# RUNX1, FUS, and ELAVL1-induced circPTPN22 promote gastric cancer cell proliferation, migration, and invasion through miR-6788-5p/PAK1 axis-mediated autophagy

**DOI:** 10.1186/s11658-024-00610-9

**Published:** 2024-07-02

**Authors:** Shuo Ma, Yanhua Xu, Xinyue Qin, Mei Tao, Xinliang Gu, Lei Shen, Yinhao Chen, Ming Zheng, Shiyi Qin, Guoqiu Wu, Shaoqing Ju

**Affiliations:** 1 Department of Laboratory Medicine, Affiliated Hospital of Nantong University, Medical School of Nantong University, Xisi Road, NO.20, Nantong, 226001 Jiangsu China; 2 Center of Clinical Laboratory Medicine, Zhongda Hospital, Medical School of Southeast University, Nanjing, 210009 Jiangsu China; 3 Diagnostics Department, Medical School of Southeast University, Nanjing, 210009 Jiangsu China; 4 Department of Laboratory Medicine, Northern Jiangsu People’s Hospital Affiliated to Yangzhou University, Yangzhou, 225000 Jiangsu China; 5 Department of Integrated Oncology, Center for Integrated Oncology (CIO), University Hospital Bonn, Bonn, Germany

**Keywords:** circPTPN22, Gastric cancer, Autophagy, miR-6788-5p, PAK1

## Abstract

**Background:**

An increasing number of studies have demonstrated the association of circular RNAs (circRNAs) with the pathological processes of various diseases and their involvement in the onset and progression of multiple cancers. Nevertheless, the functional roles and underlying mechanisms of circRNAs in the autophagy regulation of gastric cancer (GC) have not been fully elucidated.

**Methods:**

We used transmission electron microscopy and the mRFP-GFP-LC3 dual fluorescent autophagy indicator to investigate autophagy regulation. The cell counting kit-8 assay, colony formation assay, 5-ethynyl-2′-deoxyuridine incorporation assay, Transwell assay, and Western blot assay were conducted to confirm circPTPN22’s influence on GC progression. Dual luciferase reporter assays validated the binding between circPTPN22 and miR-6788-5p, as well as miR-6788-5p and p21-activated kinase-1 (*PAK1*). Functional rescue experiments assessed whether circPTPN22 modulates PAK1 expression by competitively binding miR-6788-5p, affecting autophagy and other biological processes in GC cells. We investigated the impact of circPTPN22 on in vivo GC tumors using a nude mouse xenograft model. Bioinformatics tools predicted upstream regulatory transcription factors and binding proteins of circPTPN22, while chromatin immunoprecipitation and ribonucleoprotein immunoprecipitation assays confirmed the binding status.

**Results:**

Upregulation of circPTPN22 in GC has been shown to inhibit autophagy and promote cell proliferation, migration, and invasion. Mechanistically, circPTPN22 directly binds to miR-6788-5p, subsequently regulating the expression of *PAK1*, which activates protein kinase B (Akt) and extracellular signal-regulated kinase (Erk) phosphorylation. This modulation ultimately affects autophagy levels in GC cells. Additionally, runt-related transcription factor 1 (RUNX1) negatively regulates circPTPN22 expression, while RNA-binding proteins such as FUS (fused in sarcoma) and ELAVL1 (recombinant ELAV-like protein 1) positively regulate its expression. Inhibition of the autophagy pathway can increase FUS expression, further upregulating circPTPN22 in GC cells, thereby exacerbating the progression of GC.

**Conclusion:**

Under the regulation of the transcription factor RUNX1 and RNA-binding proteins FUS and ELAVL1, circPTPN22 activates the phosphorylation of Akt and Erk through the miR-6788-5p/*PAK1* axis, thereby modulating autophagy in GC cells. Inhibition of autophagy increases FUS, which in turn upregulates circPTPN22, forming a positive feedback loop that ultimately accelerates the progression of GC.

**Supplementary Information:**

The online version contains supplementary material available at 10.1186/s11658-024-00610-9.

## Introduction

Gastric cancer (GC) represents one of the most common malignancies worldwide, ranking fifth in terms of global incidence rate. As reported by the 2020 global cancer statistics, there are almost one million new cases of GC diagnosed annually [1–3]. Despite substantial advances in surgical treatment and adjuvant therapies in recent years, the heterogeneity as well as the intricate molecular mechanisms regulating GC at the molecular level contribute to an overall dismal survival rate among patients with GC [4–6]. Notably, autophagy, a crucial cellular mechanism, exerts a pivotal role in the onset and progression of GC [7]. Recent studies have identified circular RNAs (circRNAs) as pivotal regulatory molecules of the autophagy process in GC. However, the regulatory interplay between circRNAs and autophagy as well as the clinical implications of autophagy-related circRNAs in GC diagnosis and treatment have not been thoroughly investigated. Therefore, a comprehensive investigation of the functions of circRNAs in GC initiation and progression, as well as the molecular mechanisms underlying GC cell autophagy regulation, may pave the way for new diagnostic and therapeutic strategies for GC.

CircRNAs constitute a unique category of endogenous small RNAs, distributed extensively and exhibiting diverse characteristics [8]. These covalently closed, reversal-spliced loops are predominantly formed by the circularization of exons [9, 10]. As they are distributed widely in eukaryotic cells, circRNAs possess a prolonged half-life and express in a tissue-specific and developmental stage-specific manner [10–12]. Accumulating evidence suggests that circRNAs act as microRNA (miRNA) sponges, protein decoys, RNA splicing regulators, parental gene transcription regulators, and potential protein translation templates [13]. Thus, their specific biological attributes and functions deem circRNAs as promising biomarkers for tumor diagnosis and treatment.

Autophagy is a cellular process that degrades dysfunctional components via lysosomes within the cell. In all cell types, low levels of autophagy are present in basal conditions [14]. Increasing evidence demonstrates that circRNAs can modulate autophagic activity in tumors and play a crucial role in tumor initiation and progression. For example, Gao et al. [15] reported that circPARD3 could suppress autophagy through the atypical protein kinase C isoform PRKC iota- a kinase (PRKA) C-terminal domain (Akt)-mammalian target of rapamycin (mTOR) pathway, promoting malignancy progression and chemotherapy resistance in laryngeal squamous cell carcinoma. Furthermore, As a result of circMUC16 interaction with ATG13 and miR-199a, Gan et al. [16] found that circMUC16 promotes autophagy in epithelial ovarian cancer cells. These studies support the notion that circRNAs can regulate the autophagic activity of tumors and participate in tumor-associated regulation mechanisms. However, the exploration of the regulatory mechanism of GC-specific circRNAs on autophagic capacity is still insufficient.

In this study, we conducted an in-depth investigation of the GC biomarker circPTPN22, which was selected from previous studies. Through in vitro and in vivo experiments, we discovered that circPTPN22 directly binds to miR-6788-5p, thereby regulating the expression of (*PAK1*) and activating Akt and extracellular signal-regulated kinase phosphorylation, which mediates autophagy in GC cells. Furthermore, we found that circPTPN22 regulates the proliferation, migration, and invasion of GC cells through its involvement in GC autophagy. Importantly, we identified the runt-related transcription factor 1 (RUNX1) and RNA-binding proteins (RBPs) fused in sarcoma (FUS) protein and recombinant ELAV-like protein 1 (ELAVL1) as mediators of circPTPN22 biogenesis. Finally, we have innovatively identified a feedback loop between circPTPN22 and autophagy. Upregulated circPTPN22 in GC inhibits autophagy, and this inhibitory effect leads to the upregulation of FUS, which further increases the expression of circPTPN22. This feedback loop gradually exacerbates the progression of GC. Our findings suggest that circPTPN22 can serve as a biomarker for GC and can be a potential therapeutic target.

## Materials and methods

### Human GC tissue specimens

Forty pairs of GC tissues and corresponding adjacent normal tissues were collected from patients diagnosed with GC at the Affiliated Hospital of Nantong University between December 2019 and January 2022. All patients underwent pathological analysis and had not undergone chemotherapy or surgery before tissue collection. The collected tissues were rapidly immersed in liquid nitrogen for 20 min before being stored at − 80 °C until further use. Ethics review committee approval was obtained for the study from the local hospital (ethical review report number: 2018-L055), and before enrolling in the study and publishing any subsequent findings, all participants provided informed consent.

### Total RNA extraction and quantitative real-time polymerase chain reaction (qRT-PCR)

The FastPure Cell/Tissue Total RNA Isolation Kit V2 (Vazyme Biotech Co., Ltd., Nanjing, China) was used for total RNA extraction from both GC tissues and cells. Subsequently, reverse transcription of 1000 ng of extracted total RNA was performed using a reverse transcription kit (Thermo Fisher Scientific, Waltham, MA, USA). Tissue and cell complementary DNAs were diluted by 20-fold and tenfold, respectively, in preparation for subsequent assays. For qRT-PCR detection, Q5 (Thermo Fisher Scientific) was used, and GADPH served as an internal reference for circPTPN22 and messenger RNA (mRNA), whereas U6 was used as an internal reference for miR-6788-5p. The polymerase chain reaction primer sequences are provided in Table S1. Analysis was performed with the 2^−ΔΔCT^ method.

### Cell culture and plasmid transfection

Cell lines of human gastric epithelial cells (GES-1) and GC cells (AGS, SGC-7901, MKN-45, and MKN-1) were obtained from the Chinese Academy of Sciences (Shanghai, China). Cells were cultured on Roswell Park Memorial Institute 1640 (Corning, NY, USA) medium supplemented with 10% fetal bovine serum (Gibco, Thermo Fisher Scientific) and 1% double-antibody (New Cell & Molecular Biotech Co., Ltd., Suzhou, China) at 37 °C in a CO_2_ incubator humidified with 5% CO_2_. The medium was replaced every 2 days. The lentiviral short hairpin RNA (shRNA) interference vector targeting circPTPN22 (LV-sh-circPTPN22) was purchased from Geneseed Biotech Co., Ltd. (Guangzhou, China). The circPTPN22 overexpression vector (pc-circPTPN22) and other mRNA shRNA interference vectors and overexpression vectors were synthesized by Genepharma Technology Co., Ltd. (Suzhou, China). The mimic and inhibitor of miR-6788-5p were synthesized by Ruibo Biotechnology Co., Ltd. (Guangzhou, China). GC cells were transfected with Lipofectamine 3000 (Invitrogen, Carlsbad, CA, USA). After a 48-h transfection, cells were collected for subsequent experiments. The sequences used are detailed in Table S2.

### Cell counting kit 8 (CCK-8) assay and cell colony formation assay

The cellular experiments involved the seeding of cells into 96-well plates at a density of approximately 3000 cells per well after 48 h of transfection, with each group of fragments set up in five parallel wells. Subsequently, 10 μL of CCK-8 reagent from Dojindo, Osaka, Japan, was added to each well and incubated for a period of 5 consecutive days. Daily absorbance measurements were carried out using a microplate reader at 450 and 630 nm, and the difference in absorbance between the two wavelengths was calculated. This protocol allowed for the assessment of cell viability and proliferation over the 5-day period.

Furthermore, for the colony formation assay, the remaining cell suspension from the CCK-8 assay was added to a 6-well plate at a density of approximately 800–1000 cells per well and cultured for a duration of 14 days. Upon the observation of visible cell clusters in the wells, the supernatant culture solution was discarded, and the cells were subjected to 2–3 washes with phosphate-buffered saline (PBS). Following this, the cells were fixed, and images were captured under a microscope to analyze and record the colony formation.

### 5-Ethynyl-2′-deoxyuridine (EdU) incorporation assay

The transfected cells were seeded into a 24-well plate to ensure their uniform distribution. Once the cells had adhered to the plate, the culture medium was replaced with a medium containing 50 μM EdU and incubated. For cell staining, Apolo and Hoechst 33342 were utilized. The fluorescence intensity of the cells was observed under a fluorescence microscope, and five random fields were selected for imaging and analysis. The positive rate of cells exhibiting proliferative ability was then calculated.

### Transwell assay

During the transwell migration and invasion assays, 500 μL of complete culture medium was filled into 24-well plates. Subsequently, a transwell chamber was inserted into each well. For the invasion assay, Matrigel and the base culture medium were mixed at a 1:6 ratio and added to the chamber. Cell counting was performed using a hemocytometer, and the prepared cell suspension was added to each chamber. After 48–72 h of incubation, the cells were fixed, stained, and washed with PBS. Five random fields were chosen for imaging and recording using a microscope.

### Transmission electron microscopy (TEM)

The culture medium was aspirated from the treated cells, and the cells were harvested and fixed in 2% glutaraldehyde. The mixture was then placed and fixed overnight in a 4 °C refrigerator. Following this, it was incubated in 1% osmium tetroxide at 4 °C for 1 h, dehydrated in a series of graded ethanol, infiltrated in graded acetone, sectioned into 50 nm sections, and then stained with lead citrate. Subsequently, five random fields were chosen for imaging and analysis.

### mRFP-GFP-LC3 dual fluorescent autophagy indicator system

After the third cell passage, cells were plated on slides and transfected with the mRFP-GFP-LC3 plasmid (Hanyin Biotechnology Co., Ltd., Shanghai, China) once the confluency reached 60–70%. After 48 h, the corresponding knockdown or overexpression plasmids were transfected. Following an additional 48 h of standard cell culture, cell fluorescence was observed. When significant fluorescence was present, the cells were fixed, washed with PBS 2–3 times, and imaged under a microscope. Five random fields were selected for image collection and analysis.

### Western blot assay

The protein extraction process involved the use of radioimmunoprecipitation assay buffer, which contained protease and phosphatase inhibitors from SolarBio Life Science, Beijing, China. Following extraction, the quantified proteins were separated using sodium dodecyl-sulfate polyacrylamide gel electrophoresis, with concentrations ranging from 7.5% to 15%. Subsequently, the separated proteins were transferred to polyvinylidene difluoride membranes from Millipore, Billerica, MA, USA. These membranes were then blocked using Rapid Blocking Solution from EpiZyme Biotechnology, Shanghai, China, and incubated overnight at 4 °C with the corresponding primary antibody. After washing, the membranes underwent incubation with the corresponding secondary antibody. Notably, antibodies against P62/SQSTM1, LC3, β-actin, E-cadherin (E-cad), vimentin, PAK1, p-Akt (s473), Akt, p-Erk (Thr202/Thr204), Erk, RUNX1, FUS, ELAVL1 and PTPN22 were sourced from Cell Signaling Technology, based in Danvers, MA, USA.

### Fluorescence in situ hybridization (FISH)

Specific probes for circPTPN22 (Ruibo Biotechnology Co., Ltd.) and miR-6788-5p (Genepharma Technology Co., Ltd.) were synthesized. FISH detection was conducted using a FISH kit (Ruibo Biotechnology Co., Ltd.) following the manufacturer’s instructions. Cells were fixed with a fixative and permeabilized with PBS containing 0.5% Triton X-100. A hybridization solution containing circPTPN22, miR-6788-5p, 18s, and U6 probes was added to each well and incubated overnight. After incubation, slides were washed three times with saline-sodium citrate buffer and then stained with Hoechst 33342 for 4′,6-diamidino-2-phenylindole (DAPI) staining. The slides were then fixed, and images were captured using a fluorescence microscope.

### The nude mouse xenograft model

Female BALB/c nude mice (4-week-old) were purchased from the Animal Center of Nantong University, Nantong, China. All animal experimental procedures were approved by the Animal Use and Care Committee of Nantong University (ethical review report number: S20230901-004). LV-NC and LV-sh-circPTPN22 lentiviruses were transfected into AGS cells. Subsequently, the stable LV-NC and LV-sh-circPTPN22-infected AGS cell suspensions were mixed with a pre-prepared 50 μL matrix gel and subcutaneously implanted. After successful tumor growth for 9 days, the miR-6788-5p inhibitor and PAK1 overexpression plasmid were injected around the subcutaneous tumors every 3 days until the end of the experiment. Measurements of tumor size were taken every two days, and tumor volume was calculated using V = 0.5 × length (a) × width (b)^2^, and a growth curve was plotted. On day 24 after cell implantation, the tumors were excised from the nude mice and weighed. A part of the excised tumor was fixed for immunohistochemical and immunofluorescence (IF) staining, while another part was immediately frozen at − 80 °C for further experiments.

### Immunohistochemistry (IHC) and IF

In order to perform IHC, tumor tissues were first treated with 4% paraformaldehyde, encapsulated in paraffin, and sectioned at a thickness of 5 μM. Subsequently, a 20 min incubation at 95 °C with sodium citrate buffer (pH 6.0) was followed by a 10 min blocking step with 5% normal goat serum at 20 °C in order to retrieve antigens. Afterward, monoclonal antibodies against E-cad, vimentin, or Ki-67 were incubated overnight at 4 °C, followed by secondary antibodies.

IF included sections of 5 μM thickness from the paraffin-embedded tumor tissues. For antigen retrieval, 0.01 M citrate buffer (pH 6.0) was used for 20 min in a pressure cooker. Fixed immunofluorescent cells were permeabilized with 0.05% Triton X-100 in PBS on ice for 5 min after being fixed with 4% paraformaldehyde for 20 min. The samples were then blocked with PBS containing 2% bovine serum albumin at RT for 1 h and incubated overnight at 4 °C with primary antibodies against LC3 and P62/SQSTM1. Fluorescent-conjugated or enzyme-conjugated secondary antibodies were then incubated at RT for 1 h. Multiplex fluorescence immunostaining was performed using the Multiplex Fluorescent Immunohistochemistry Staining Kit (abs50013, Absin, Shanghai, China). Finally, the cell nuclei were counterstained with DAPI. The tissue sections were then scanned. P62/SQSTM1, LC3, E-cad, and Vimentin were all purchased from Cell Signaling Technology, and Ki67 was purchased from Abcam (Burlingame, CA, USA).

### Bioinformatics analysis

Using the TargetScan, the circBank website, miRanda, and RNAhybrid software to predict miRNAs combined with circPTPN22, nine miRNAs were obtained after cross-analysis.

The Circinteractome website was used to predict the RBPs combined with circPTPN22. The circRNADb website was used to predict the open reading frame (ORF), internal ribosome entry site (IRES), and m6A sites contained in circPTPN22.

Using Targetscan, miRWalk, miRPathDB, the miRDB website, and miRanda software to predict the mRNAs combined with miR-6788-5p. After the intersection analysis, 69 mRNAs were obtained, and then the 69 mRNAs were combined with the Pathcards website. Cross-analysis of mRNAs with autophagy-related proteins yielded 2 mRNAs.

The JASPAR website was used to predict transcription factors that bind to the PTPN22 promoter region. Using circAtlas, RBPDB, and Starbase website prediction, the results were obtained after combining RBP with pre-PTPN22 and cross-analysis. The websites involved are listed in Table S3.

### Dual-luciferase reporter assay

Vazyme Biotech Co. Ltd. provided the Dual-Luciferase Reporter Assay System (Vazyme Biotech Co. Ltd.) for conducting this assay. Wild-type (WT) and mutant (Mut) reporter genes for circPTPN22 (Ruibo Biotechnology Co., Ltd.), PAK1, and RUNX1 (Geneseed Biotech Co., Ltd.) were constructed. Next, GC cells were co-transfected with a reporter gene and either 100 nM miR-6788-5p or 100 nM miR-NC and pc-PTPN22 plasmid using Lipofectamine 3000 (Invitrogen) in 24-well plates.

### RNA immunoprecipitation (RIP)

RIP detection was performed using a RIP kit (BersinBio, Guangzhou, China) following the manufacturer’s instructions. Sample cells were lysed with lysis buffer containing protease and ribonuclease inhibitors. Magnetic beads were pre-incubated with either anti-AGO2, anti-FUS, anti-ELAVL1 antibodies (Cell Signaling Technology), or anti-immunoglobulin G (IgG) antibodies for 1 h at RT, followed by overnight IP of the lysate at 4 °C. Total RNA was then extracted from the sample, and the enrichment level of the corresponding RNA was detected.

### CircRNA pulldown assay

A biotinylated probe specific to circPTPN22 was synthesized (Ruibo Biotechnology Co., Ltd.). Cells were lysed with IP lysis buffer supplemented with protease inhibitors and RNase inhibitors (Thermo Fisher Scientific) for 30 min on ice. The lysate was sonicated for 2 min and pre-cleared with 30 μL of agarose beads, rotating for 1 h at 4 °C. Subsequently, a biotin-labeled probe specific to circPTPN22 or a negative control probe was added to the pre-cleared lysate, and the mixture was incubated overnight at 4 °C. Then, 30 μL of streptomycin agarose beads were incubated at 37 °C for 1 h, followed by washing with 1 mL of IP lysis buffer. To detect circPTPN22 and miRNAs, total RNA bound to the beads was extracted for reverse transcription and qRT-PCR analysis.

### Chromatin IP (ChIP)

ChIP assays were performed using a ChIP kit (Millipore). Cells were cross-linked with 1% formaldehyde, and then an appropriate amount of glycine was added to terminate the cross-linking reaction. The resulting DNA–protein complexes were sonicated to generate DNA fragments ranging from 200 to 1000 bp. Subsequently, a CHIP-grade antibody specific to RUNX1 (Abcam) was added to form antibody-target protein-DNA complexes, followed by IP using protein agarose beads. After washing and reverse cross-linking, the enriched DNA was purified for subsequent detection.

### Statistical analysis

In this study, statistical analysis was conducted primarily using GraphPad Prism 8.0 (GraphPad Software Inc., CA, USA) and SPSS 23.0 (IBM, SPSS, Chicago, IL, USA). The t-test was used for comparison of data between two groups in the experimental operations, whereas one-way analysis of variance was used for comparing data among more than two groups. An analysis of survival curves was performed using Kaplan–Meier (K–M) methods and a log-rank test to compare them. A *p*-value < 0.05 was considered statistically significant.

## Results

### circPTPN22 can inhibit GC cell autophagy

**Fig. 1:**
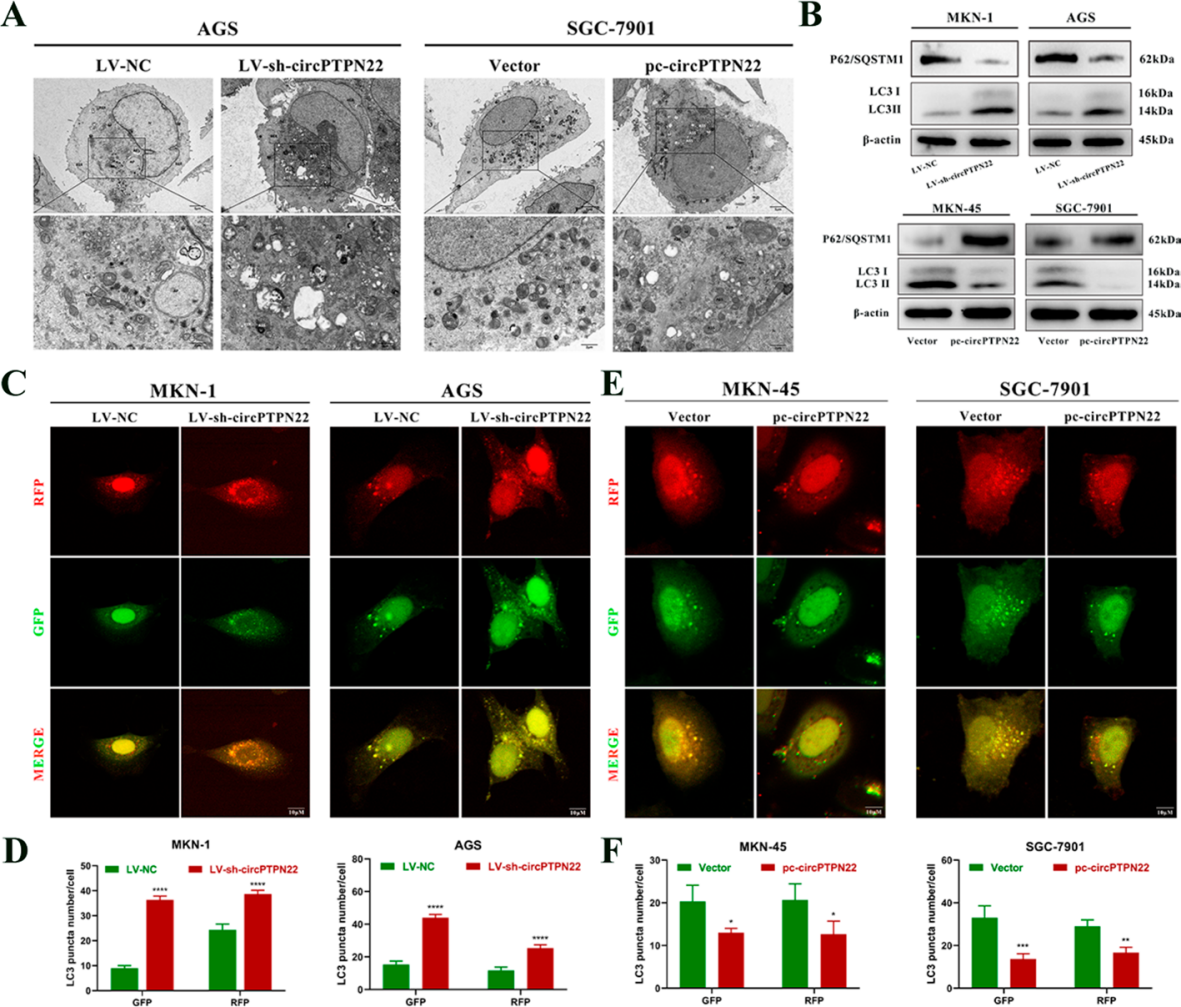
circPTPN22 inhibits autophagy in GC cells. **A** Transmission electron microscope observation of the number of autophagosomes and autolysosomes in GC cells after knockdown and overexpression of circPTPN22. Scale bars are 5 μM and 2 μM, respectively. **B** Western blot assay to detect the protein levels of P62/SQSTM1 and LC3 B in GC cells after knockdown and overexpression of circPTPN22. **C–F** circPTPN22 was knocked down in mRFP-GFP-LC3-labeled MKN-1 and AGS cells, and circPTPN22 was overexpressed in MKN-45 and SGC-7901 cells. Confocal microscopy was used to observe the autophagic flux. **C **and** E** are representative confocal microscope images, and **D **and** F** are quantitative data. Scale bar, 10 μM. *p < 0.05, **p < 0.01, ***p < 0.001, ****p < 0.001

The previous report on circPTPN22’s potential as a diagnostic marker for GC and its ability to monitor patient prognosis set the stage for the current study. In the present research, a lentivirus targeting circPTPN22 was engineered and used to achieve effective knockdown in MKN-1 and AGS cells. Subsequently, the results were validated through in vitro cell function assays, including CCK-8, EdU, and Transwell assays. The study’s findings revealed that the knockdown of circPTPN22 led to a significant inhibition of proliferation, migration, and invasion of GC cells, as demonstrated in Figure S1A, C, E, F, I, J, M, and N. Furthermore, the transfection of circPTPN22 overexpression plasmids in MKN-45 and SGC-7901 cells yielded opposite results compared to the knockdown, as indicated in Figure S1B, D, G, H, K, L, O, and P. These results highlight circPTPN22’s crucial role in modulating the behavior of GC cells, specifically in promoting proliferation, migration, and invasion. The use of lentiviral knockdown and overexpression plasmids in tandem with in vitro cell function assays provides robust evidence for the functional impact of circPTPN22 in GC cells, laying the groundwork for further understanding its molecular mechanisms and potential therapeutic interventions. Consequently, we were interested in understanding the specific mechanism behind these findings. Previous studies have revealed that circRNAs could regulate various cellular processes by inducing autophagy [17, 18]. Hence, we speculated whether circPTPN22 induced autophagy in GC cells. Our findings showed that, compared with the LV-NC group, the number of autophagosomes increased following the knockdown of circPTPN22, and a certain number of autolysosomes were observed. Autophagosome formation decreased when circPTPN22 was overexpressed, and only a few autolysosomes appeared (Fig. 1A). Autophagy activation or inhibition resulted in altered expression of P62 and LC3 II as well as autophagic flux. Therefore, we used Western blot assays and the mRFP-GFP-LC3 dual fluorescent autophagy indicator system to examine changes in autophagy marker protein expression and autophagy flux in GC cells. The results showed that the knockdown of circPTPN22 reduced the expression of P62 but increased the expression of LC3 II in MKN-1 and AGS cells (Fig. 1B). Moreover, autophagosomes gradually evolved into autolysosomes, and the number of autophagosomes increased in GC cells (Fig. 1C and D). In contrast, overexpression of circPTPN22 resulted in the opposite findings (Fig. 1B, E, and F). Collectively, our findings suggest that highly expressed circPTPN22 could inhibit the autophagy of GC cells.

### circPTPN22 promotes the progression of GC cells by mediating autophagy

**Fig. 2:**
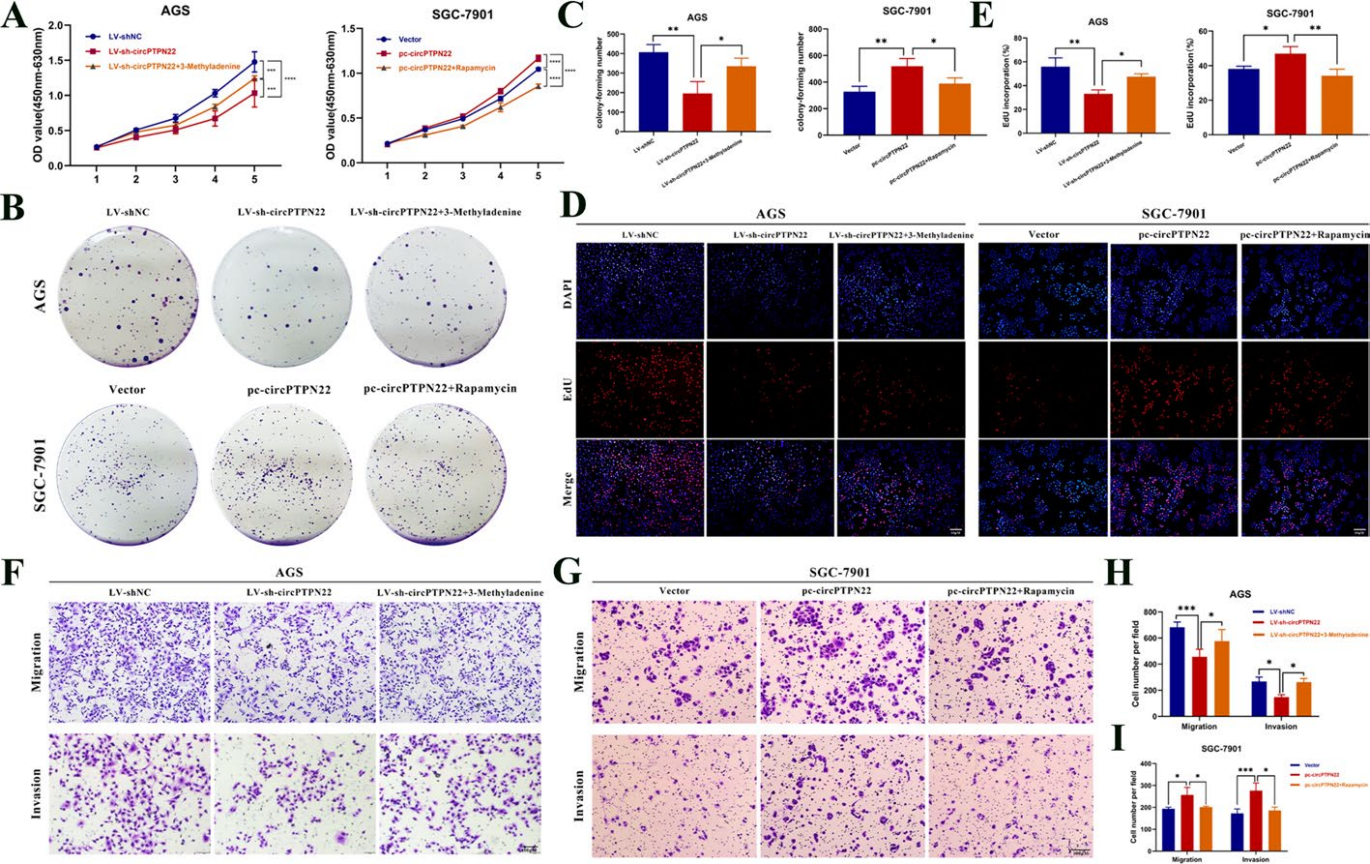
Autophagy can restore the promoting effect of circPTPN22 on GC. **A–E** circPTPN22 knockdown or overexpression GC cells were treated with autophagy inhibitor 3-Methyladenine or autophagy activator Rapamycin for 24 h after 48 h. The proliferation of GC cells was detected by CCK-8 assay (**A**), cell colony formation assay (**B**) and EdU cell proliferation assay (**D**). **B **and** D** are representative clones and fluorescence images, and **C **and** E** are quantitative data. Scale bar, 100 μM. **F–I** circPTPN22 knockdown or overexpression GC cells were treated with autophagy inhibitor 3-Methyladenine or autophagy activator Rapamycin for 24 h after 48 h, and the migration (**F**) and invasion (**G**) of GC cells were detected by transwell assay. **F **and** G** are representative migration and invasion micrographs, and **H **and** I** are quantitative data. Scale bar, 100 μM. *p < 0.05, **p < 0.01, ****p < 0.001

To investigate whether circPTPN22 regulates the progression of GC cells by mediating autophagy, we knocked down or overexpressed circPTPN22 in AGS and SGC-7901 cells. The treated cells were then exposed to the autophagy inhibitor 3-Methyladenine (3-MA) and the autophagy inducer Rapamycin (Rap), followed by in vitro recovery assays. CCK-8, cell colony formation, and EdU rescue experiments revealed that inhibition of autophagy partially restored the suppressed cell proliferation observed after circPTPN22 knockdown, whereas enhanced autophagy restored the promoting effects of circPTPN22 overexpression on GC cell proliferation (Fig. 2A–E). Similarly, in Transwell rescue experiments, the reduction of autophagy partially reversed the observed inhibition of cell migration and invasion after knockdown of circPTPN22, while enhanced autophagy restored the promoting effects of circPTPN22 overexpression on the migration and invasion of GC cells (Fig. 2F–I). These results indirectly implicate circPTPN22 in the progression of GC through its influence on autophagy.

### circPTPN22 acts as a sponge for miR-6788-5p in GC cells

**Fig. 3:**
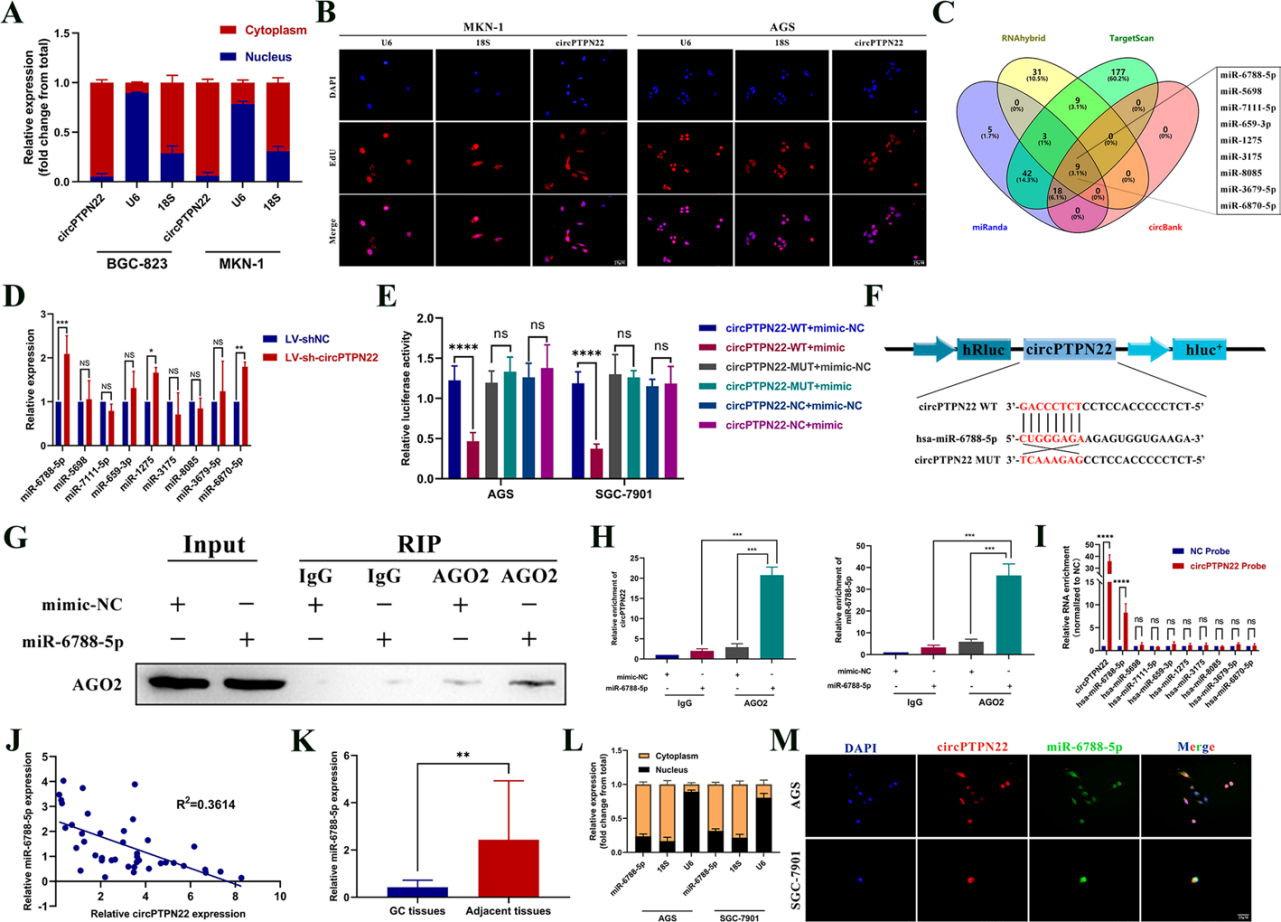
circPTPN22 can directly bind miR-6788-5p. **A **and** B** Nuclear separation assay (**A**) and FISH assay (**B**) to detect the sublocalization of circPTPN22 in GC cells. Scale bar, 25 μM. **C** Bioinformatics detection of target miRNAs of circPTPN22. **D** qRT-PCR assay to detect the expression of miRNAs in GC cells after knockdown of circPTPN22. **E **and** F** Dual luciferase reporter gene assay to detect the binding of circPTPN22 and miR-6788-5p. **G** Anti-AGO2 RIP analysis of AGO2 protein expression detected by Western blotting. **H** qRT-PCR assay to detect the expression of circPTPN22 and miR-6788-5p. **I** RNA pulldown assay was performed in GC cells using biotin-labeled circPTPN22 probe, and then the enrichment of the indicated miRNAs was detected by qPCR analysis. **J** Correlation analysis of expression of circPTPN22 and miRNAs in 40 pairs of GC tissues. **K** Expression of miR-6788-5p in GC tissues. **L** Nuclear separation assay to detect the sublocalization of miR-6788-5p in GC cells. **M** FISH colocalization assay to detect the cellular sublocalization of circPTPN22 and miR-6788-5p. Scale bar, 25 μM. *p < 0.05, **p < 0.01, ***p < 0.001, ****p < 0.001

To elucidate the mechanism by which circPTPN22 regulates autophagy and biological functions in GC, we first determined the subcellular localization of circPTPN22 using nuclear-cytoplasmic separation and FISH assays, which revealed that circPTPN22 was predominantly located in the cytoplasm (Fig. 3A and B). Currently, circRNAs can exert their functions in disease states by acting as miRNA sponges, RBPs, or templates for translation [19]. Following this, we used bioinformatics methods to identify the miRNAs, RBP, and IRES sites that circPTPN22 may bind to. It was observed that circPTPN22 bound fewer RBPs and had a lower binding site score (Figure S2A). Additionally, circPTPN22 contained only IRES sites but lacked both m6A sites and ORF, which reduces the likelihood of circPTPN22 being a translation template (Figure S2B). Based on these observations, we hypothesized that circPTPN22 regulates GC autophagy by acting as a miRNA sponge. Through bioinformatics analysis, we identified nine miRNAs that circPTPN22 is most likely to bind to (Fig. 3C). From these, we selected miR-6788-5p for further analysis since its expression was significantly upregulated upon circPTPN22 knockdown (Fig. 3D). Further dual-luciferase reporter assays revealed that miR-6788-5p mimics only inhibited circPTPN22 WT vector luciferase activity, but not Mut vector luciferase activity (Fig. 3E and F). According to previous studies, the binding of circRNAs and miRNAs relies on AGO2, which mediates their interactions [20]. Our anti-AGO2 RIP assay in AGS cells confirmed the relationship between circPTPN22 and miR-6788-5p in GC cells. Our results showed that the anti-AGO2 antibody enriched AGO2 in AGS cells transfected with miR-6788-5p mimic but not in the negative control (IgG) (Fig. 3G). According to qRT-PCR results, AGS cells transfected with miR-6788-5p mimic were significantly more enriched for circPTPN22 and miR-6788-5p than mimic-NC cells (Fig. 3H). Pulldown assays also revealed that the circPTPN22 probe effectively enriched miR-6788-5p, which was not observed in the control group (Fig. 3I). Spearman correlation analysis indicated that the expression levels of miR-6788-5p and circPTPN22 were negatively correlated (Fig. 3J). We further analyzed the expression of miR-6788-5p and observed its downregulation in both GC tissues and cells (Fig. 3K and Figure S2C). Furthermore, FISH co-localization and nuclear-cytoplasmic separation assays showed that both molecules co-exist in the cytoplasm (Fig. 3L and M), thereby confirming that circPTPN22 could act as a miRNA sponge for miR-6788-5p. Additionally, an analysis of K-M curves showed that patients with high miR-6788-5p expression had a better chance of surviving (Figure S2D).

### miR-6788-5p can partially restore the effect of circPTPN22 on inhibiting autophagy

**Fig. 4:**
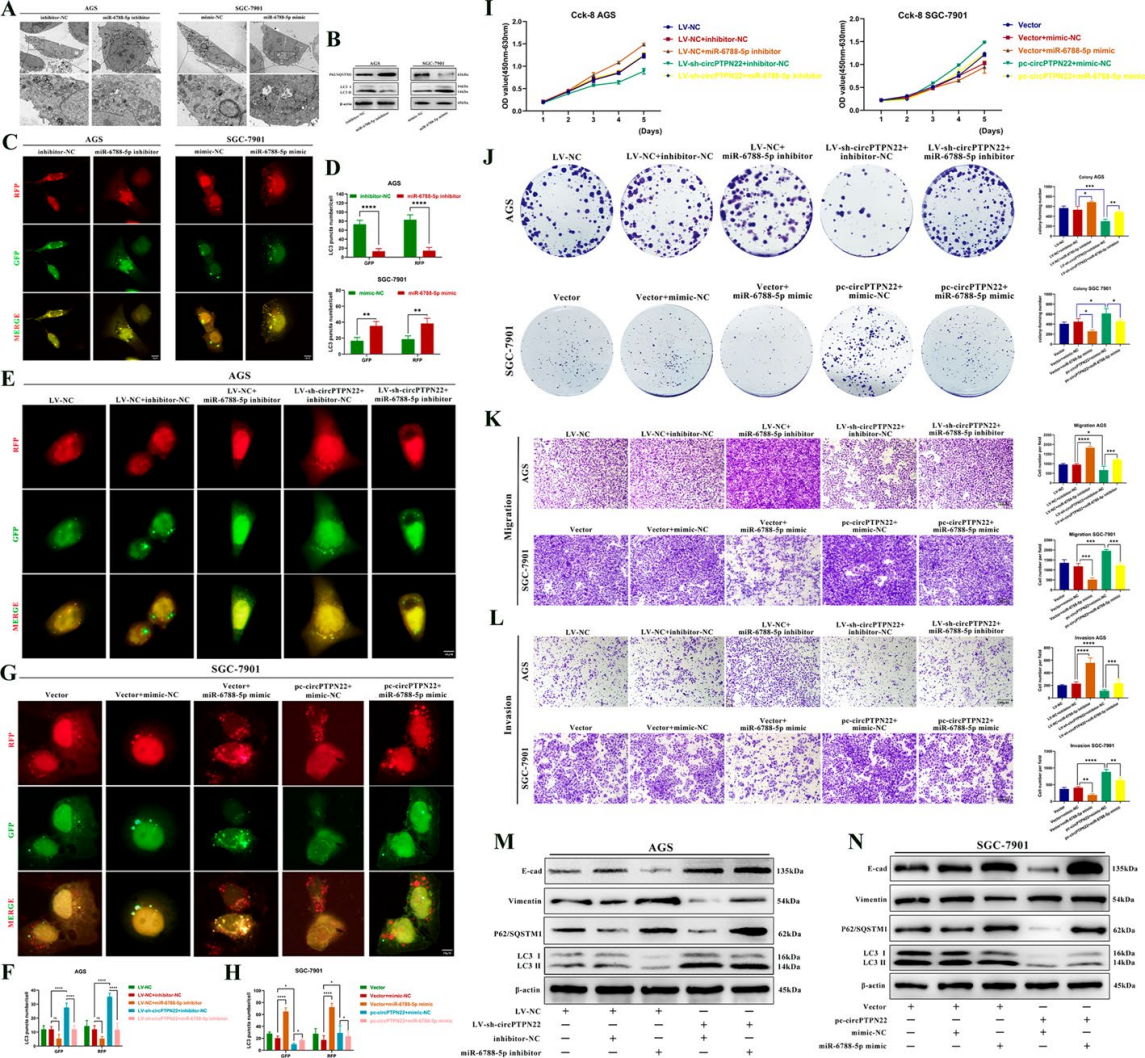
miR-6788-5p can partially restore the effects of circPTPN22 on autophagy inhibition. **A** Transmission electron microscope observation of the number of autophagosomes and autolysosomes in GC cells after adding miR-6788-5p mimic or inhibitor. **B** Western blot detection of P62/SQSTM1 and LC3 B protein levels in GC cells after adding miR-6788-5p mimic or inhibitor. **C **and** D** miR-6788-5p inhibitor and mimic were added to mRFP-GFP-LC3-labeled AGS and SGC-7901 cells, respectively. Cellular autophagic flux was visualized using confocal microscopy. **C** is a representative confocal microscope image, and **D** is quantitative data. Scale bar, 10 μM. **E–H** After knockdown or overexpression of circPTPN22 in mRFP-GFP-LC3-labeled GC cells, the inhibitor or mimic of miR-6788-5p was added, and the autophagy flux was observed by confocal microscopy. **E **and** G** are representative confocal microscope images, and **F **and** H** are quantitative data. Scale bar, 10 μM. **I–L** After knockdown or overexpression of circPTPN22, inhibitor or mimic of miR-6788-5p was added. The proliferation and metastasis of GC cells were detected by CCK-8 (**I**), cell colony formation assay (**J**), migration (**K**) and invasion (**L**) assays. Scale bar, 100 μM. **M **and** N** After knockdown or overexpression of circPTPN22, inhibitor or mimic of miR-6788-5p was added. Western blot was used to detect the protein content of E-cad, vimentin, P62/SQSTM1 and LC3 B in GC cells. *p < 0.05, **p < 0.01, ***p < 0.001, ****p < 0.001

Initially, we performed in vitro functional assays to investigate the effects of miR-6788-5p on autophagy and other biological functions in GC. TEM, Western blot assay, and the mRFP-GFP-LC3 double fluorescence autophagy indicator system demonstrated that the miR-6788-5p inhibitor reduced the number of autophagosomes and autolysosomes, increased P62 expression, and decreased LC3 II expression in GC cells. Conversely, an opposite effect was observed upon the addition of the miR-6788-5p mimic (Fig. 4A–D). In summary, miR-6788-5p, a tumor suppressor gene, is capable of promoting autophagy in GC cells. Additionally, in vitro functional assays indicated that, compared to the control group, the proliferation, migration, and invasion abilities of GC cells were enhanced with the addition of the miR-6788-5p inhibitor, whereas the opposite results were observed with the addition of the miR-6788-5p mimic (Figure S3A-H). Furthermore, we found that the effects of miR-6788-5p on GC cells could be reversed by altering autophagy (Figure S3H). These findings suggest that miR-6788-5p can inhibit the progression of GC by regulating autophagy.

We then conducted a partial rescue experiment to explore whether circPTPN22 influences GC cells by binding to miR-6788-5p. Following the knockdown of circPTPN22 in AGS cells and the addition of the miR-6788-5p inhibitor, mRFP-GFP-LC3 double fluorescent autophagy indicator system transfection rescue experiments revealed that the inhibitor partially restored the increased number of autophagosomes and autolysosomes in GC cells after circPTPN22 interference (Fig. 4E and F). Additionally, upon overexpression of circPTPN22 in SGC-7901 cells, the miR-6788-5p mimic was added, which partially restored the reduction in the number of autophagosomes and autolysosomes in GC cells (Fig. 4G and H). Western blot analysis showed that the miR-6788-5p inhibitor restored a decrease in P62 expression and an increase in LC3 II expression after circPTPN22 knockdown. Moreover, miR-6788-5p mimics exert contrasting effects (Fig. 4M and N). These findings suggest that miR-6788-5p can partially restore the effects of circPTPN22 on autophagy in GC cells. Additionally, in vitro experiments have demonstrated that miR-6788-5p inhibitors or mimics can reverse the effects induced by knockdown or overexpression of circPTPN22 in GC cells (Fig. 4I–L).

### *PAK1* is a downstream target gene of miR-6788-5p

**Fig. 5:**
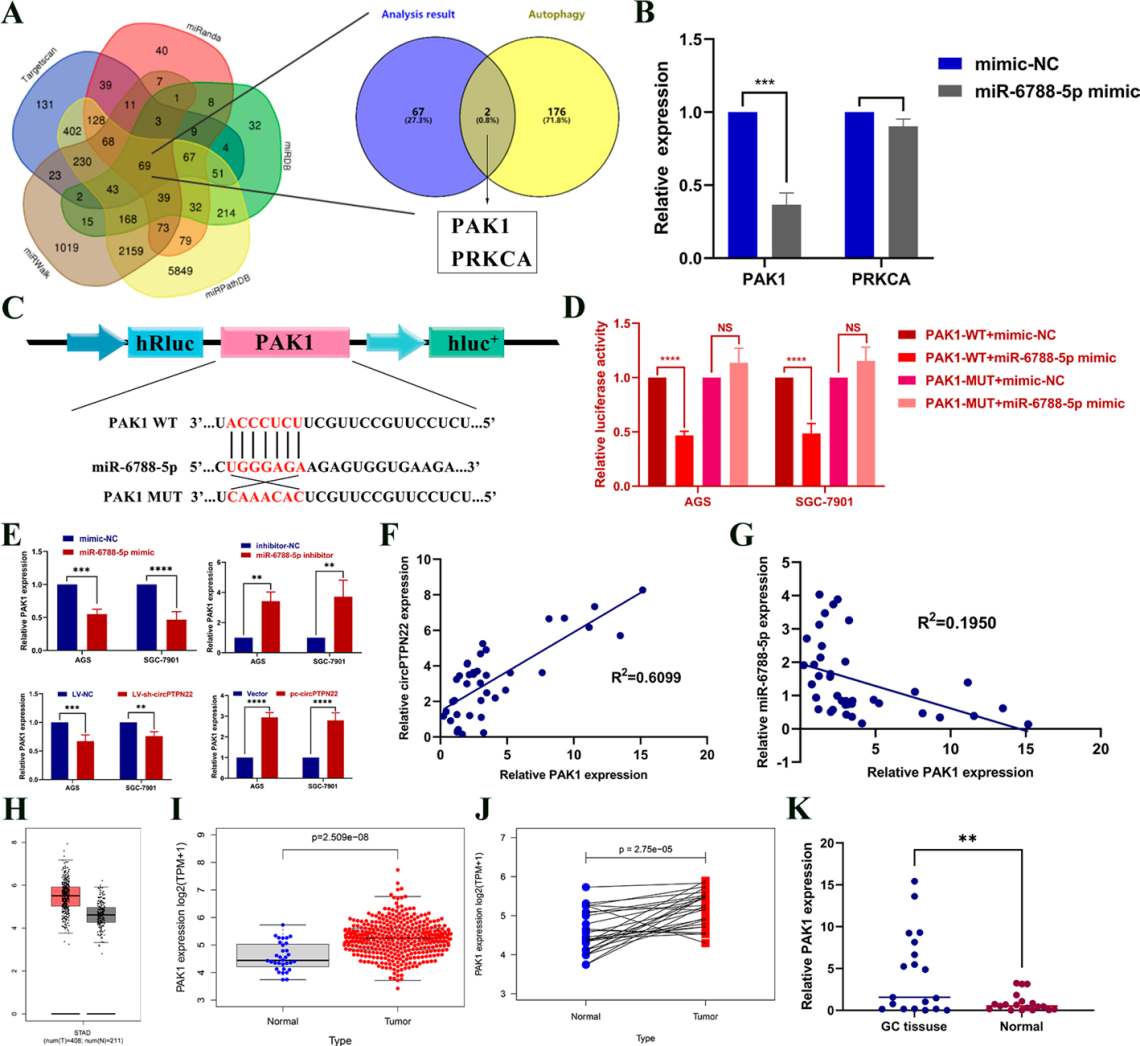
PAK1 can directly bind miR-6788-5p. **A** Bioinformatics analysis of downstream mRNAs associated with autophagy bound by miR-6788-5p. **B** qRT-PCR assay to detect the expression levels of PAK1 and PRKCA in GC cells after adding miR-6788-5p mimic. **C **and** D** Dual luciferase reporter gene assay to detect the binding of PAK1 and miR-6788-5p. **E** Expression of PAK1 after adding miR-6788-5p inhibitor and mimic or knocking down and overexpressing circPTPN22 in GC cells. **F **and** G** Correlation analysis of expression of PAK1 and miR-6788-5p, PAK1 and circPTPN22 in 40 pairs of GC tissues. **H** Expression of PAK1 in GC and normal tissues in the GEPIA database. **I **and** J** Expression of PAK1 in GC tissues and paired normal tissues from the TCGA database. **K** Expression of PAK1 in GC tissues. **p < 0.01, ***p < 0.001, ****p < 0.001

In our investigation of miR-6788-5p downstream targets, 69 mRNAs were identified through bioinformatics analysis. These were further scrutinized concerning autophagy pathway-related genes, leading to the identification of *PAK1* and *PRKCA* genes (Fig. 5A). The expression level of only *PAK1* demonstrated significant downregulation upon the addition of miR-6788-5p mimic to cells (Fig. 5B), rendering it the focus of subsequent studies. Using a dual-luciferase reporter assay, we found that miR-6788-5p mimic inhibited *PAK1* WT luciferase activity but exerted no significant effect on *PAK1* Mut luciferase activity (Fig. 5C and D), indicating that *PAK1* could bind to miR-6788-5p. qRT-PCR analysis confirmed that alterations in the expression levels of miR-6788-5p or circPTPN22 can lead to changes in *PAK1* mRNA expression levels (Fig. 5E). Further correlation analysis revealed that the expression level of PAK1 is inversely correlated with the expression level of miR-6788-5p, and positively correlated with the expression level of circPTPN22 (Fig. 5F and G). In contrast to GES-1 cells, GC cell lines exhibited higher levels of *PAK1* expression (Figure S4), and it is upregulated in GC tissues. (Fig. 5H–J). Additionally, we examined *PAK1* expression levels in 40 pairs of GC tissues and established relatively high *PAK1* expression in GC tissues (Fig. 5K). These results suggest that *PAK1* can be targeted by miR-6788-5p and induced by circPTPN22.

### PAK1 can partially restore the effects of circPTPN22 on autophagy inhibition

**Fig. 6:**
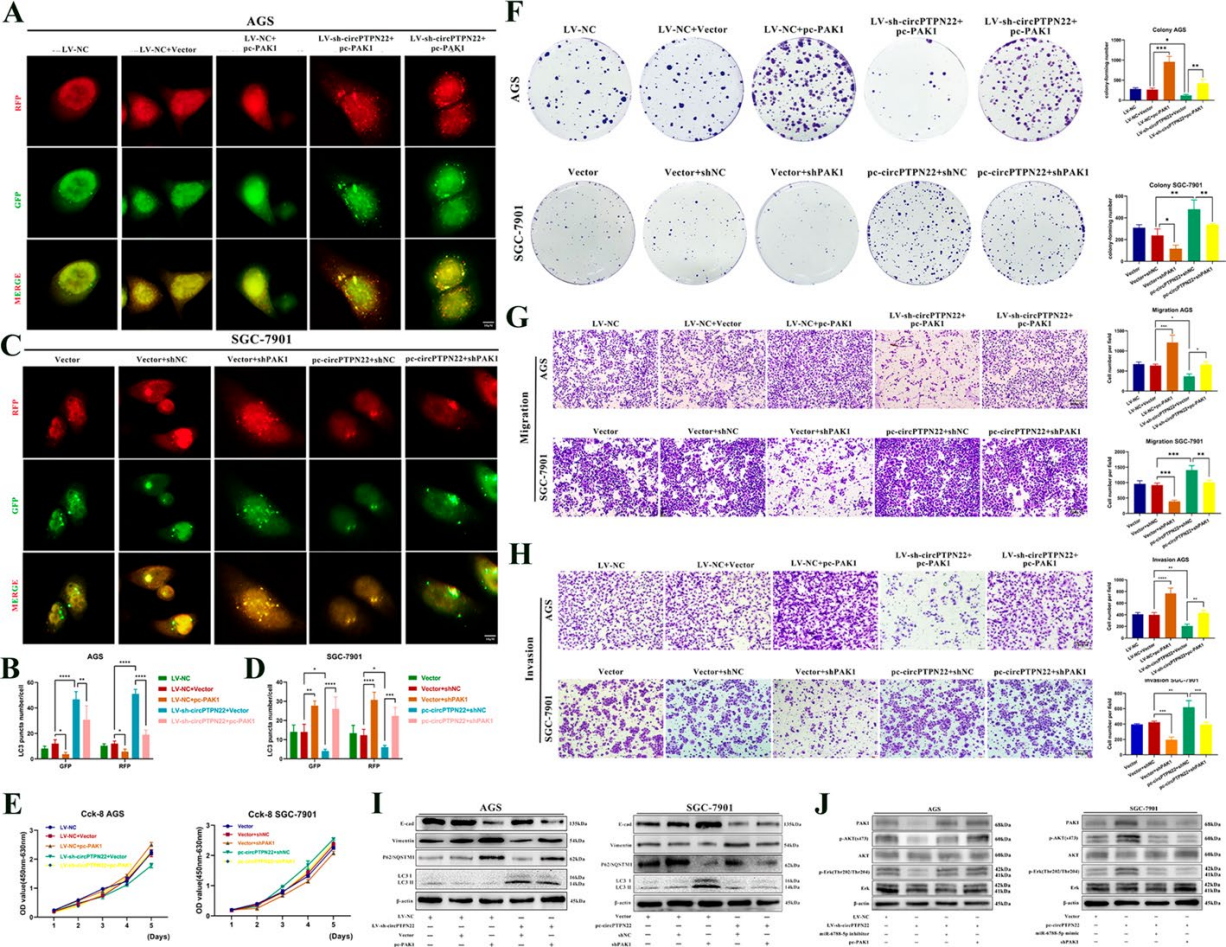
The circPTPN22/miR-6788-5p/PAK1 axis regulates autophagy in GC cells by activating AKT and ERK phosphorylation. **A–D** After knockdown or overexpression of circPTPN22 in mRFP-GFP-LC3-labeled GC cells, PAK1 knockdown or overexpression plasmids were added. Cellular autophagic flux was visualized by confocal microscopy. **A **and** C** are representative confocal microscope images, and **B **and** D** are quantitative data. Scale bar, 10 μM. **E–H** After knockdown or overexpression of circPTPN22, inhibitor or mimic of miR-6788-5p was added. The proliferation and metastasis of GC cells were detected by CCK-8 (**E**), cell colony formation assay (**F**), migration (**G**) and invasion (**H**) assays. Scale bar, 100 μM. **I** After knocking down or overexpressing circPTPN22, add miR-6788-5p inhibitor or mimic. Western blot was used to detect the protein content of E-cad, vimentin, P62/SQSTM1 and LC3 B in GC cells. **J** Add miR-6788-5p inhibitor or pc-PAK1 after knocking down circPTPN22 and add miR-6788-5p mimic or shPAK1 after overexpressing circPTPN22. The protein levels of PAK1, p-AKT (s473), AKT, p-Erk (Thr202/Thr204) and Erk in GC cells were detected by Western blot. *p < 0.05, **p < 0.01, ***p < 0.001, ****p < 0.001

To investigate whether PAK1 can reverse the effects of circPTPN22 on autophagy in GC cells, we knocked down circPTPN22 in AGS cells and overexpressed PAK1 using a corresponding vector. Using a mRFP-GFP-LC3 double fluorescent autophagy indicator system transfection and Western blot rescue experimentation, we discovered that PAK1 overexpression could only partially alleviate the increase in autophagosomes and autophagic lysosomes, the reduction in P62 expression, and the increase in LC3-II expression in GC cells after circPTPN22 knockdown (Fig. 6A, B, and I). Conversely, interference with PAK1 led to the opposite results (Fig. 6C, D, and I). Furthermore, in vitro experiments have shown that knocking down or overexpressing PAK1 can effectively restore the biological effects of overexpression or knockdown of circPTPN22 on GC cells **(**Fig. 6E–I). Collectively, the findings suggest that PAK1 can function as an oncogene, whereby it serves as a target gene of circPTPN22/miR-6788-5p to promote autophagy, proliferation, migration, and invasion in GC.

### The circPTPN22/miR-6788-5p/PAK1 axis regulates autophagy in GC cells by activating Akt and Erk phosphorylation

P21-activated kinase 1 (PAK1) is the most widely studied and frequently detected member of the P21-activated kinases and is known to be dysregulated in various tumors [21, 22]. Clinical research has demonstrated that PAK1 not only participates in the tumorigenicity of diverse cancers [23] but also modulates autophagy levels through the Akt/mTOR or Erk/mTOR signaling pathway [24, 25]. To investigate whether the circPTPN22/miR-6788-5p/PAK1 axis could trigger the phosphorylation of Akt and Erk proteins, we conducted Western blot assays. We observed that PAK1 expression decreased upon circPTPN22 knockdown, and the phosphorylation of Akt and Erk was inhibited, which could be rescued by adding a miR-6788-5p inhibitor or PAK1 overexpression vector. Conversely, overexpression of circPTPN22 led to increased PAK1 expression and phosphorylation levels of Akt and Erk, which could be alleviated by the addition of miR-6788-5p mimic or PAK1 interference vector (Fig. 6J). This bundle of results suggests that circPTPN22 can harmoniously regulate the phosphorylation of Akt and Erk mediated by the miR-6788-5p/PAK1 axis, thereby affecting GC autophagy.

### circPTPN22 promotes tumor progression in BALB/c nude mice

**Fig. 7:**
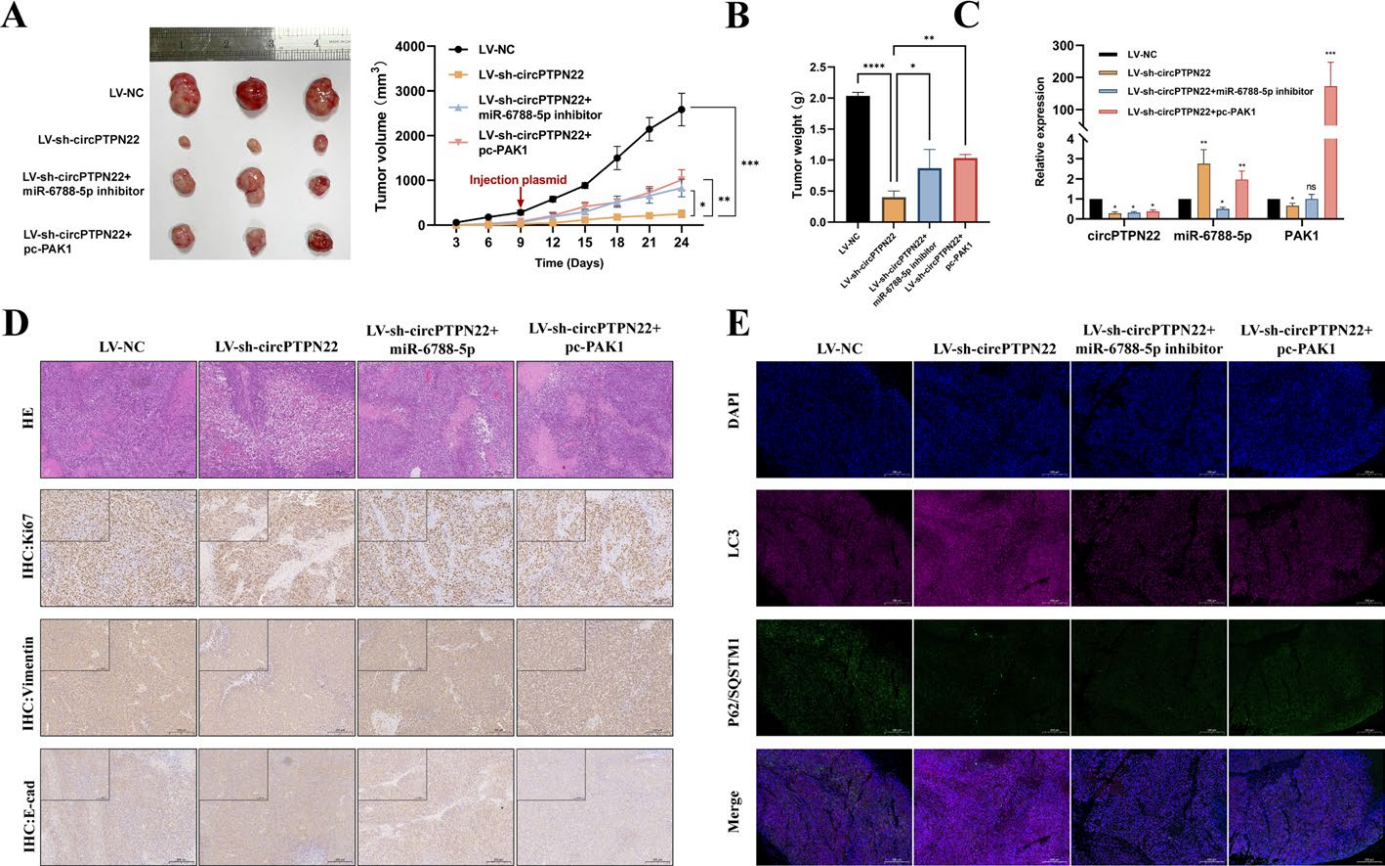
Knockdown of circPTPN22 can inhibit the progression of GC via autophagy in BALB/c nude mice. **A** Tumors formed by different treatment groups of BALB/c nude mice and tumor growth curves. **B** Tumor weights of each group at the end of the experiment. **C** mRNA expression levels of circPTPN22, miR-6788-5p, and PAK1 in different groups validated by qRT-PCR. **D** Hematoxylin and eosin staining showing the structure of xenograft tumors, scale bar: 200 μM. Immunohistochemistry demonstrating protein expression levels of Ki67, Vimentin, and E-cad in different groups, scale bar: 100 μM and 200 μM. **E** Immunofluorescence showing protein expression levels of LC3 and P62 in different groups, scale bar: 200 μM. *p < 0.05, **p < 0.01, ***p < 0.001, ****p < 0.001

In order to further investigate the role of circPTPN22 in vivo, we treated AGS cells differently and established a xenograft tumor model in BALB/c nude mice. The results showed that compared to the LV-NC group, knocking down circPTPN22 resulted in a decrease in tumor weight and volume, slower growth rate, and reduced expression of PAK1 in the tumor (Fig. 7A–C). IHC results revealed that knocking down circPTPN22 led to a decrease in the protein levels of Ki67 and vimentin, but an increase in E-cadherin protein level (Fig. 7D). This suggests that knocking down circPTPN22 weakens overall tumor progression. Furthermore, IF results showed that knocking down circPTPN22 resulted in an increase in the expression level of LC3 and a decrease in P62 protein level, indicating that knocking down circPTPN22 can promote autophagy within the tumor (Fig. 7E). Surprisingly, all these effects could be reversed by adding miR-6788-5p inhibitor or PAK1 overexpression vector. These results indicate that knocking down circPTPN22 can enhance autophagy and inhibit tumorigenicity of GC cells through the miR-6788-5p/PAK1 axis.

### The transcription factor RUNX1 can negatively regulate the expression of circPTPN22.

**Fig. 8:**
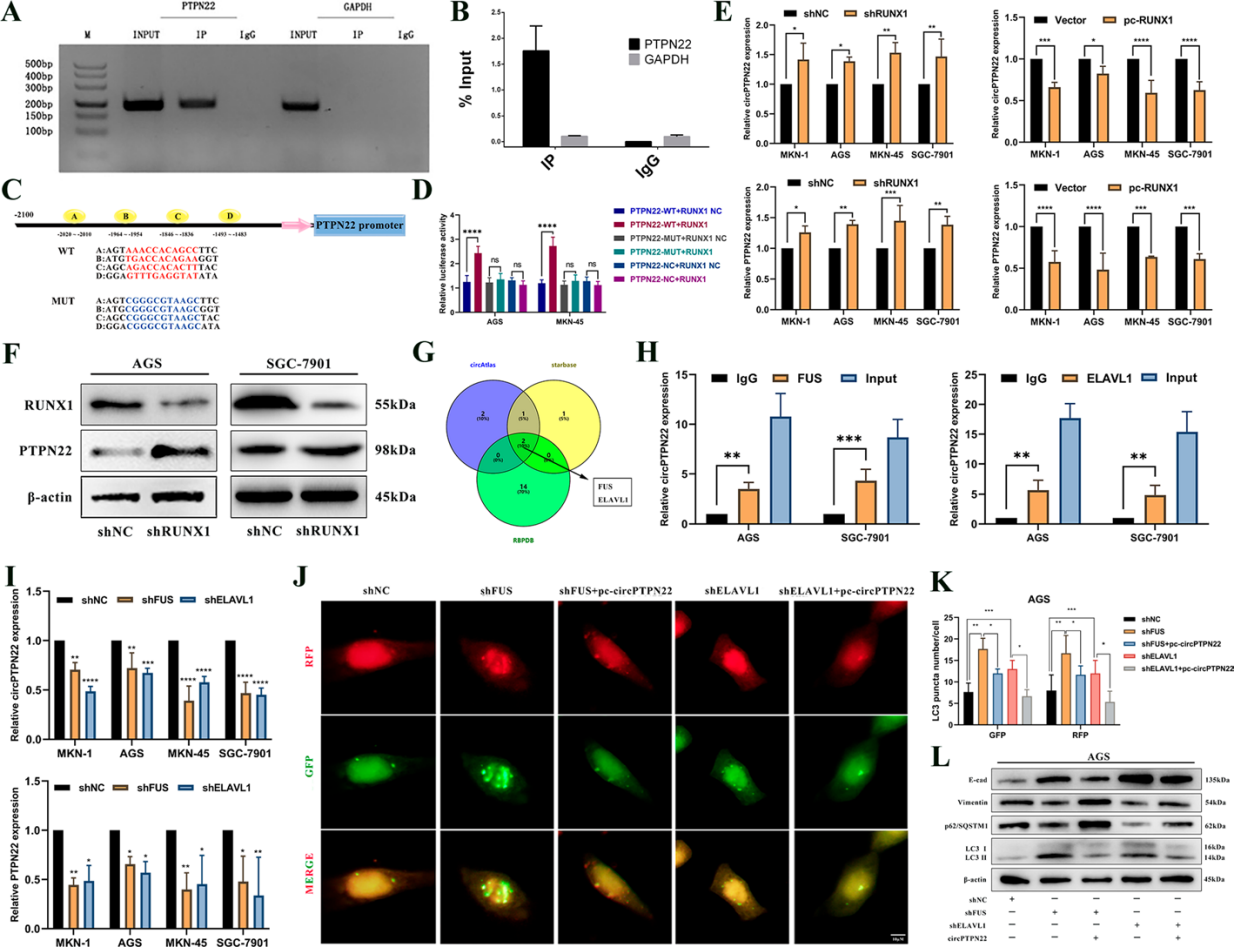
RUNX1, FUS and ELAVL1 can regulate the expression of circPTPN22. **A **and** B** ChIP-qPCR detection of GC cells ChIP-qPCR detection of PTPN22 promoter in GC cells may bind to the RUNX1 binding site, and IgG was used as a negative control. **C** Schematic diagram showing WT and Mut sequences of RUNX1 four putative binding sites on the PTPN22 promoter. **D** The combination of RUNX1 and PTPN22 was verified by dual luciferase reporter assay. **E** qRT-PCR assay to detect the expression of circPTPN22 and PTPN22 in GC cells after knockdown or overexpression of RUNX1. **F** Western blot detection of RUNX1 and PTPN22 protein expression in GC cells after RUNX1 knockdown. **G** Bioinformatic analysis of RNA-binding proteins associated with pre-circPTPN22. **H** RIP-qPCR detection of circPTPN22 expression in GC cells. **I** Expression of circPTPN22 and PTPN22 in GC cells after knockdown of FUS or ELAVL1. **J **and** K** After knocking down FUS or ELAVL1 in mRFP-GFP-LC3-labeled GC cells, the overexpression plasmid of circPTPN22 was added, and the autophagic flux was observed by confocal microscopy. Scale bar, 10 μM. **L** After knocking down FUS or ELAVL1, the overexpression plasmid of circPTPN22 was added, and the protein levels of E-cad, vimentin, P62/SQSTM1 and LC3 B in GC cells were detected by Western blot. *p < 0.05, **p < 0.01, ***p < 0.001, ****p < 0.001

Regulating circRNAs has attracted considerable attention. We sought to investigate the factors that control the expression of circPTPN22. Using bioinformatics methods, we predicted that the transcription factor, RUNX1, could bind to the PTPN22 promoter region of the circPTPN22 transcription gene, as there are seven binding sites (Table S4). The expression of RUNX1 is downregulated in GC tissues (Figure S5A). ChIP experiments were subsequently conducted, and it was confirmed that RUNX1 could bind tightly to the PTPN22 promoter region, thereby inhibiting its transcriptional activity (Fig. 8A and B). Four sequences with a binding degree score > 8.0 in the predicted sites were integrated into a dual fluorescent expression vector for PTPN22, and dual-luciferase reporter gene assays were used to confirm that PTPN22 can directly bind to RUNX1 (Fig. 8C and D). Thereafter, we transfected RUNX1 knockdown and overexpression vectors and found that the expression of PTPN22 and circPTPN22 increased after RUNX1 knockdown and decreased after overexpression (Fig. 8E). Additionally, Western blot assays revealed that PTPN22 expression increased after RUNX1 knockdown (Fig. 8F). These results demonstrate that the transcription factor RUNX1 can restrict the transcription of PTPN22 and negatively regulate the expression of circPTPN22.

### Alternative splicing of circPTPN22 generation by FUS and ELAVL1

Studies have shown that circRNAs are regulated by alternative splicing of the respective pre-mRNA, which is primarily triggered by RBPs [26]. We conducted bioinformatics cross-analysis to identify the putative RBPs, FUS, and ELAVL1 proteins that bind to pre-PTPN22 (Fig. 8G). Both RBPs are upregulated in GC (Figure S5B). The RIP experiment revealed that the enrichment of circPTPN22 in the experimental (FUS and ELAVL1) groups was significantly higher than that in the IgG group (Fig. 8H), indicating that pre-PTPN22 binds to FUS and ELAVL1. Furthermore, circPTPN22 and PTPN22 expressions were markedly reduced upon FUS and ELAVL1 knockdown, suggesting that FUS and ELAVL1 can positively regulate circPTPN22 expression (Fig. 8I). Subsequent rescue experiments showed that the number of autophagosomes in GC cells gradually increased and transformed into autolytic lysosomes after FUS and ELAVL1 knockdown, with concurrent reductions in P62 expression and increased LC3-II expression. This phenomenon could be rescued by sh-circPTPN22 (Fig. 8J–L). Furthermore, CCK-8, cell colony formation, and Transwell rescue experiments uniformly showed that FUS and ELAVL1 knockdown inhibited GC cell proliferation, migration, and invasion, which could be rescued by LV-sh-circPTPN22 (Figure S5C-J). Overall, these findings indicate that FUS and ELAVL1 can positively regulate the alternative splicing of circPTPN22 and influence biological functions such as autophagy in GC cells.

### Autophagy accelerates the progression of GC by forming a feedback loop through FUS-regulated generation of circPTPN22

**Fig. 9:**
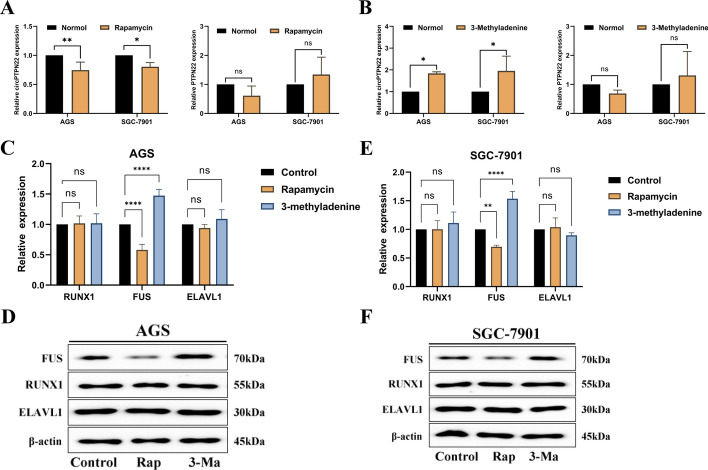
Autophagy Negatively Regulates the Expression of circPTPN22 by Modulating FUS. **A** qRT-PCR assay to detect the expression levels of circPTPN22 and PTPN22 after adding the autophagy activator Rap and the autophagy inhibitor 3-Ma. **B **and** C** Gene and protein expression of RUNX1, FUS, and ELAVL1 in AGS and SGC-7901 cells after the addition of Rap. **D **and** E** Gene and protein expression of RUNX1, FUS, and ELAVL1 in AGS and SGC-7901 cells after the addition of the autophagy inhibitor 3-Ma. *p < 0.05, **p < 0.01, ****p < 0.001

Studies have revealed that autophagy can induce the degradation of circRNAs [27, 28]. Consequently, to investigate the impact of autophagy on the expression of circPTPN22, AGS and SGC-7901 cells were treated with Rap and 3-MA. The results demonstrated a significant decrease in circPTPN22 expression, while PTPN22 remained unchanged upon the administration of Rap (Fig. 9A and B). In contrast, treatment with 3-MA resulted in a marked increase in circPTPN22 expression, with no effect on PTPN22 (Fig. 9A and B). These findings suggest that modifications in autophagy adversely regulate the expression of circPTPN22, without affecting its transcriptional gene, PTPN22. A growing body of research indicates that the degradation of circRNAs necessitates the involvement of critical transcription factors or functional proteins [29–33]. Therefore, we investigated the relationship between autophagy and the transcription factor RUNX1, as well as the RNA-binding proteins FUS and ELAVL1. The study revealed that the exposure of AGS and SGC-7901 cells to Rap did not affect the genes or proteins of RUNX1 and ELAVL1; however, the gene and protein levels of FUS were reduced (Fig. 9C and D). Similarly, exposure of AGS and SGC-7901 cells to 3-Ma did not impact the genes or proteins of RUNX1 and ELAVL1, whereas both the gene and protein levels of FUS were elevated (Fig. 9E and F). These findings suggest that alterations in autophagy regulate the degradation of the RNA-binding protein FUS gene or protein, thereby influencing the generation of circPTPN22.

## Discussion

Due to their unique molecular structure, circRNAs are capable of regulating various types of cancer, making them superior cancer diagnostic markers and therapeutic targets compared to linear RNAs [11]. Currently, an increasing number of studies have revealed the detailed mechanisms of certain circRNAs in several cancers, including GC. For instance, Shen et al. [34] demonstrated that circPDIA4 exerts oncogenic functions via different mechanisms in the cytoplasm and nucleus. In the cytoplasm, circPDIA4 binds to Erk1/2, thereby preventing its dephosphorylation leading to activation of the mitogen-activated protein kinase (MAPK) pathway. Meanwhile, in the nucleus, circPDIA4 interacts with DEAH box helicase 9 to increase the expression of various oncogenic circRNAs and promote GC progression. Liu et al. [35] discovered that reduced expression of circIPO7 directly binds to caprin-1, which is involved in mRNA translation, in GC. This interaction blocks caprin-1-G3BP1 interaction and sequesters caprin-1 and its target mRNAs, such as epidermal growth factor receptor and mTOR, from ribosomes. Consequently, the phosphoinositide 3-kinase/Akt/mTOR pathway is inactivated, making circIPO7 a potential target for cancer therapy. These findings indicate that circRNAs possess significant scientific and clinical value in the development and occurrence of GC. However, since GC is heterogeneous and complex at the molecular level, more research is needed to update the molecular regulatory relationships in GC involving circRNAs. In this study, we explored the specific mechanism of circPTPN22, a previously reported biomarker in GC, and discovered that its upregulated expression promotes GC cell proliferation, migration, and invasion. Moreover, activation of autophagy appears to be necessary for this effect, although inhibition of autophagy can rescue it. Autophagy is an evolutionarily conserved and dynamic process by which intracellular components are degraded through lysosomes [36]. circRNAs play a crucial role in gene regulation processes [37, 38]. For instance, in prostate cancer, heterogeneous nuclear ribonucleoprotein L-catalyzed upregulation of circCSPP1 leads to the induction of autophagy via the circCSPP1/miR-520 h axis, thereby regulating prostate tumor progression [39]. In GC, circCPM serves as a key regulator of both autophagy and fluorouracil (5-FU) resistance by targeting PRKAA2. These findings provide a theoretical foundation for evaluating GC efficacy and reversing 5-FU chemoresistance [40]. Therefore, it is reasonable to consider circPTPN22 as a novel biomarker and target for autophagy detection and treatment in GC.

Currently, exploration of the mechanism of circRNAs is mainly focused on three aspects: “miRNA sponge,” “RBP scaffolding,” and “serving as a simple polypeptide translation template.” Of these, acting as miRNA sponges in cancer cells is considered the most prevalent mechanism. It is well-established that in many cancers, including GC, miRNAs act as oncogenes or tumor suppressors, playing a pivotal role in tumor progression [41, 42]. As new members of the competitive endogenous RNA family and regulators of miRNA activity, circRNAs may exert a significant role in gene expression regulation in cancer and other diseases [43, 44]. Results from our study demonstrate that circPTPN22 in the cytoplasm directly binds to miR-6788-5p. Furthermore, our study reveals, for the first time, that miR-6788-5p serves as a tumor suppressor molecule in GC cells by promoting autophagy and inhibiting cell proliferation, migration, and invasion. Subsequently, our bioinformatics analysis identified PAK1 as a potential target related to autophagy. Through experiments involving dual-luciferase reporter genes, we found that *PAK1* is a direct downstream target gene of miR-6788-5p. Earlier studies suggest that PAK1 not only participates in the biological progression of various tumors but also regulates the autophagy level of tumors by promoting the phosphorylation of Akt or Erk [22]. In an earlier study, Dou et al. [24] found that PAK1 expression was positively correlated with Akt phosphorylation levels. Inhibiting PAK1 expression could block the Akt/mTOR signaling pathway, leading to autophagy induction induced by ivermectin. The Akt/mTOR pathway is the main pathway for autophagy regulation [25]. In addition to the above pathways, PAK1 can efficiently phosphorylate mitogen-activated protein kinase kinase (MEK1) at S298 in vivo and in vitro, leading to Erk1/2 phosphorylation and MAPK signaling pathway activation [45]. The MEK1/Erk1/2 pathway can also inhibit autophagy in GC cells. These findings suggest that PAK1 can jointly inhibit autophagy through two signaling pathways. Our study confirmed the role of this axis. We found that by transfecting the circPTPN22 interference plasmid, phosphorylation of Akt and Erk levels was inhibited. However, this effect can be restored effectively by co-transfection with a miR-6788-5p inhibitor or PAK1 overexpression plasmid. In summary, our study indicates that circPTPN22 serves as a miR-6788-5p sponge to regulate PAK1 expression, which can activate Akt or Erk phosphorylation via the miR-6788-5p/PAK1 axis to modulate autophagy and other biological functions in GC.

In the realm of non-coding research, the regulation of circRNAs generation has persistently been a pivotal issue. Prior studies have demonstrated that factors influencing the generation of circRNAs include cis-acting elements, trans-acting factors, RBPs, splicing factors, and transcription factors, among others [46–48]. Transcription factors and RBPs are two primary regulatory agents that impact the generation and function of circRNAs through diverse mechanisms. For instance, in HPV-positive cervical cancer cells, transcription factor FOXA1 activates the expression of circODC1, which in turn promotes cancer cell growth by competitively binding to miR-607, thereby relieving the inhibition on ODC1 [49]. Additionally, circSEPT9 mediated by E2F1 and EIF4A3 propels the carcinogenesis and progression of triple-negative breast cancer via the circSEPT9/miR-637/LIF axis [50]. RUNX1, identified as a critical transcription factor, has been observed to regulate circRNAs expression, consequently influencing tumor progression. For example, RUNX1 modulates the expression of circ_0014614, thereby affecting the differentiation of myeloid cells into megakaryocytes [51]. In GC, RUNX1 has been found to suppress the transcription of PTPN22, thus inhibiting the expression of circPTPN22. Similarly, at the level of RBPs, two significant regulators are the FUS protein and ELAVL1 protein, both of which are among the most prevalent RNA-binding proteins. The FUS protein is involved in regulating intracellular RNA transport, mRNA synthesis, alternative splicing, and polyadenylation site selection [52]. He et al. [53] demonstrated that FUS binds to circ_002136, promoting the formation of circ_002136 in gliomas. ELAVL1, a ubiquitously expressed neuron-like ELAV protein, participates in various biological behaviors of cancer [54]. Most precursor mRNAs of circRNAs possess binding sites for ELAVL1, providing a potential pathway for ELAVL1-mediated regulation of circRNAs generation. In this study, we discovered that ELAVL1 and FUS positively regulate the expression of circPTPN22 via their interaction with pre-PTPN22. Thus, transcription factors and RBPs collectively participate in regulating the generation of circPTPN22.

Moreover, we found that autophagy also impacts the expression of circPTPN22. Autophagy can induce the degradation of circRNAs, primarily through the degradation of their mRNAs or the engulfment of deposited RBPs. This study revealed that changes in autophagy specifically affect the expression of circPTPN22, while the expression of its parent gene, PTPN22, remains unaffected. We speculate that changes in autophagy state modify RBPs without impacting the transcription level. Specifically, we observed that increased autophagy reduces the expression of the FUS gene or protein, thereby affecting the expression of circPTPN22. Finally, we identified an intriguing feedback loop: upregulated circPTPN22 in GC inhibits cellular autophagy via the miR-6788-5p/PAK axis, but suppression of autophagy increases the expression of FUS, further enhancing circPTPN22 expression. While this discovery supplements the feedback mechanism between circRNAs and autophagy, our exploration of this feedback loop remains insufficient, necessitating further research to illuminate the key factors operating within the autophagy pathway.

## Conclusions

**Fig. 10:**
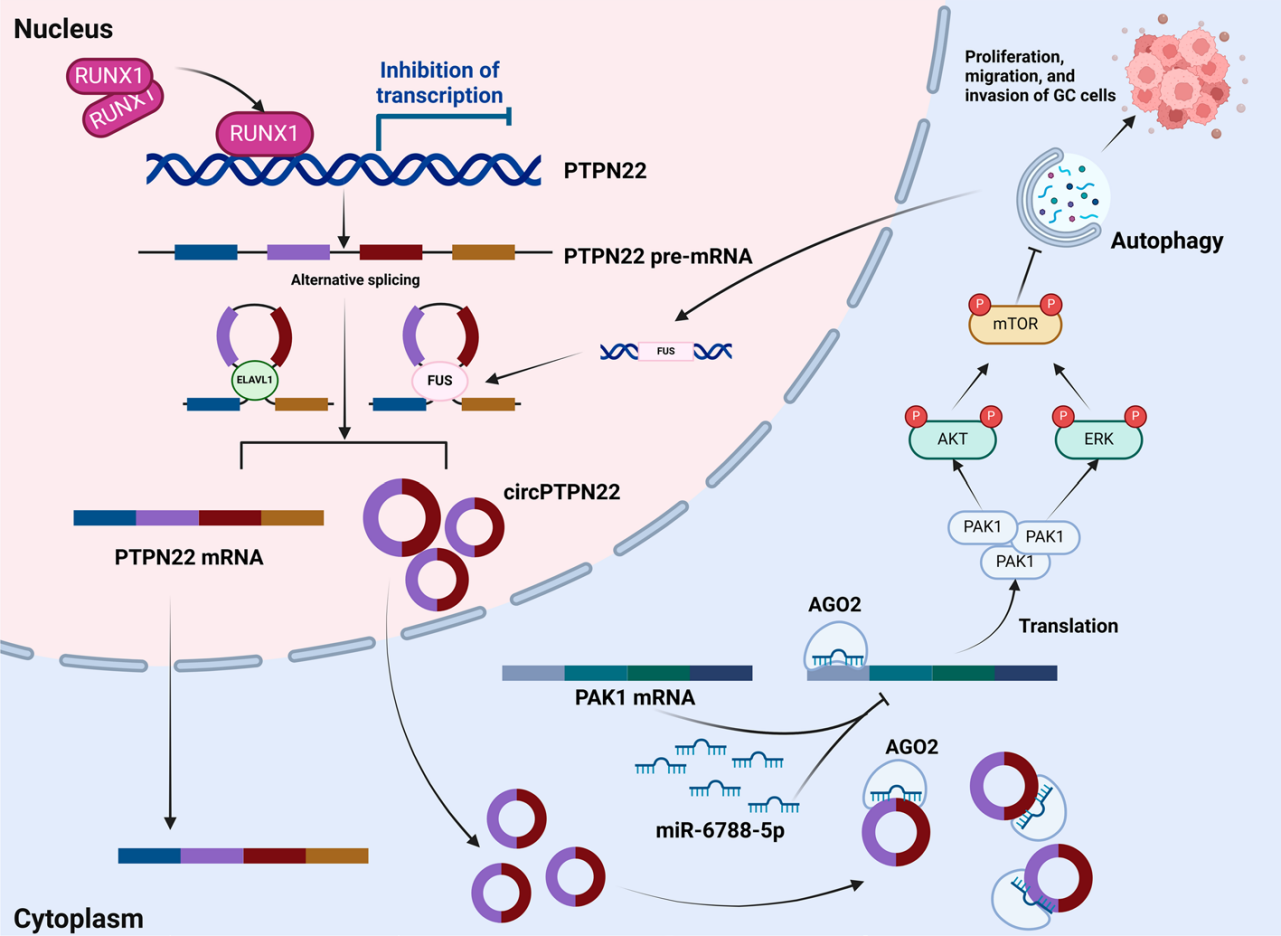
In GC, circPTPN22 is negatively regulated by the transcription factor RUNX1 and positively regulated by the RNA-binding proteins FUS and ELAVL1. Through the miR-6788-5p/PAK1 axis, circPTPN22 activates the phosphorylation of Akt and Erk and inhibits autophagy. Moreover, the inhibition of autophagy creates a positive feedback loop that increases FUS expression, which in turn further enhances circPTPN22 expression. This ultimately exacerbates the progression of GC

In summary, our research indicates that circPTPN22, which is upregulated in GC and negatively regulated by the transcription factor RUNX1 and positively regulated by RNA-binding proteins FUS and ELAVL1, can activate Akt and Erk phosphorylation via the miR-6788-5p/*PAK1* axis and inhibit autophagy. Furthermore, the inhibition of autophagy can create a feedback loop that increases FUS expression, thereby further enhancing circPTPN22 expression. This feedback loop between circPTPN22 and autophagy ultimately exacerbates the progression of GC. The schematic diagram is shown in Fig. 10. Overall, these findings offer new insights into the role of circPTPN22 in GC progression and autophagy regulation, underscore the regulatory role of autophagy in GC progression, and demonstrate circPTPN22 as a promising diagnostic biomarker and therapeutic target for GC.

### Supplementary Information

**Additional file 1:** Table S1: The primer sequence of qRT-PCR. Table S2: Plasmid sequences were transfected. Table S3 Bioinformatics analysis was used to analyze the specific information. Table S4 Seven sites where RUNX1 binds to the promoter region of PTPN22. Figure S1. circPTPN22 can promote the proliferation, migration, and invasion of GC cells. A and B. qRT-PCR detection of circPTPN22 knockdown and overexpression efficiency in GC cells. C-H. The effect of knockdown or overexpression of circPTPN22 on the proliferation of GC cells was detected by cck-8 assay (C and D) and EdU assay (E–H). I-P. The effects of knockdown or overexpression of circPTPN22 on migration (I-L) and invasion (M-P) of GC cells were detected by transwell assay. **p < 0.01, ***p < 0.001, ****p < 0.001. Figure S2. Evidence for circPTPN22 binding to miR-6788-5p. A and B. Bioinformatics analysis of RBPs, IRES sites and ORF reading frames that circPTPN22 may bind. C. Expression of miR-6788-5p in GC cells. D. Survival analysis of GC patients in miR-6788-5p high and low groups. *p < 0.05, **p < 0.01, ****p < 0.001. Figure S3. miR-6788-5p inhibited the proliferation, migration, and invasion of GC cells. A-E. Using cck-8 (A), cell colony formation assay (B and C), and EdU cell proliferation assay (D and E) to detect the effect of adding miR-6788-5p inhibitor or mimic on the proliferation of GC cells. F and G. Transwell assay was used to detect the effect of adding miR-6788-5p inhibitor or mimic on the migration and invasion of GC cells. H. Western blot detection of E-cad and vimentin protein levels in GC cells after adding miR-6788-5p inhibitor or mimic. **p < 0.01, ***p < 0.001, ****p < 0.001. Figure S4. The expression level of PAK1 in GC cells. *p < 0.05, ****p < 0.001. Figure S5. FUS and ELAVL1 can partially restore the effects of circPTPN22 on the proliferation, migration, and invasion of GC cells. A. The expression level of RUNX1 in gastric cancer tissues. B. The expression level of FUS and ELAVL1 in GC tissues. C. Knockdown efficiency of FUS and ELAVL1 in GC cells. D-F. After the knockdown of FUS or ELAVL1, the overexpression plasmid of circPTPN22 was transfected, and the proliferation of GC cells was detected by cck-8 (D) and cell colony formation assay (E and F). G-J. After the knockdown of FUS or ELAVL1, the overexpression plasmid of circPTPN22 was transfected, and the migration (G and H) and invasion (I and J) abilities of GC cells were detected by transwell assay. *p < 0.05, **p < 0.01, ***p < 0.001, ****p < 0.001. Supplementary file- the full uncropped Gels and Blots image

## Data Availability

The datasets used and/or analyzed during the current study are available from the corresponding author on reasonable request.

## Abbreviations

circRNAs: Circular RNAs
GC: Gastric cancer
PAK1: P21-activated kinase-1
PRKA: Activating a kinase
Erk: Extracellular signal-regulated kinase
RUNX1: Runt-related transcription factor 1
FUS: Fused in sarcoma
ELAVL1: ELAV-like protein 1
miRNA: MicroRNA
RBPs: RNA-binding proteins
GES-1: Gastric epithelial cells
shRNA: Short hairpin RNA
CCK-8: Cell counting kit 8
PBS: Phosphate-buffered saline
EdU: 5-Ethynyl-2′-deoxyuridine
TEM: Transmission electron microscopy
FISH: Fluorescence in situ hybridization
DAPI: 4′,6-Diamidino-2-phenylindole
IF: Immunofluorescence
IHC: Immunohistochemistry
ORF: Open reading frame
IRES: Internal ribosome entry site
RIP: RNA immunoprecipitation
ChIP: Chromatin IP
Rap: Rapamycin
3-MA: 3-Methyladenine
IgG: Negative control
5-FU: Fluorouracil
MEK1: Mitogen-activated protein kinase kinase
