# Supplementary material for: RUNX1, FUS, and ELAVL1-induced circPTPN22 promote gastric cancer cell proliferation, migration, and invasion through miR-6788-5p/PAK1 axis-mediated autophagy
Source: Cell Mol Biol Lett. 2024 Jul 2;29:95. doi: 10.1186/s11658-024-00610-9 (PMC11218243; doi:10.1186/s11658-024-00610-9)

**RUNX1, FUS, and ELAVL1-induced circPTPN22 promote gastric cancer cell proliferation, migration, and invasion through miR-6788-5p/PAK1 axis-mediated autophagy**

**Running title:** circPTPN22 promotes gastric cancer progression through autophagy.

**Shuo Ma^1, 2, 3, #^, Yanhua Xu^1, 4, #^, Xinyue Qin^1^, Mei Tao^1^, Xinliang Gu^1^, Lei Shen^1^, Yinhao Chen^5^, Ming Zheng^1^, Shiyi Qin^1^, Guoqiu Wu^2, 3, *^and Shaoqing Ju^1, *^**

^1^ Department of Laboratory Medicine, Affiliated Hospital of Nantong University, Medical School of Nantong University, Nantong 226001, Jiangsu, China.

^2^ Center of Clinical Laboratory Medicine, Zhongda Hospital, Medical School of Southeast University, Nanjing 210009, Jiangsu, China.

^3^ Medical School of Southeast University, Nanjing 210009, Jiangsu, China.

^4^ Department of Laboratory Medicine, Northern Jiangsu People’s Hospital Affiliated to Yangzhou University, Yangzhou 225000, Jiangsu, China.

^5^ Department of Integrated Oncology, Center for Integrated Oncology (CIO), University Hospital Bonn, Bonn, Germany.

**^#^Shuo Ma and Yanhua Xu contributed equally to this article.**

**Correspondence:**

**Shaoqing Ju,** Department of Laboratory Medicine, Affiliated Hospital of Nantong University, Xisi Road, NO.20, Nantong 226001, Jiangsu, China; E-mail: jsq814@hotmail.com.

**Guoqiu Wu,** Center of Clinical Laboratory Medicine, Zhongda Hospital, Southeast University, Nanjing 210009, Jiangsu, China; Diagnostics Department, Medical School of Southeast University, Nanjing 210009, Jiangsu, China; Email: 101008404@seu.edu.cn.

**Supplementary documents**

| **Table S1: The primer sequence of qRT-PCR** | | |
| --- | --- | --- |
| **Name** | **Forward primers（5’-3’）** | **Reverse primer（5’-3’）** |
| circPTPN22 | TCACAAGGAGTAAGGAGAAT | CTCTTGCTTGGTCTAAGTATC |
| PTPN22 | AGGCAGACAAAACCTATCCTACA | TGGGTGGCAATATAAGCCTTG |
| PAK1 | CAGCCCCTCCGATGAGAAATA | CAAAACCGACATGAATTGTGTGT |
| PRKCA | GTCCACAAGAGGTGCCATGAA | AAGGTGGGGCTTCCGTAAGT |
| RUNX1 | CTGCCCATCGCTTTCAAGGT | GCCGAGTAGTTTTCATCATTGCC |
| FUS | ATGGCCTCAAACGATTATACCCA | GTAACTCTGCTGTCCGTAGGG |
| ELAVL1 | GGGTGACATCGGGAGAACG | CTGAACAGGCTTCGTAACTCAT |
| GADPH | TCCCATCACCATCTTCCAGG | GATGACCCTTTTGGCTCCC |
| U6 | AACGCTTCACGAATTTGCGT | CTCGCTTCGGCAGCACA |

| **Table S2: Plasmid sequences were transfected** | |
| --- | --- |
| **Name** | **Sequence（5’-3’）** |
| LV-sh-circPTPN22 | GAGUAAGGAGAAUUCUCACCA |
| shPAK1 | GCTGAGGATTACAATTCTTCT |
| shRUNX1 | GGATCCATTGCCTCTCCTTCT |
| shFUS | CAGAGCAGCTATTCTTCTTAT |
| shELAVL1 | GAACGAATTTGATCGTCAACT |
| shNC | TTCTCCGAACGTGTCACGT |
| miR-6788-5p inhibitor | UCUUCACCACUCUUCUCCCAG |
| inhibitor-NC | CAGUACUUUUGUGUAGUACAA |
| miR-6788-5p mimic | CUGGGAGAAGAGUGGUGAAGA |
| mimic-NC | UUCUCCGAACGUGUCACGUTT |

| **Table S3 Bioinformatics analysis was used to analyze the specific information** | |
| --- | --- |
| **Name** | **Web site** |
| Targetscan | http://www.targetscan.org/vert_71/ |
| circBank | http://www.circbank.cn/index.html |
| Circinteractome | https://circinteractome.nia.nih.gov/index.html |
| circRNADb | http://reprod.njmu.edu.cn/cgi-bin/circrnadb/circRNADb.php |
| miRWalk | http://mirwalk.umm.uni-heidelberg.de/ |
| miRPathDB | https://mpd.bioinf.uni-sb.de/ |
| miRDB | http://mirdb.org/index.html |
| Pathcards | https://pathcards.genecards.org/ |
| JASPAR | https://jaspar.genereg.net/ |
| circAtlas | http://circatlas.biols.ac.cn/ |
| RBPDB | http://rbpdb.ccbr.utoronto.ca/ |
| Starbase | https://starbase.sysu.edu.cn/ |

| **Table S4 Seven sites where RUNX1 binds to the promoter region of PTPN22** | | | | | | | | |
| --- | --- | --- | --- | --- | --- | --- | --- | --- |
| **Matrix ID** | **Name** | **Score** | **Relative score** | **Sequence ID** | **Start** | **End** | **Strand** | **Predicted sequence** |
| MA0002.1 | MA0002.1. RUNX1 | 10.910171 | 0.91202396 | NC_000001.11:c113873759-113871660 | 1954 | 1964 | - | TTCTGTGGTCA |
| MA0002.1 | MA0002.1. RUNX1 | 8.588789 | 0.85189663 | NC_000001.11:c113873759-113871660 | 1483 | 1493 | + | GTTTGAGGTAT |
| MA0002.1 | MA0002.1. RUNX1 | 8.569008 | 0.85138427 | NC_000001.11:c113873759-113871660 | 2010 | 2020 | - | GGCTGTGGTTT |
| MA0002.1 | MA0002.1. RUNX1 | 8.25088 | 0.84314428 | NC_000001.11:c113873759-113871660 | 1836 | 1846 | - | AAGTGTGGTCT |
| MA0002.1 | MA0002.1. RUNX1 | 7.4636927 | 0.82275492 | NC_000001.11:c113873759-113871660 | 176 | 186 | - | GAATATGGTAA |
| MA0002.1 | MA0002.1. RUNX1 | 7.417141 | 0.82154916 | NC_000001.11:c113873759-113871660 | 684 | 694 | - | TATCTTGGTTA |
| MA0002.1 | MA0002.1. RUNX1 | 7.3874874 | 0.82078109 | NC_000001.11:c113873759-113871660 | 1015 | 1025 | + | AAATGTGGAAA |


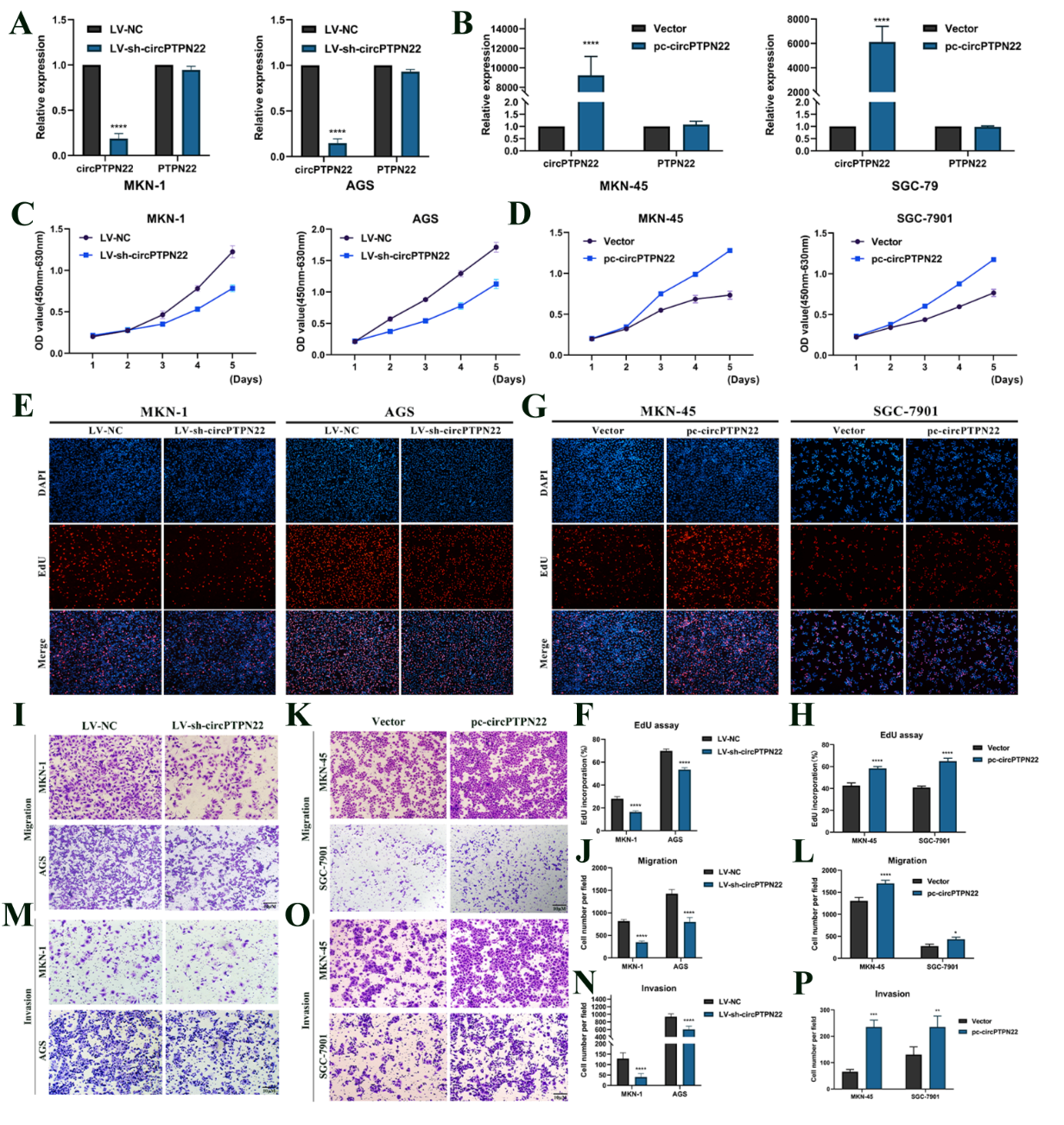


**Supplementary Figure 1.** **circPTPN22 can promote the proliferation, migration, and invasion of GC cells. A and B.** qRT-PCR detection of circPTPN22 knockdown and overexpression efficiency in GC cells. **C-H.** The effect of knockdown or overexpression of circPTPN22 on the proliferation of GC cells was detected by cck-8 assay **(C and D)** and EdU assay **(E-H)**. I-P. The effects of knockdown or overexpression of circPTPN22 on migration **(I-L)** and invasion **(M-P)** of GC cells were detected by transwell assay. **p < 0.01, ***p < 0.001, ****p < 0.001.


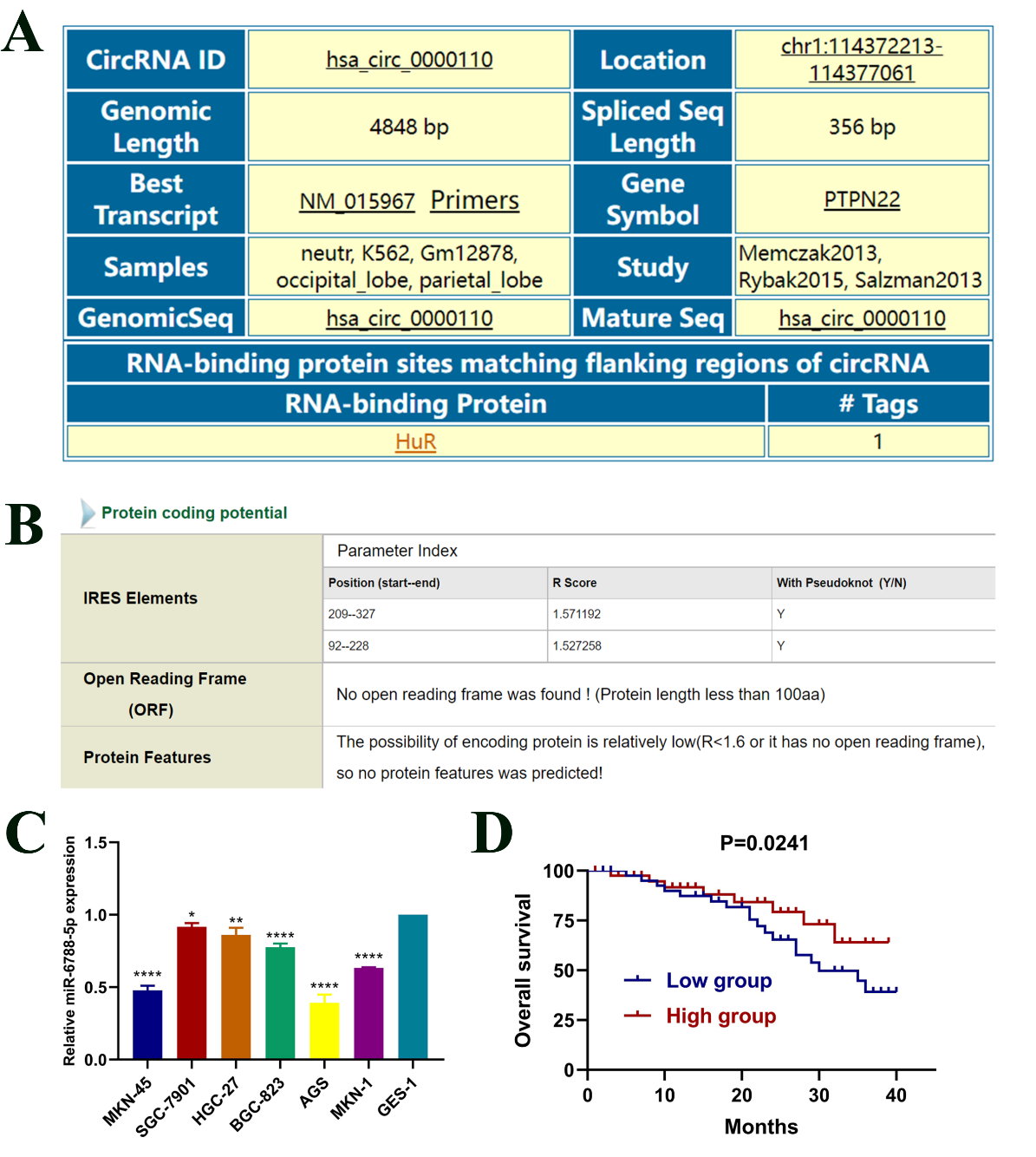


**Supplementary Figure 2.** **Evidence for circPTPN22 binding to miR-6788-5p. A and B.** Bioinformatics analysis of RBPs, IRES sites and ORF reading frames that circPTPN22 may bind. **C.** Expression of miR-6788-5p in GC cells. **D.** Survival analysis of GC patients in miR-6788-5p high and low groups. *p < 0.05, **p < 0.01, ****p < 0.001.


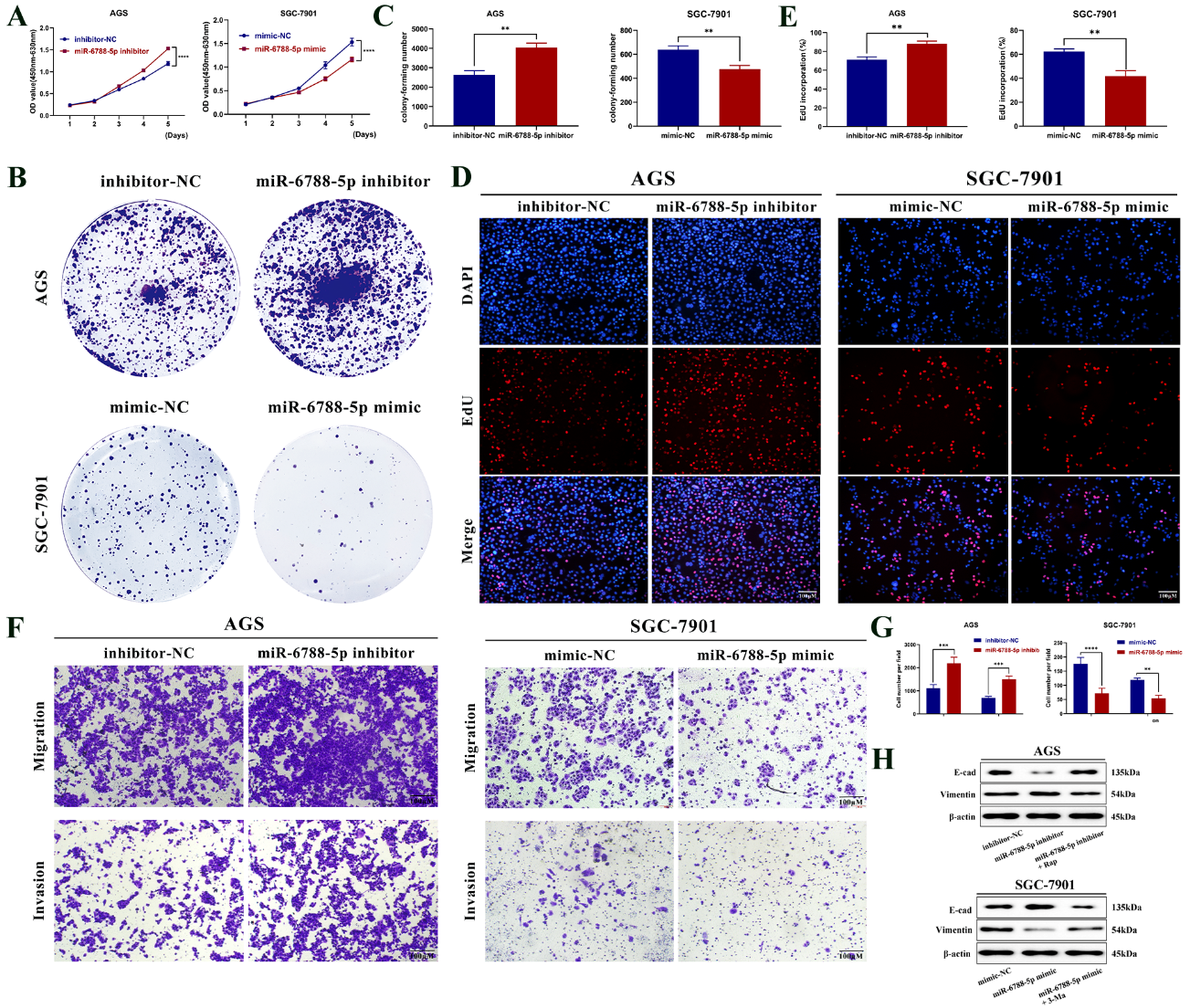


**Supplementary Figure 3. miR-6788-5p inhibited the proliferation, migration, and invasion of GC cells. A-E.** Using cck-8 (**A**), cell colony formation assay (**B and C**), and EdU cell proliferation assay (**D and E**) to detect the effect of adding miR-6788-5p inhibitor or mimic on the proliferation of GC cells. **F and G.** Transwell assay was used to detect the effect of adding miR-6788-5p inhibitor or mimic on the migration and invasion of GC cells. **H.** Western blot detection of E-cad and vimentin protein levels in GC cells after adding miR-6788-5p inhibitor or mimic. **p < 0.01, ***p < 0.001, ****p < 0.001.


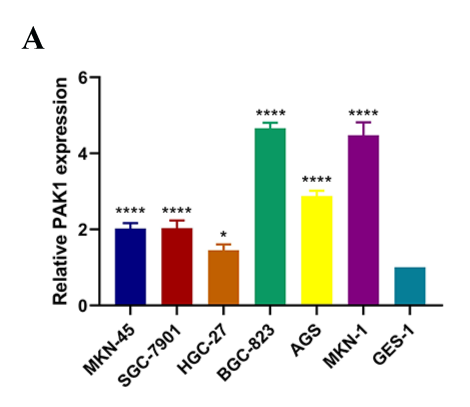


**Supplementary Figure 4. The expression level of PAK1 in GC cells.** ***p < 0.05, ****p < 0.001.**

**
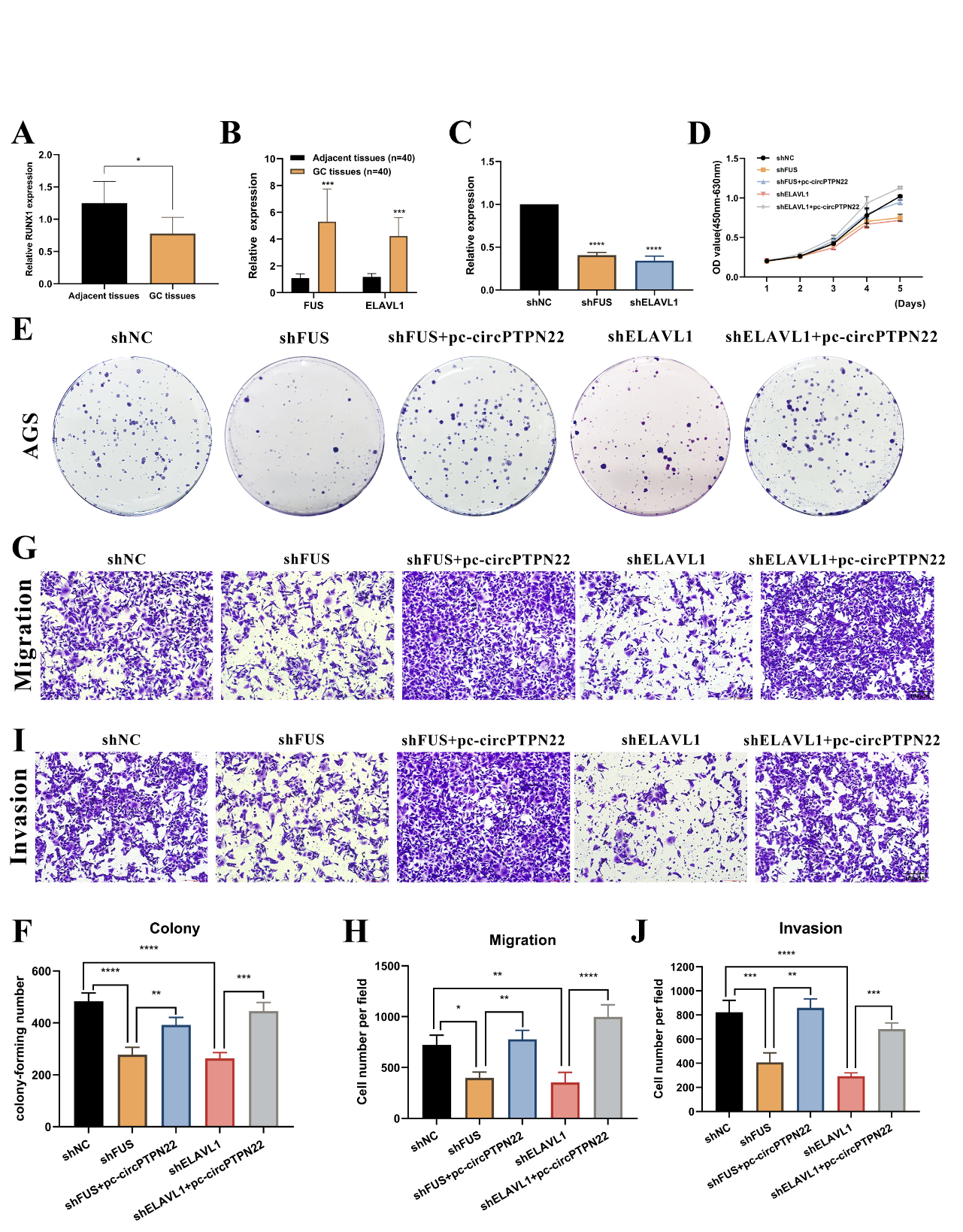
**

**Supplementary Figure 5. FUS and ELAVL1 can partially restore the effects of circPTPN22 on the proliferation, migration, and invasion of GC cells. A.** The expression level of RUNX1 in gastric cancer tissues. **B.** The expression level of FUS and ELAVL1 in GC tissues. **C.** Knockdown efficiency of FUS and ELAVL1 in GC cells. **D-F.** After the knockdown of FUS or ELAVL1, the overexpression plasmid of circPTPN22 was transfected, and the proliferation of GC cells was detected by cck-8 (**D**) and cell colony formation assay (**E and F**). **G-J.** After the knockdown of FUS or ELAVL1, the overexpression plasmid of circPTPN22 was transfected, and the migration (**G and H**) and invasion (**I and J**) abilities of GC cells were detected by transwell assay. *p < 0.05, **p < 0.01, ***p < 0.001, ****p < 0.001.

**Supplementary file-** **the full uncropped Gels and Blots image**

**FIGURE 1B-**

MKN1-P62 and AGS-P62


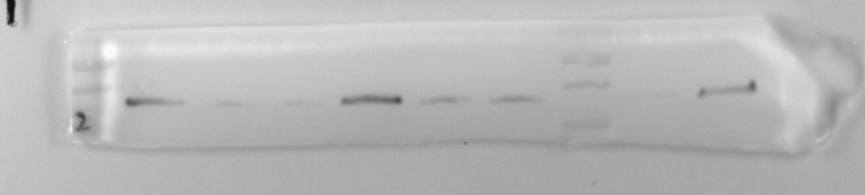


MKN1-LC3 and AGS-LC3


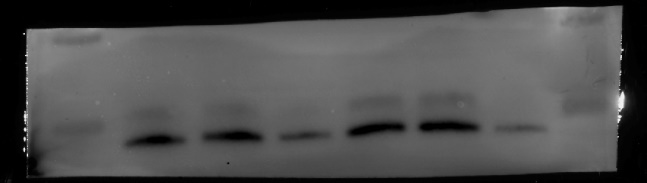


MKN1-Actin and AGS-Actin


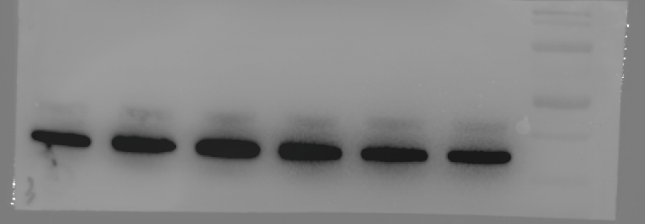


MKN45-P62 and SGC-7901-P62


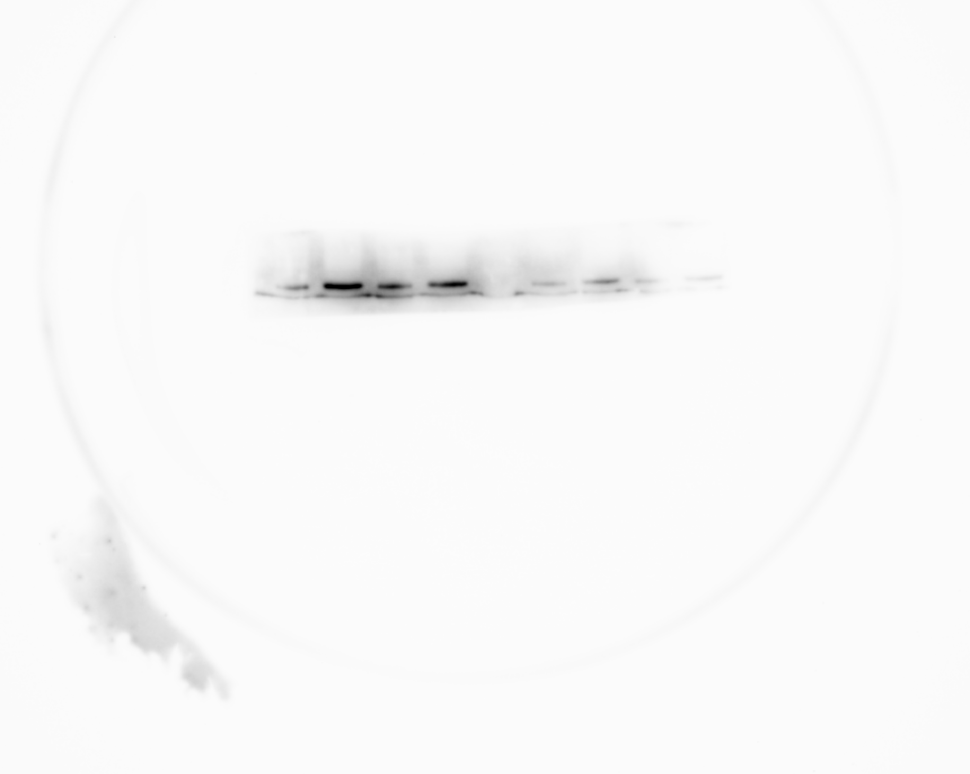


MKN45-LC3 and SGC-7901- LC3


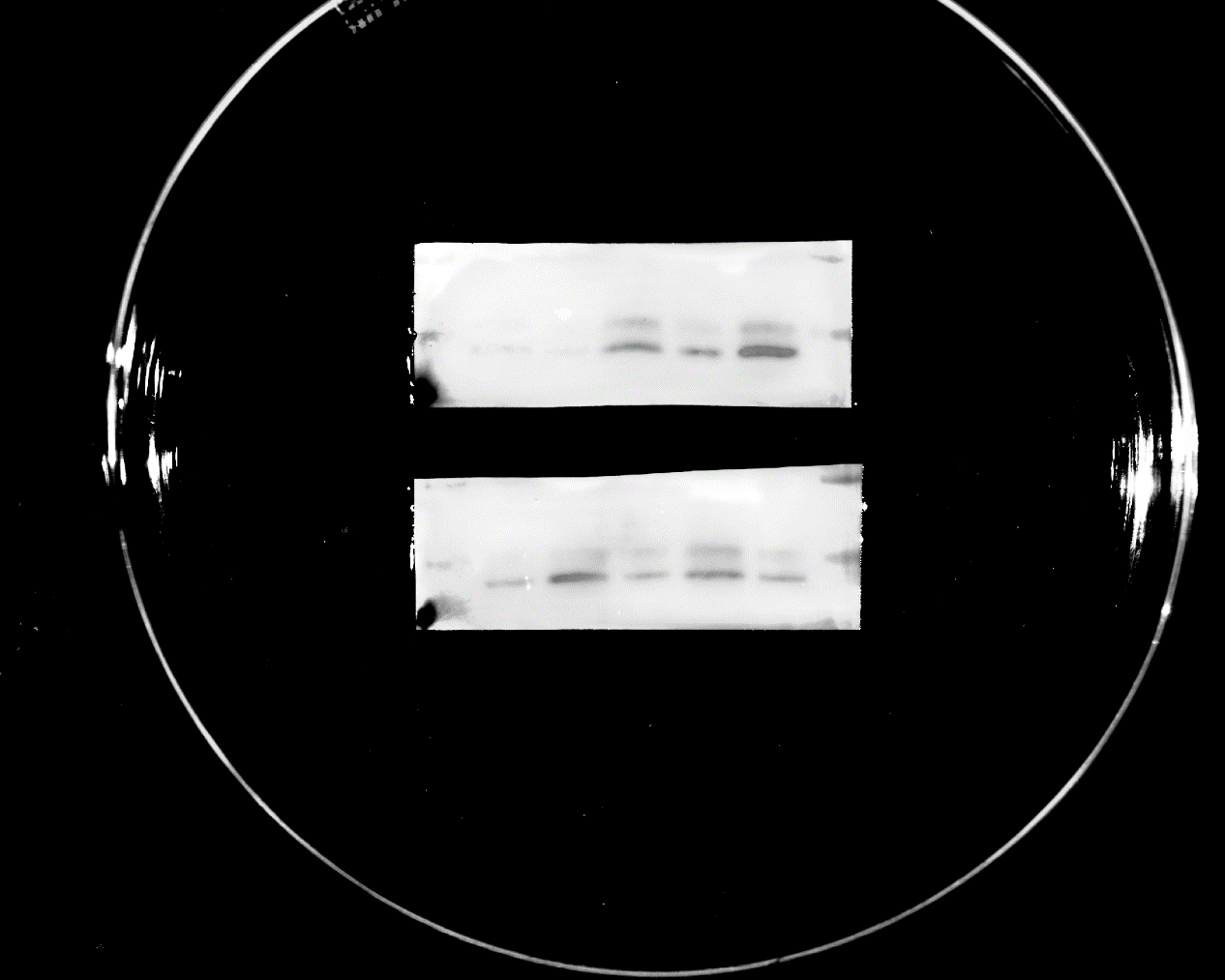


MKN45-Actin and SGC-7901- Actin


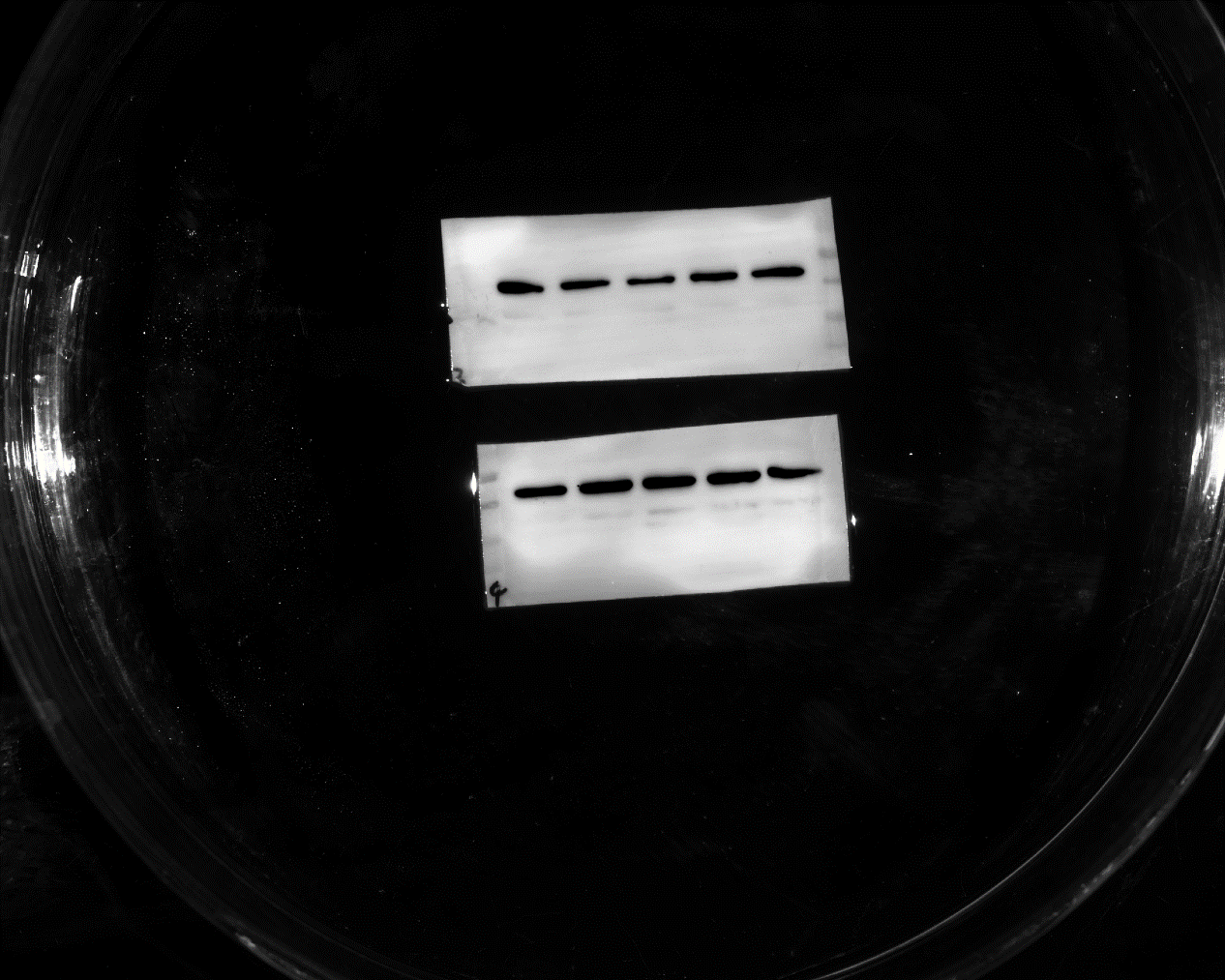


**FIGURE 4B**

AGS-P62


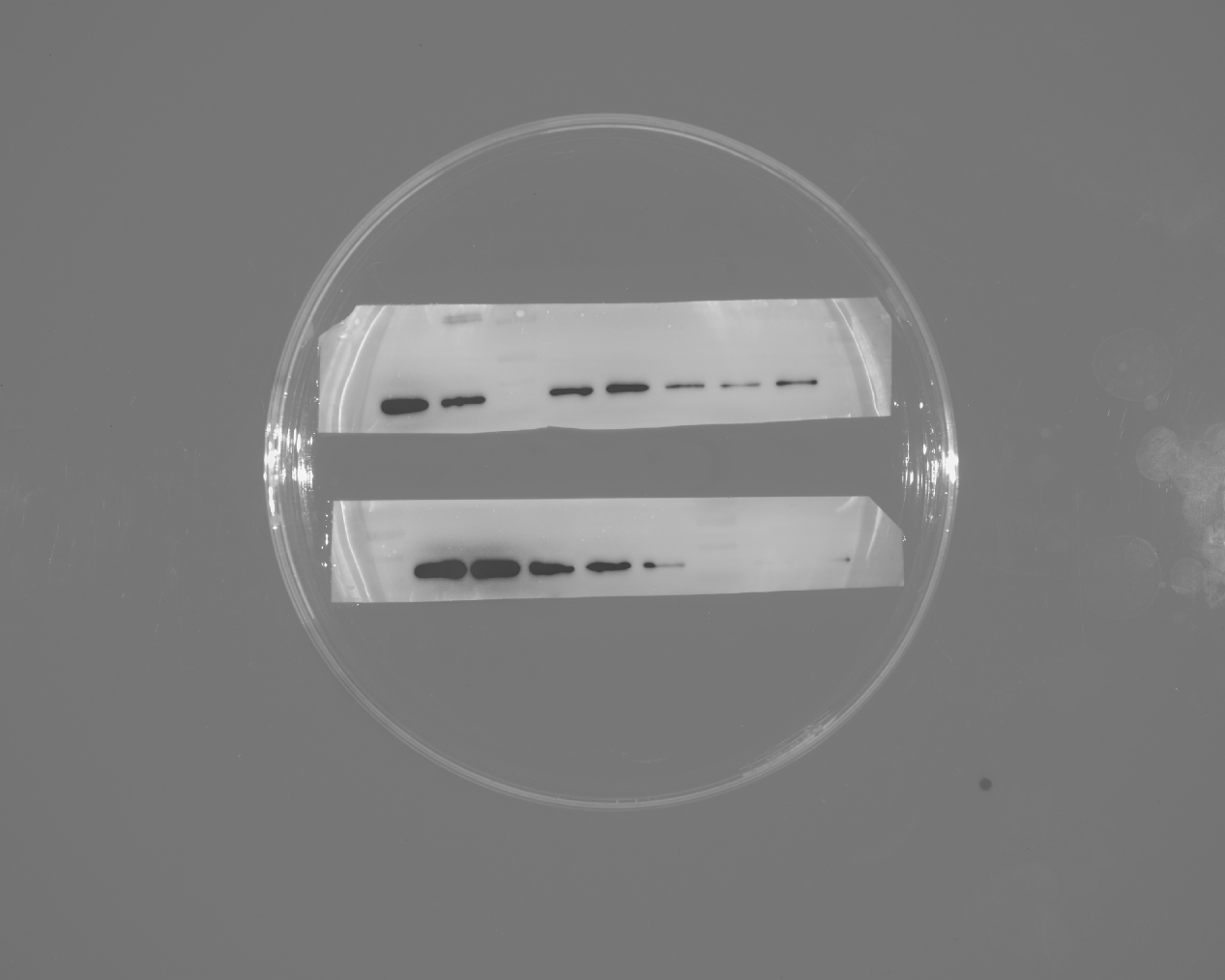


AGS-LC3


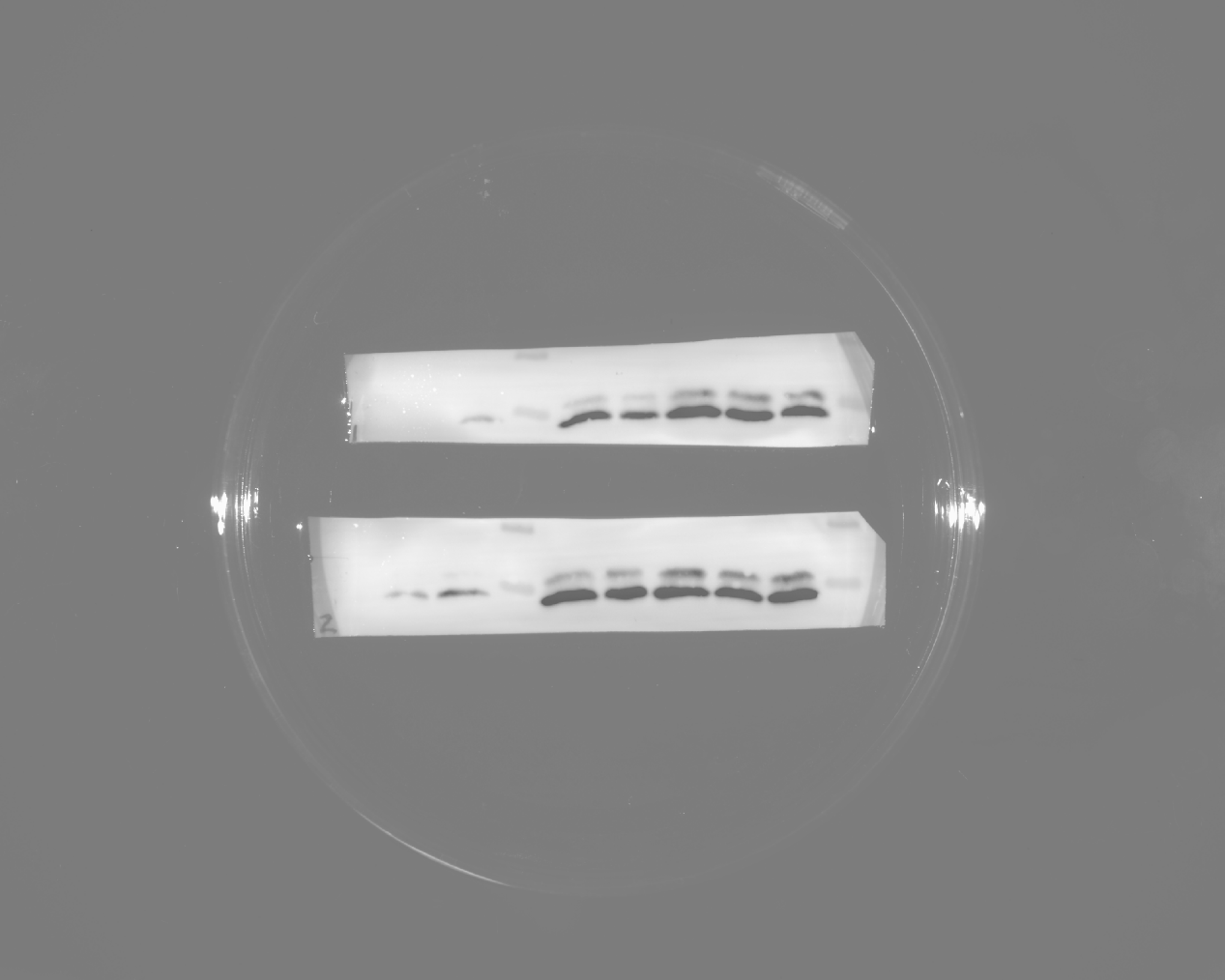


AGS-Actin


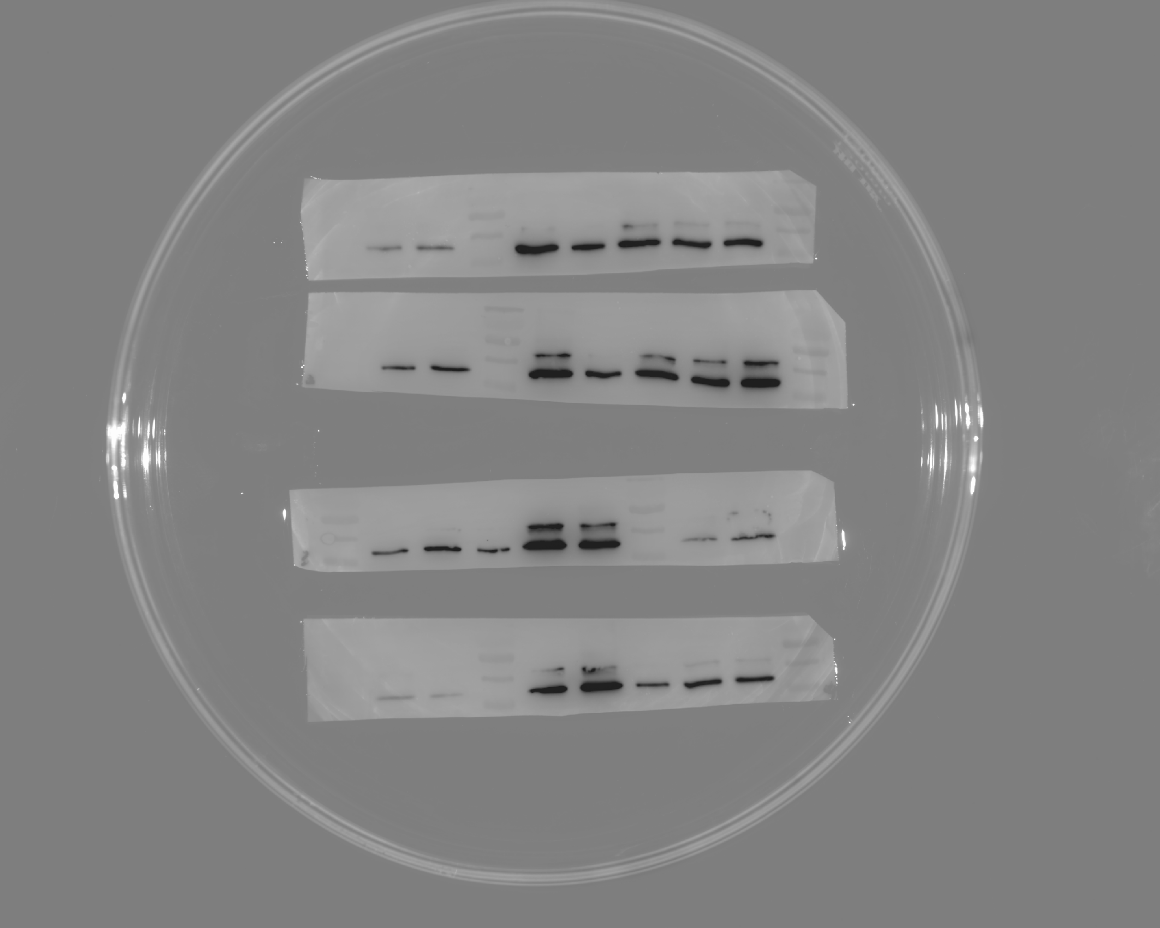


SGC-7901-P62


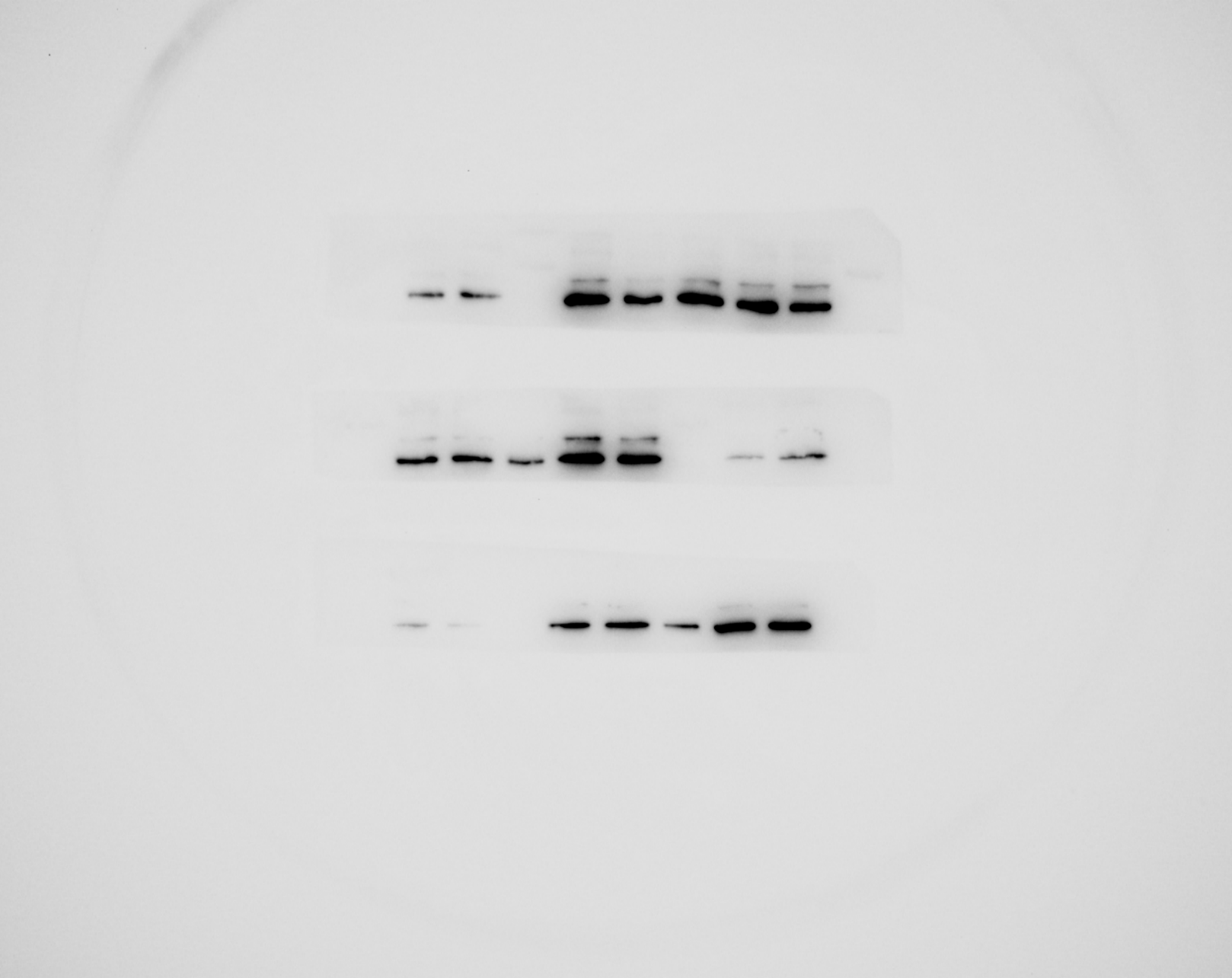


SGC-7901-LC3


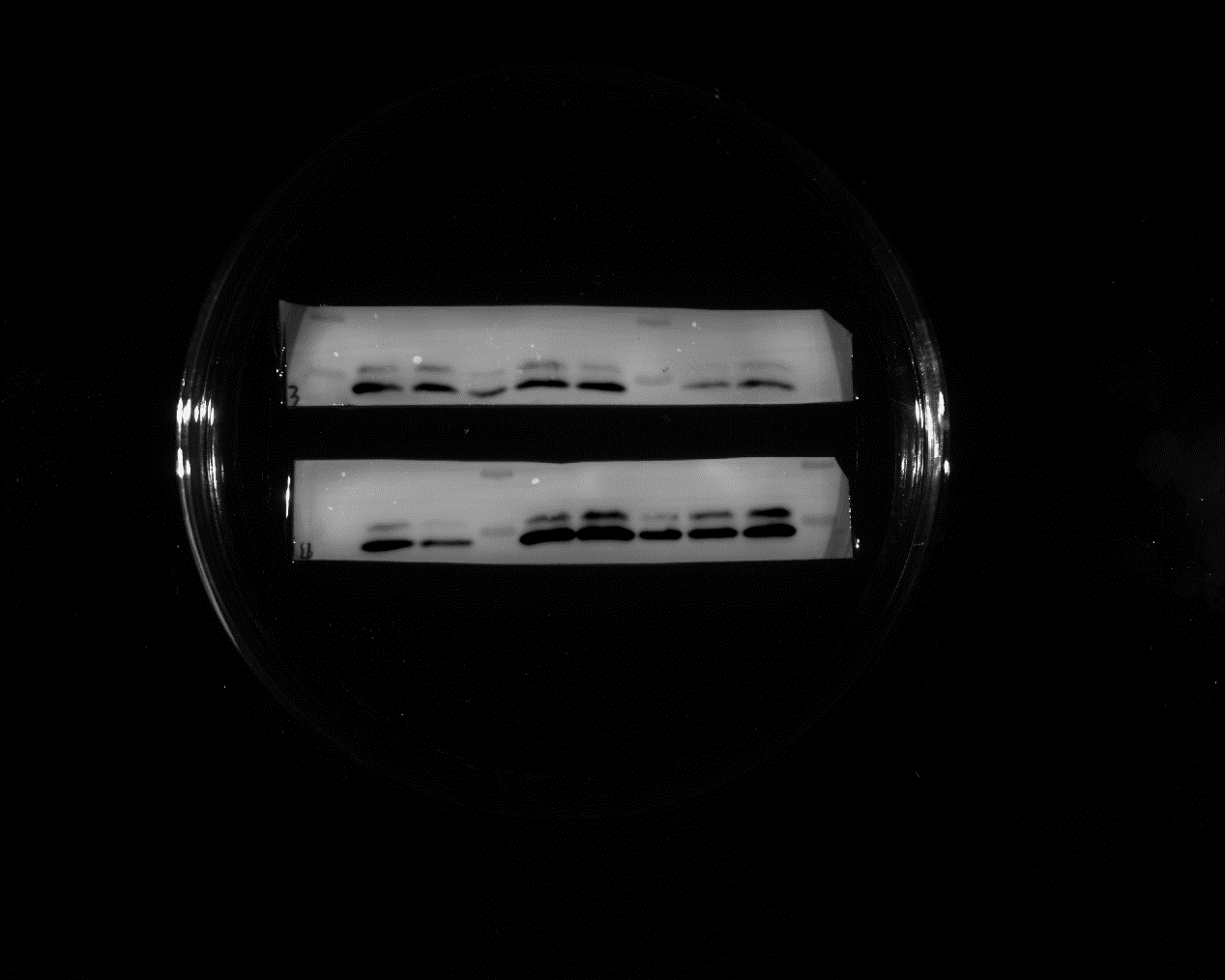


SGC-7901-Actin


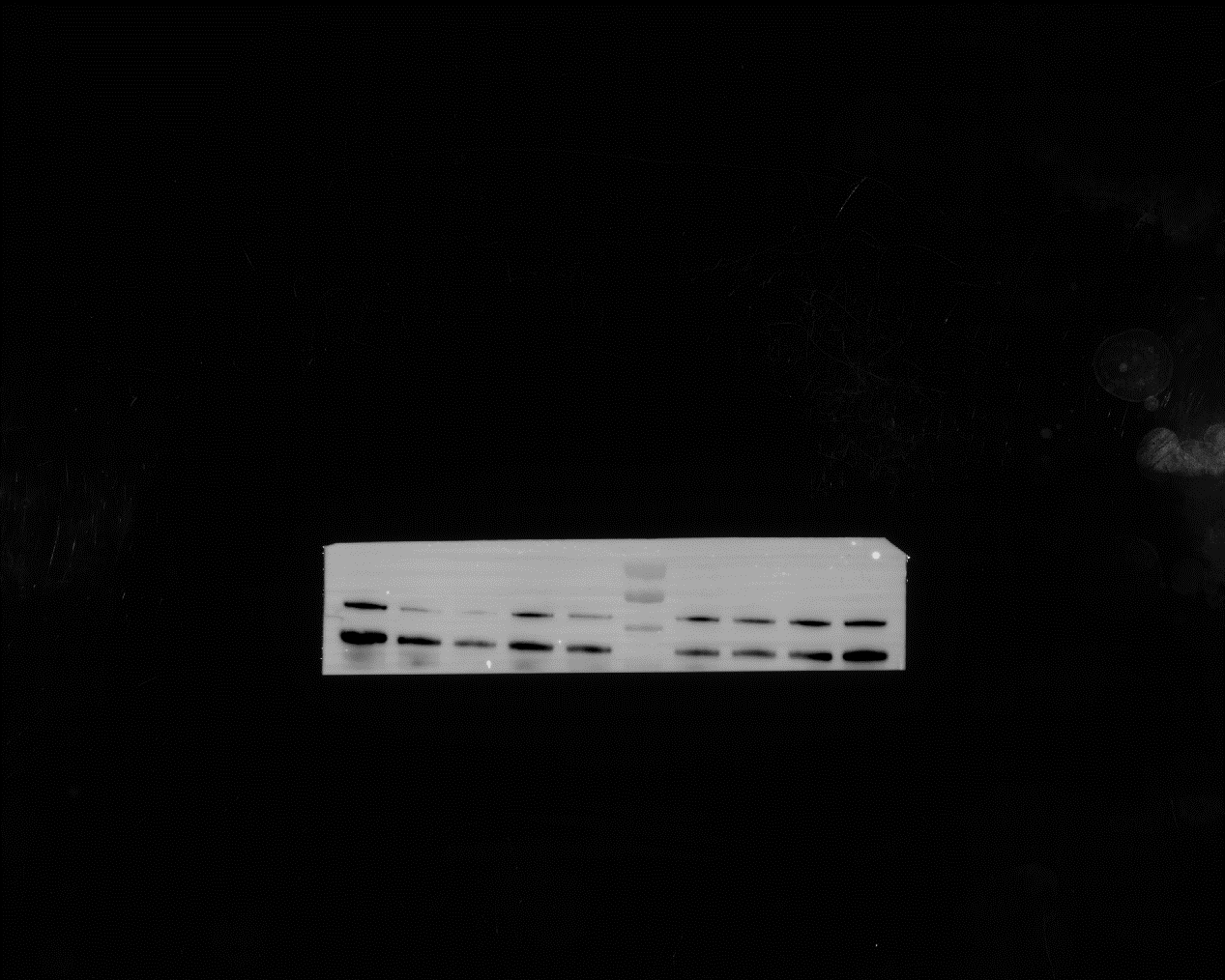


**FIGURE 4M**

AGS-E-cad


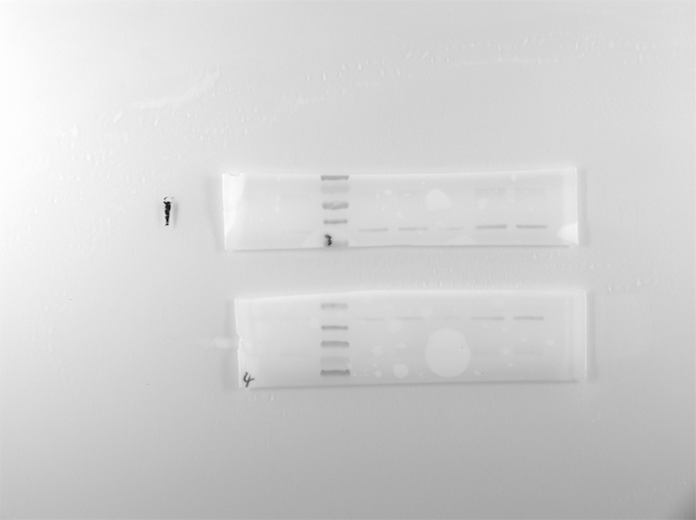


AGS-Vimentin


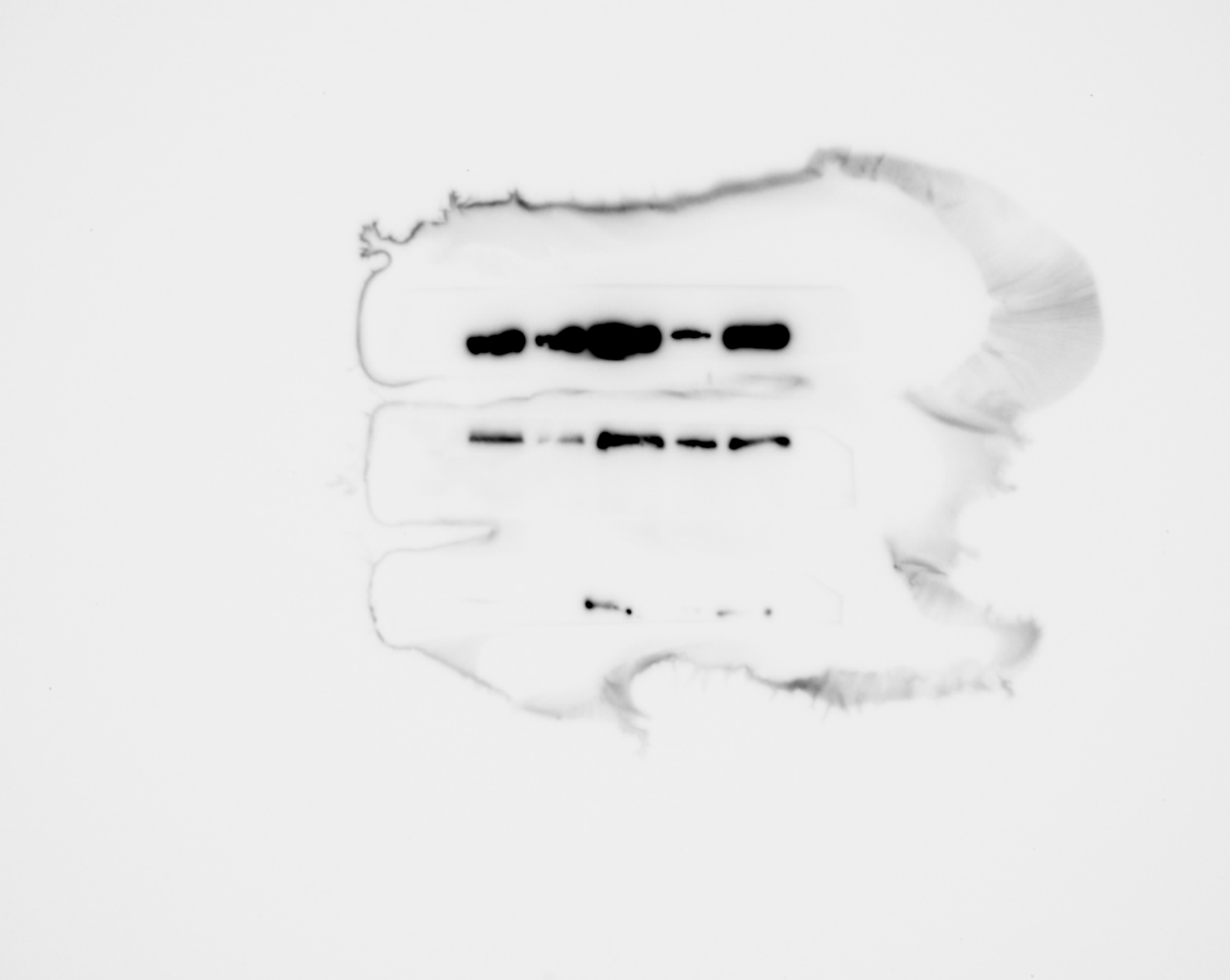


AGS-P62


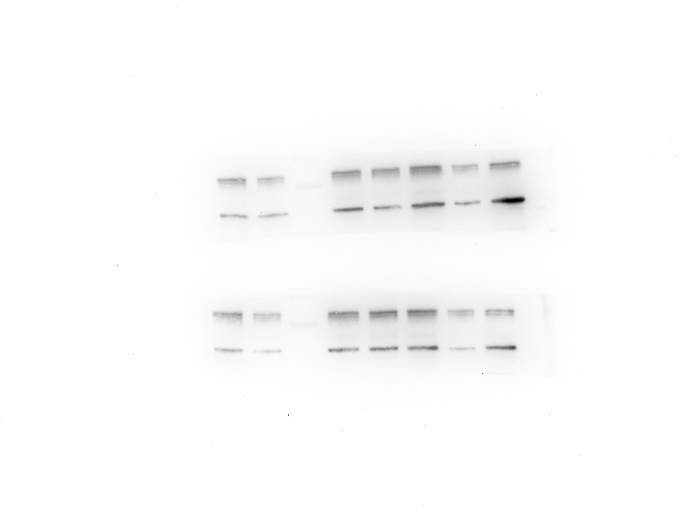


AGS-LC3


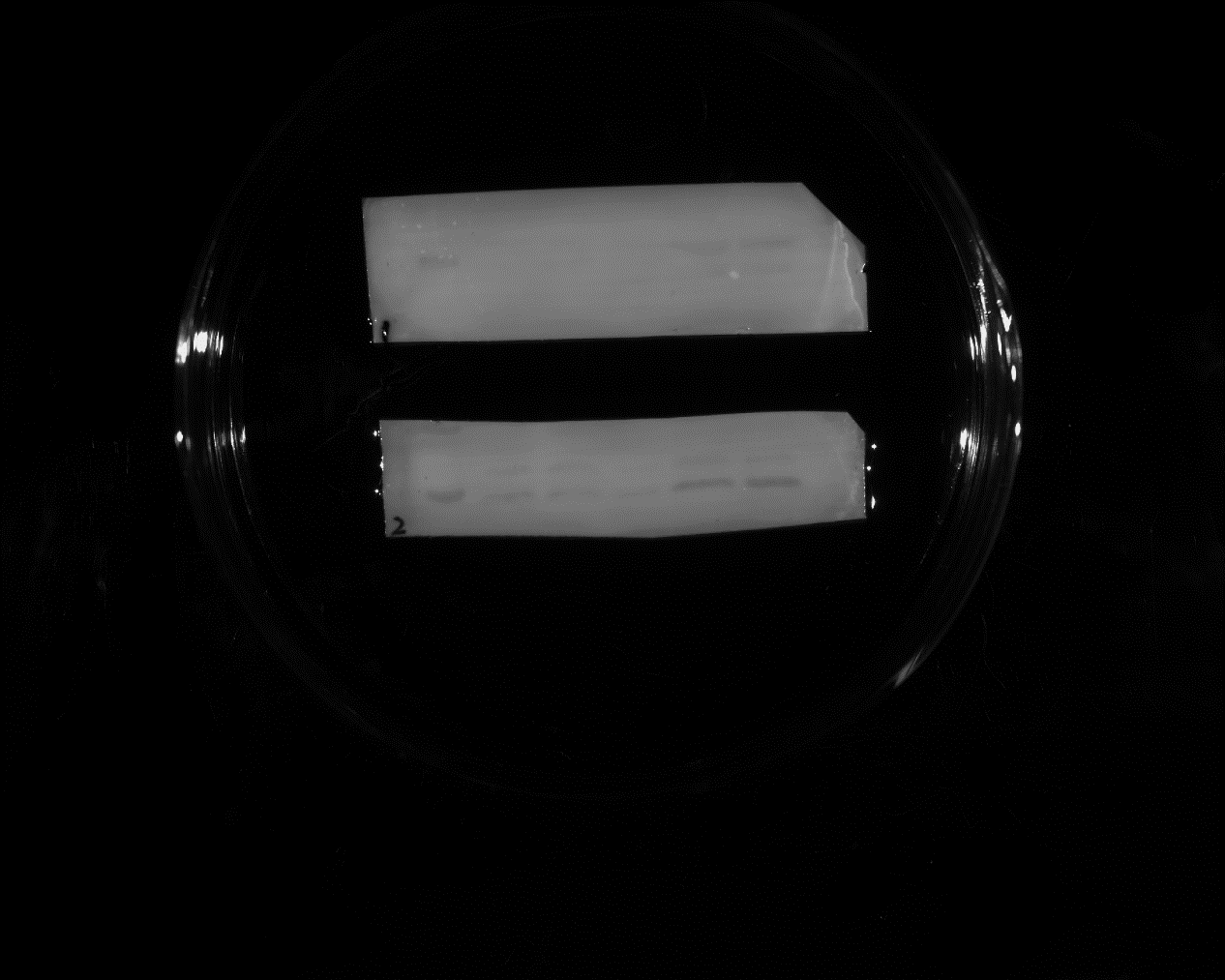


AGS-Actin


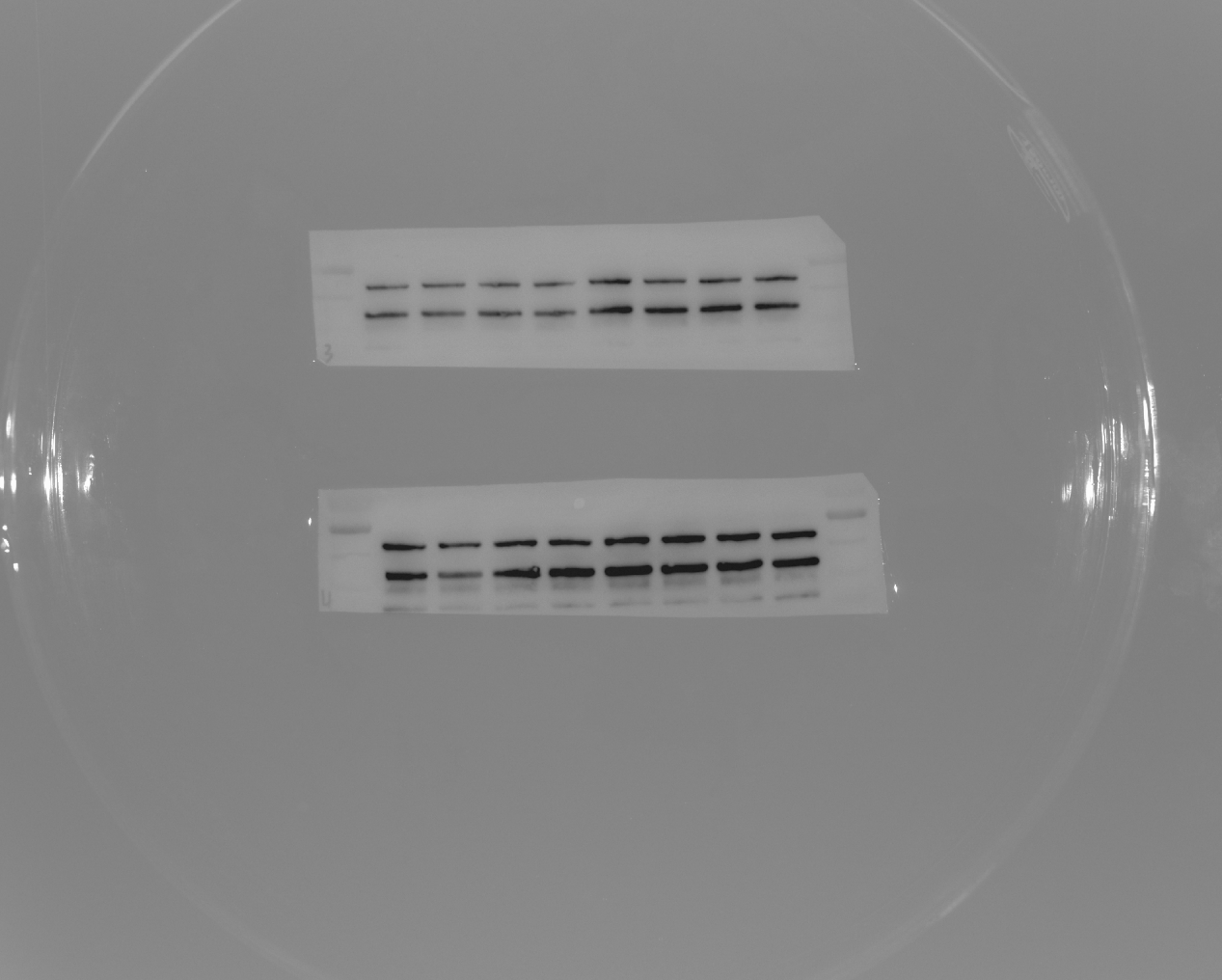


**FIGURE 4N**

SGC-7901-E-cad


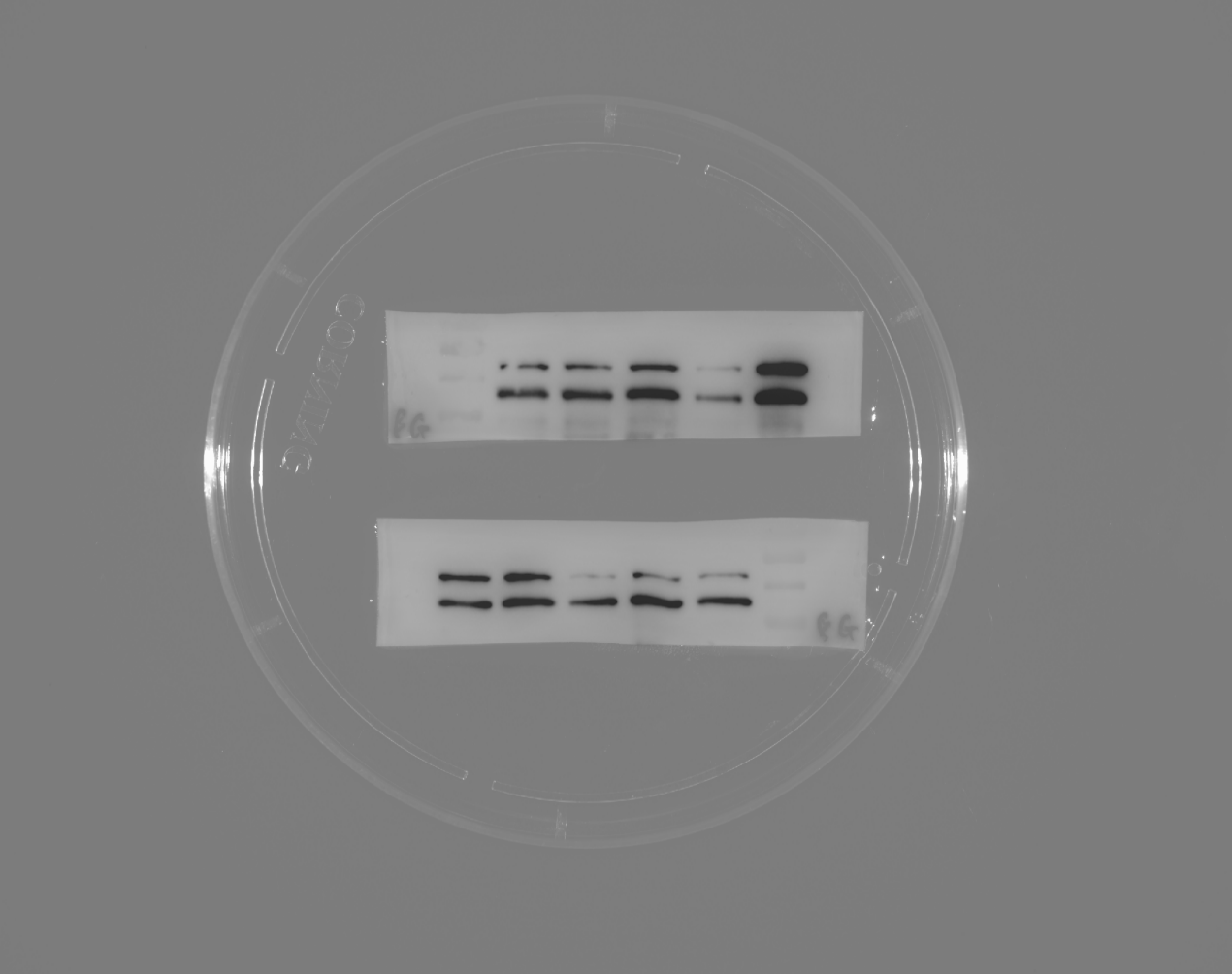


SGC-7901-Vimentin


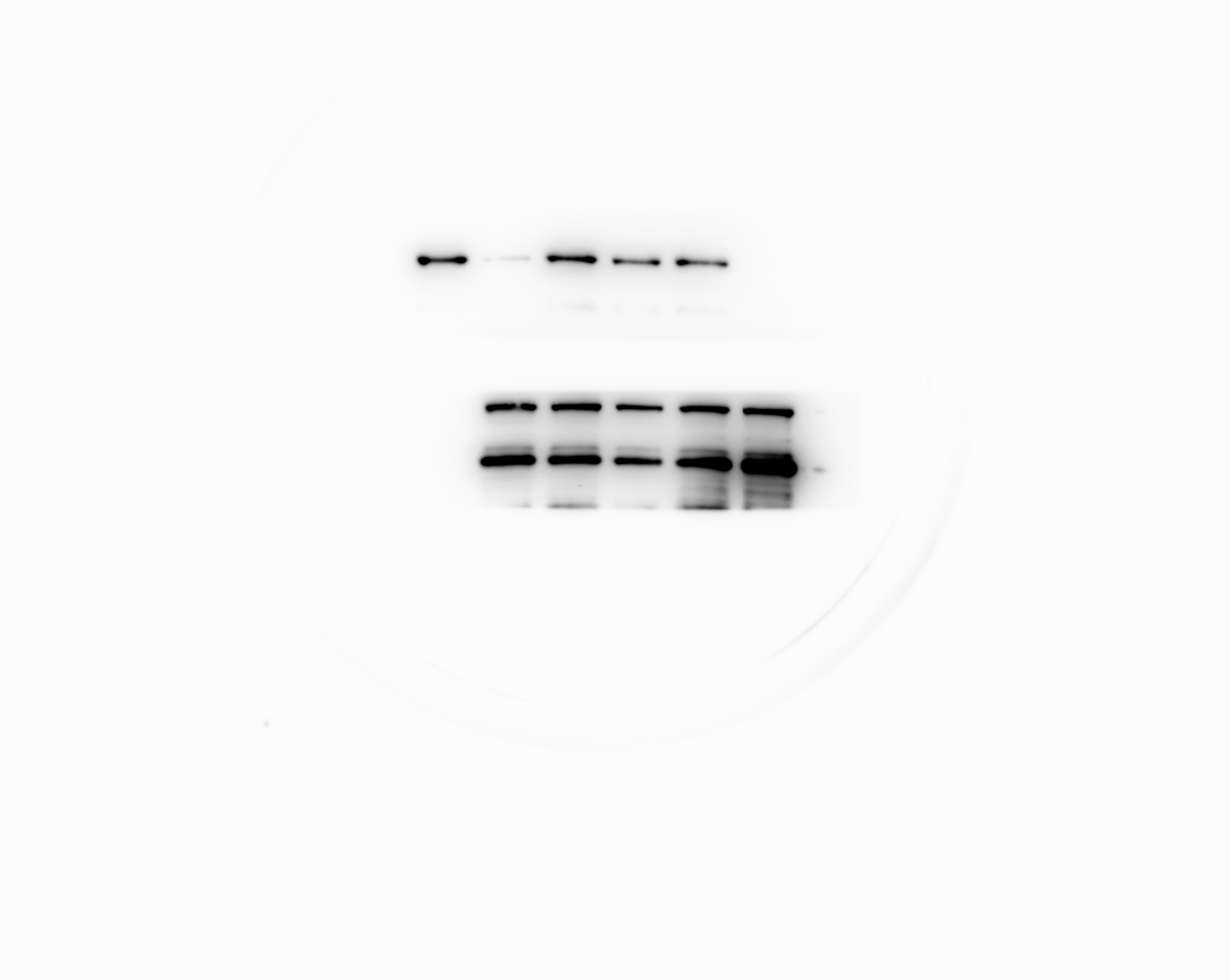


SGC-7901-P62


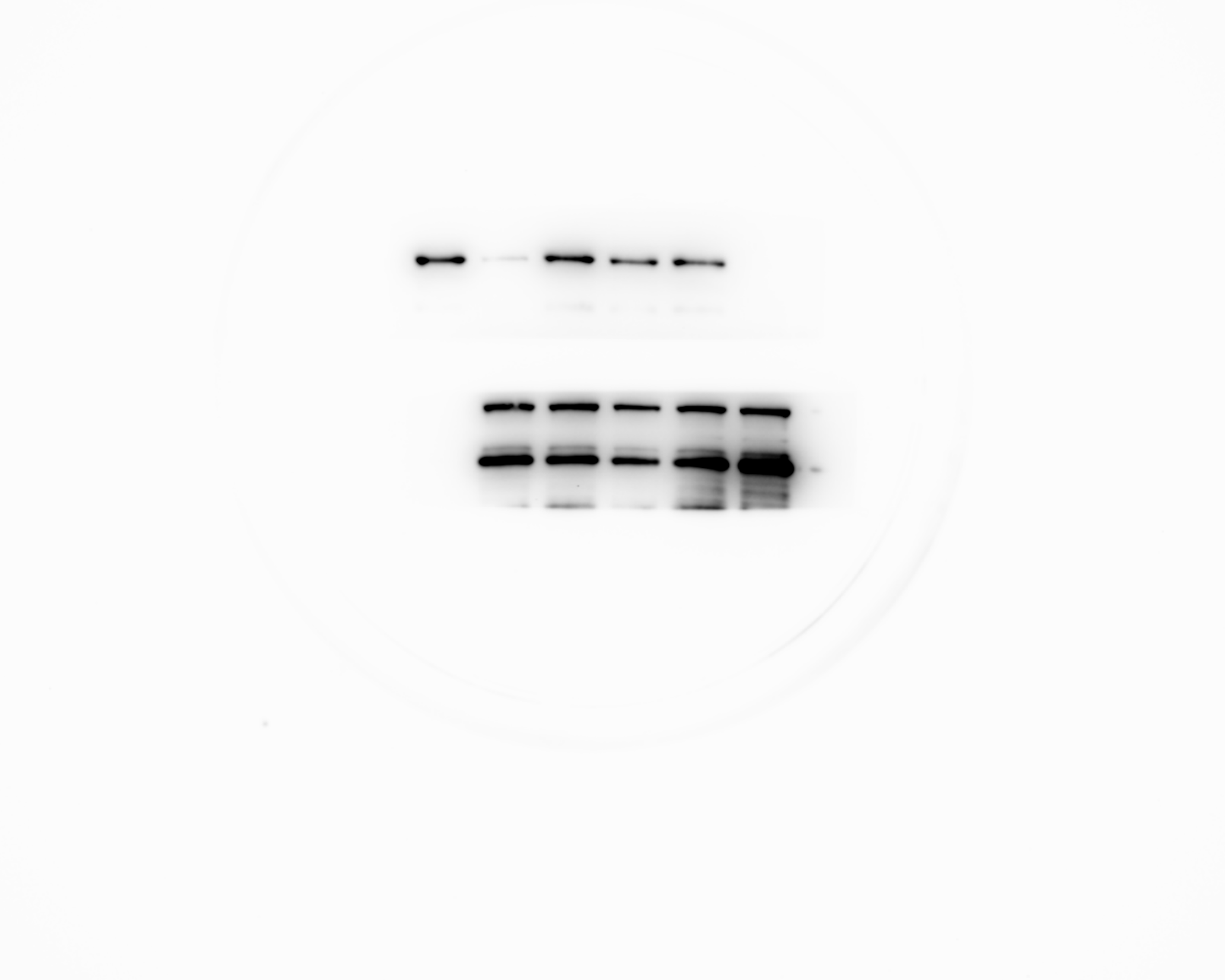


SGC-7901-LC3


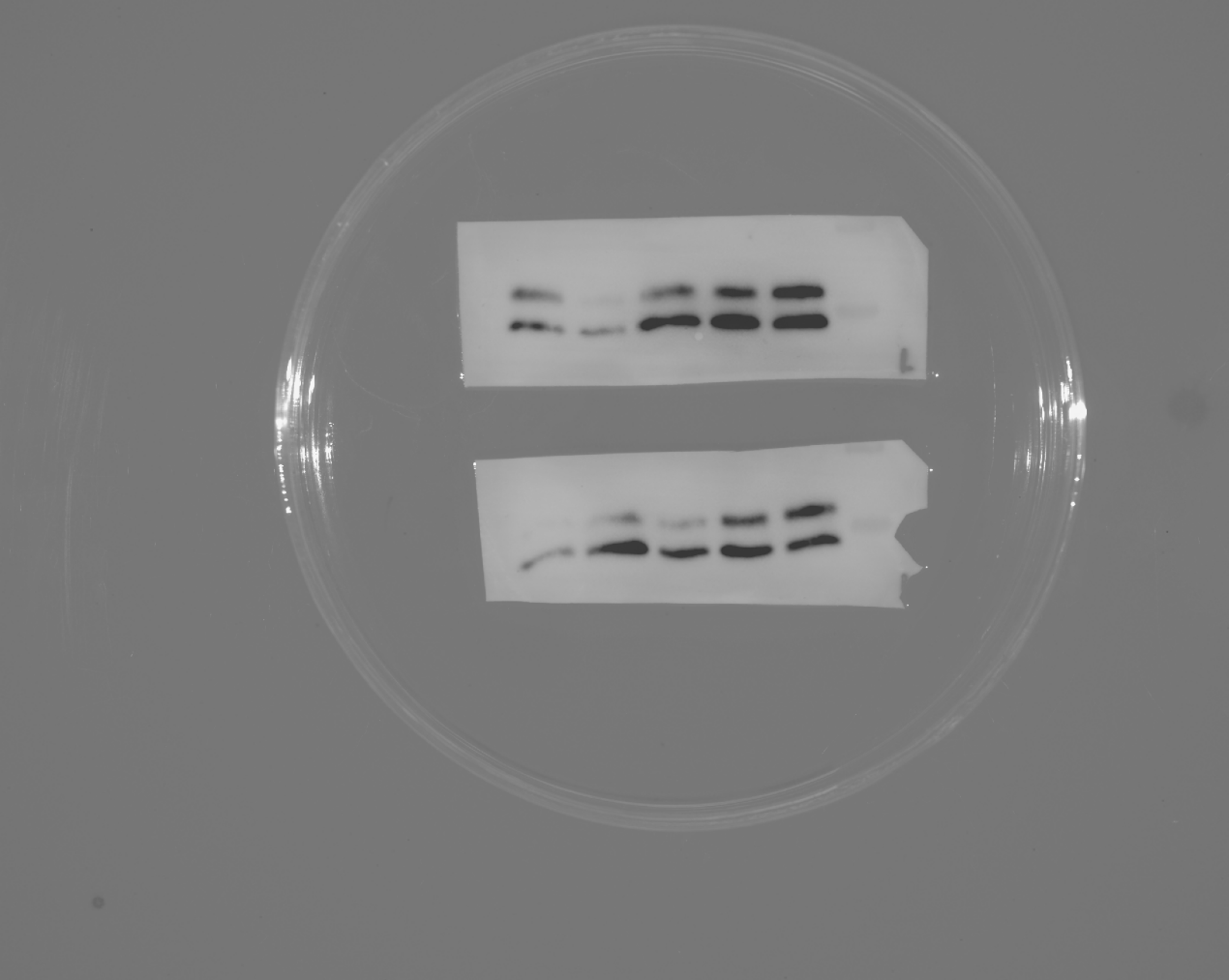


SGC-7901-Actin


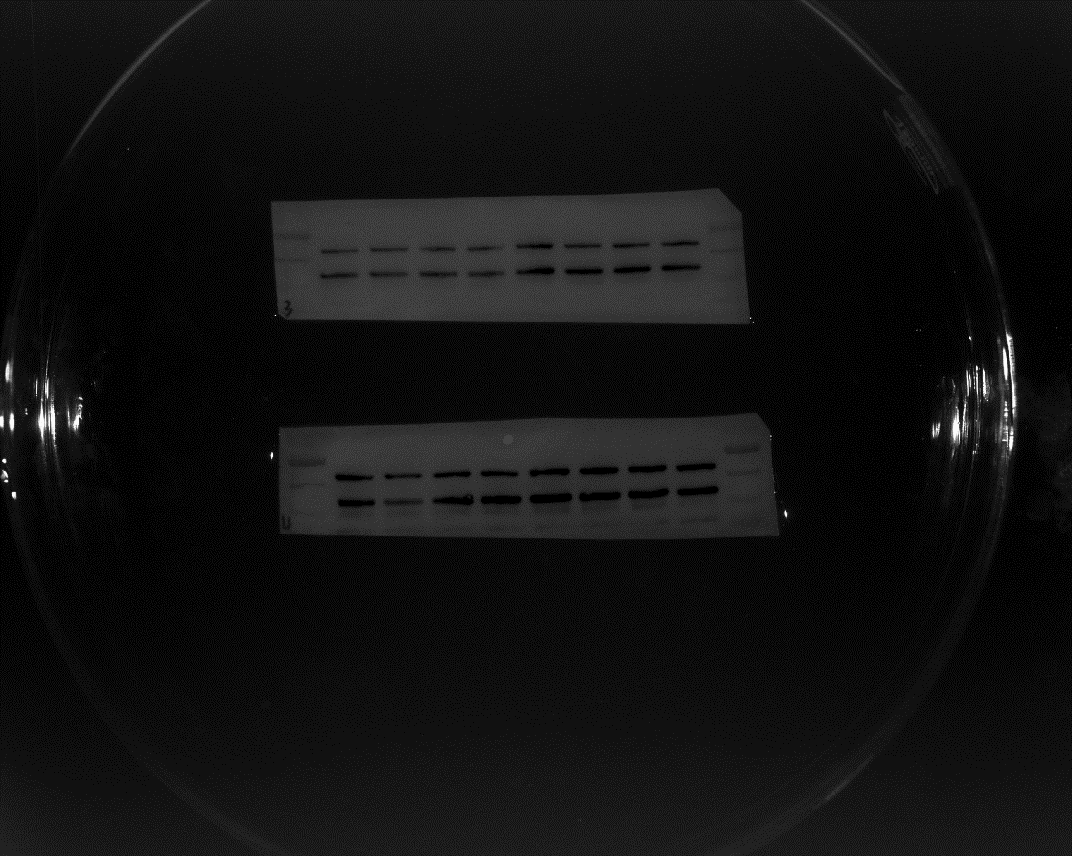


FIGURE 6I

AGS-E-cad


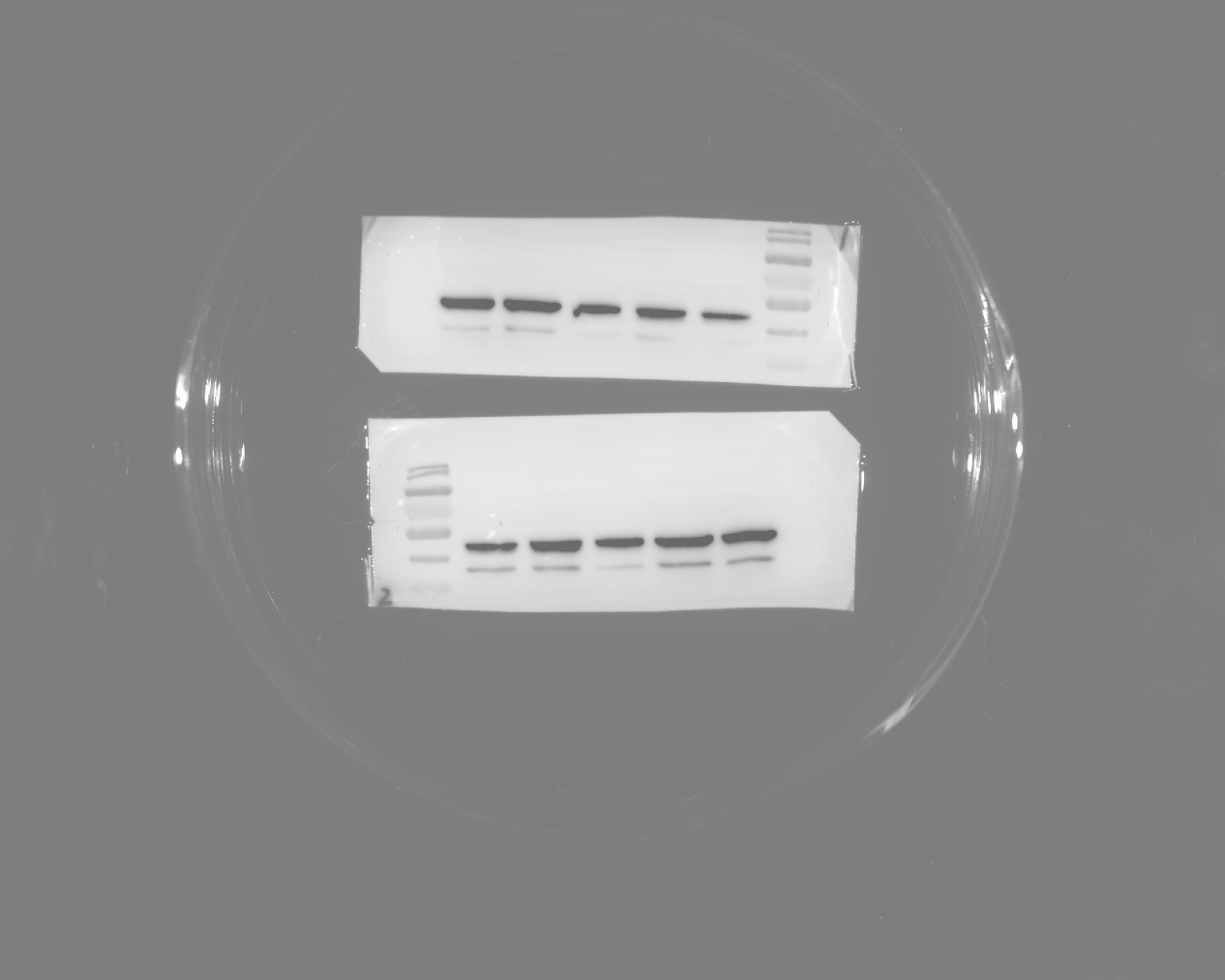


AGS-Vimentin


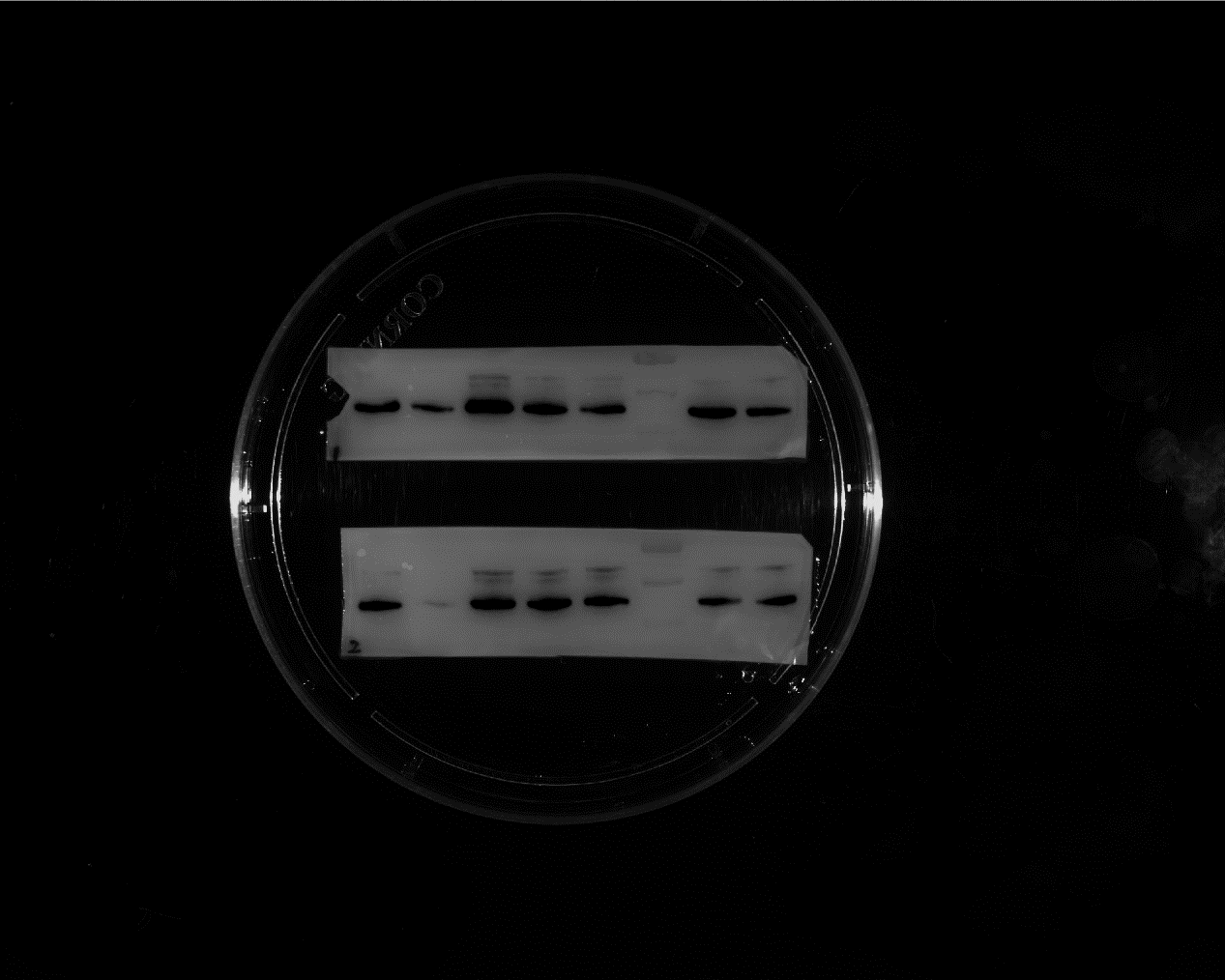


AGS-P62


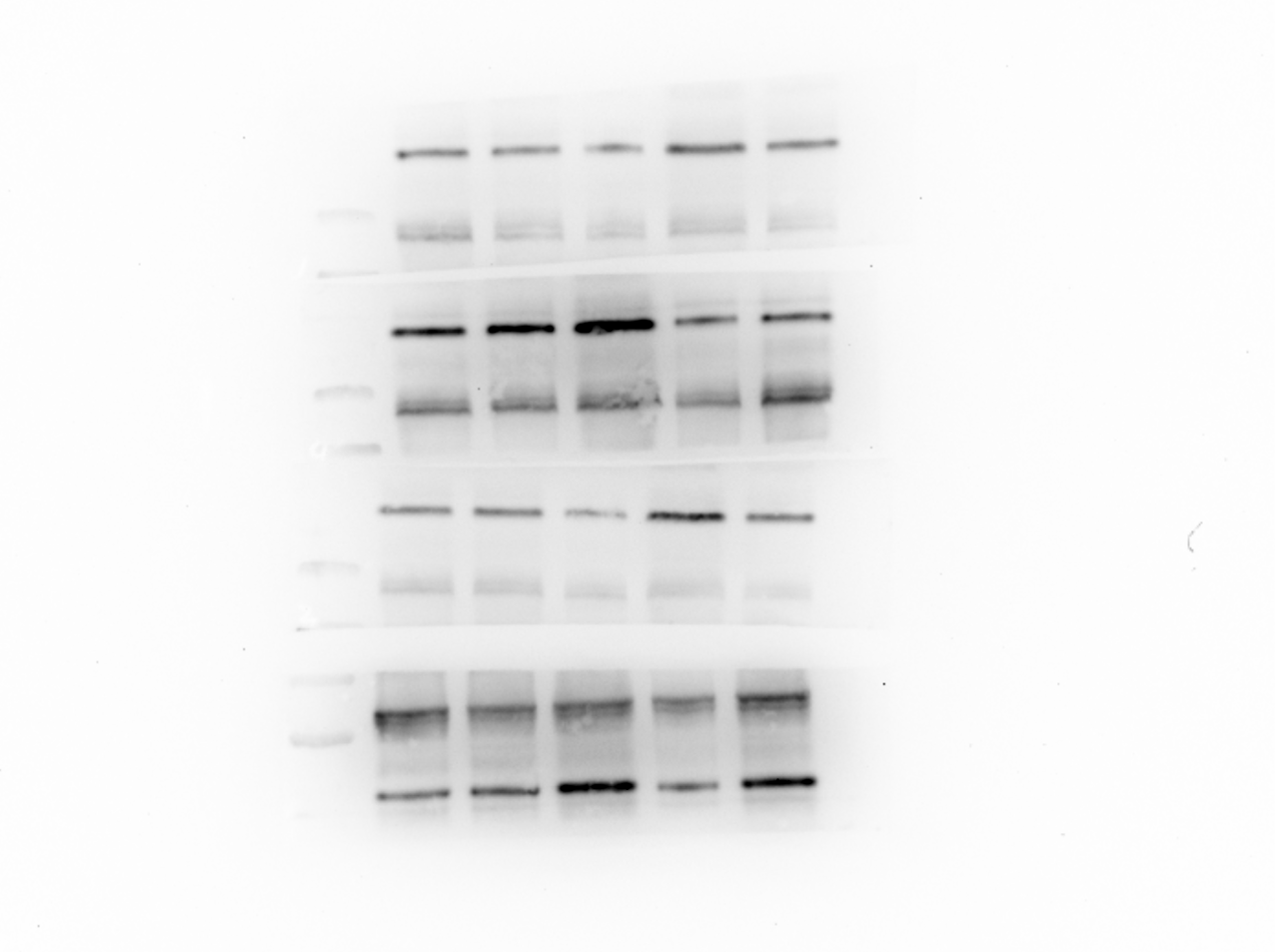


AGS-LC3


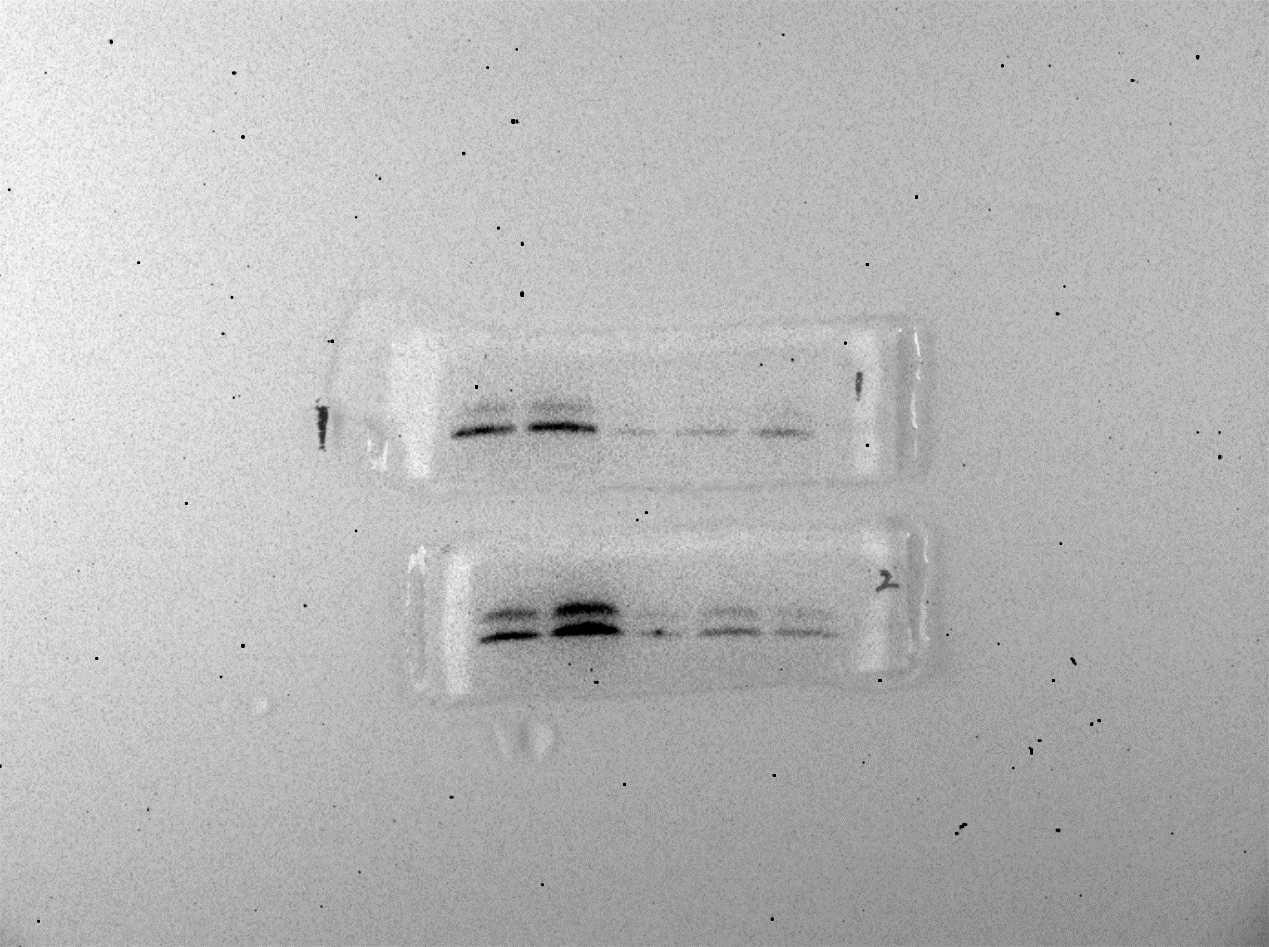


AGS-Actin


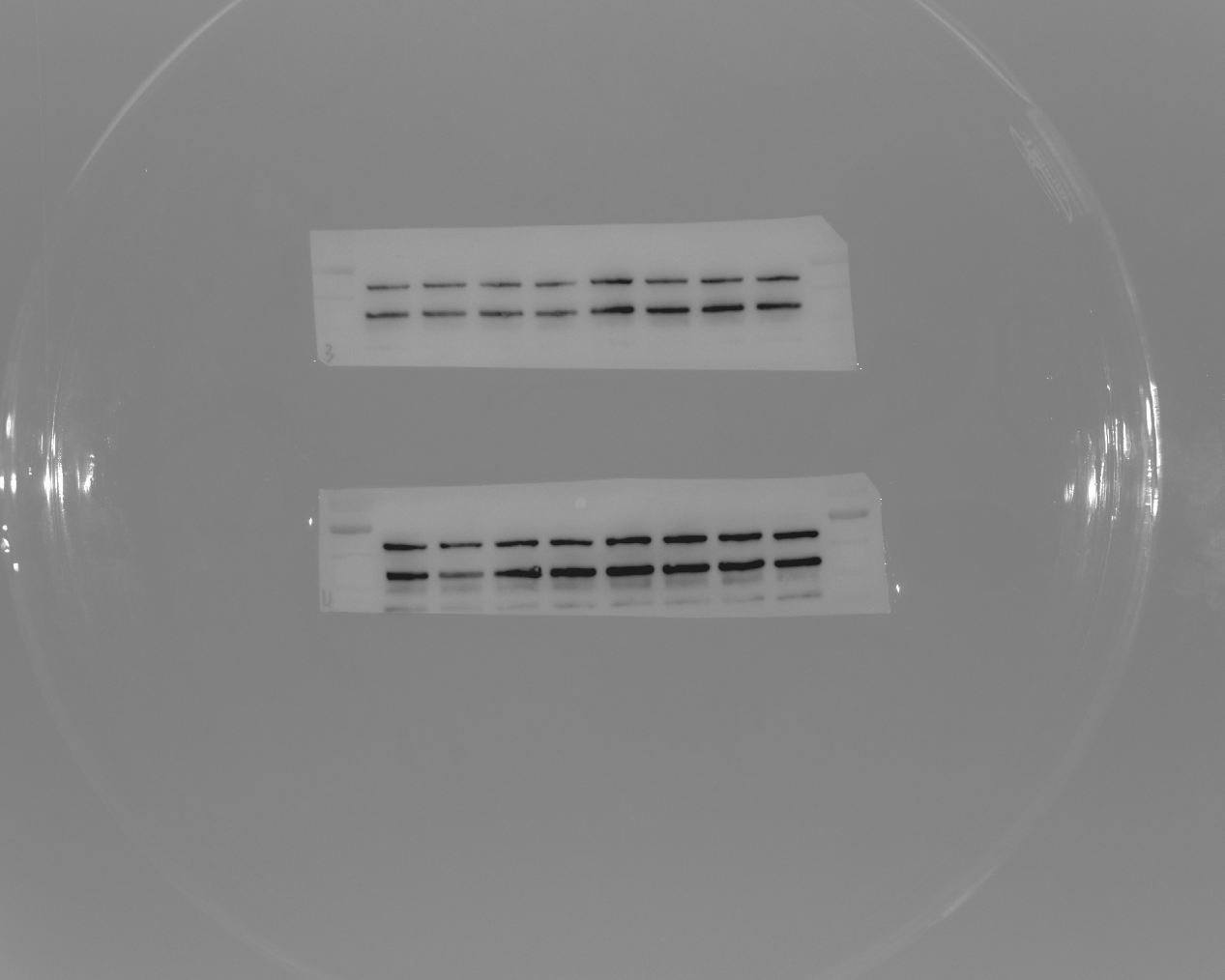


SGC-7901-E-cad


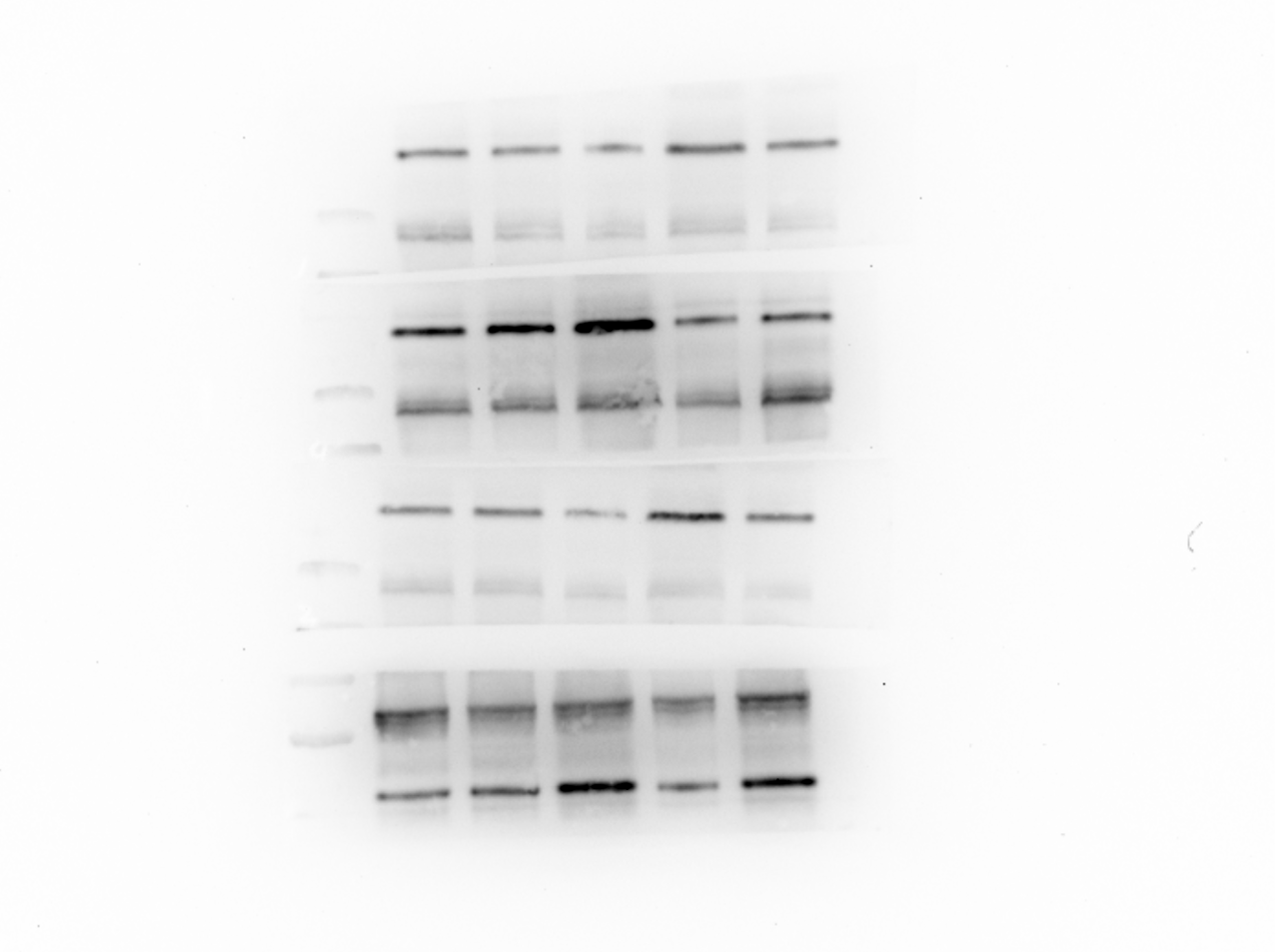


SGC-7901-Vimentin


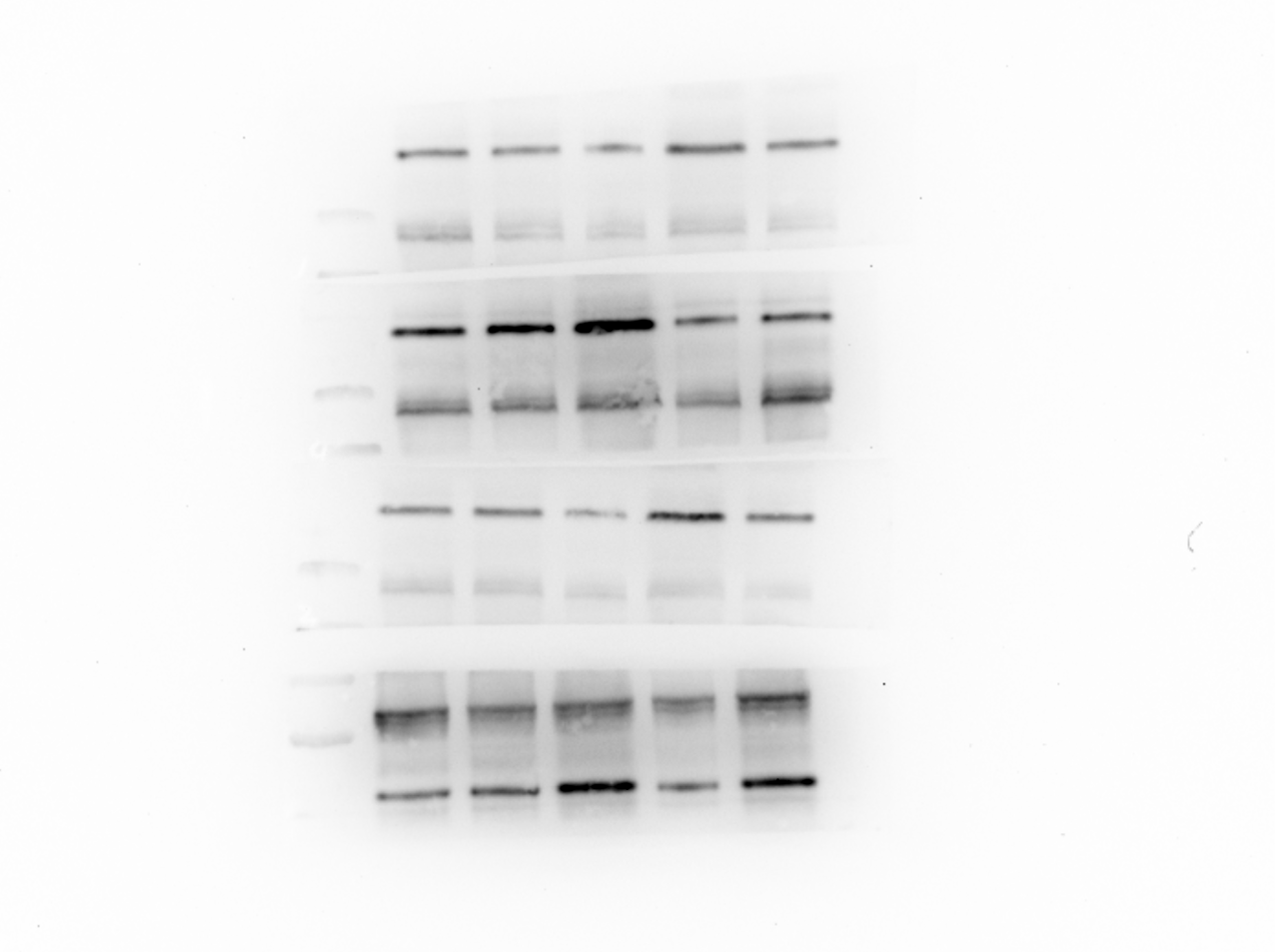


SGC-7901-P62


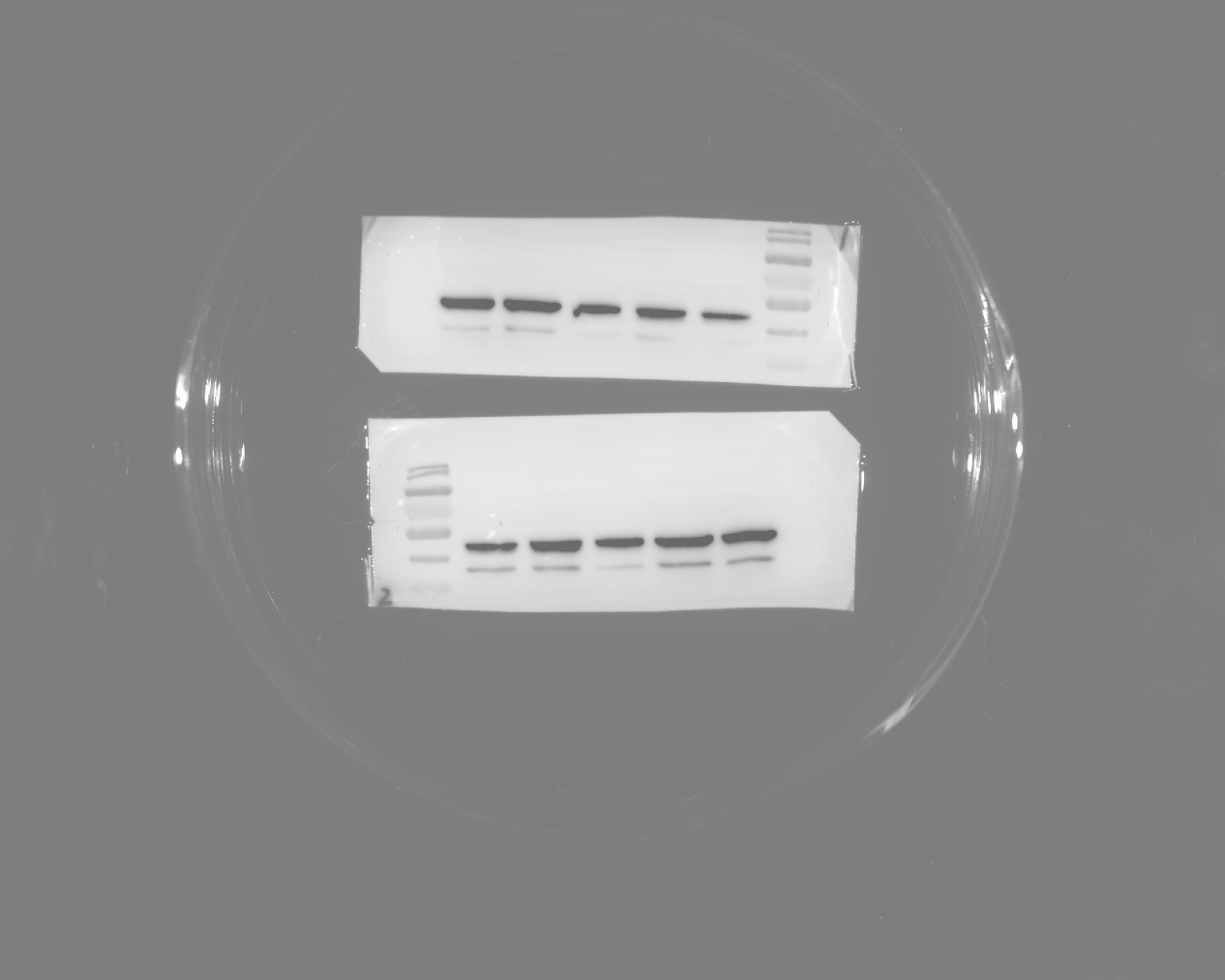


SGC-7901-LC3


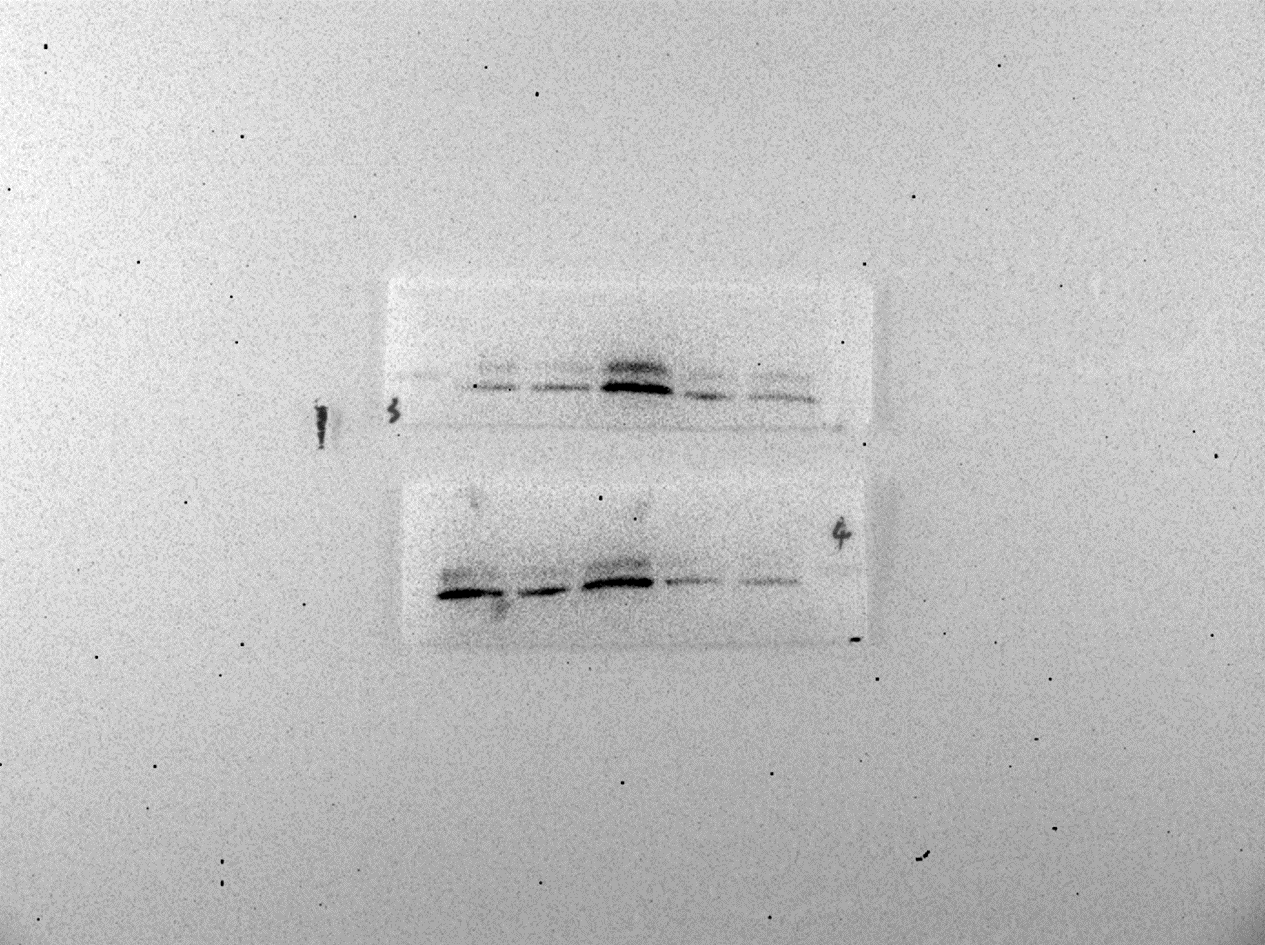


SGC-7901-Actin


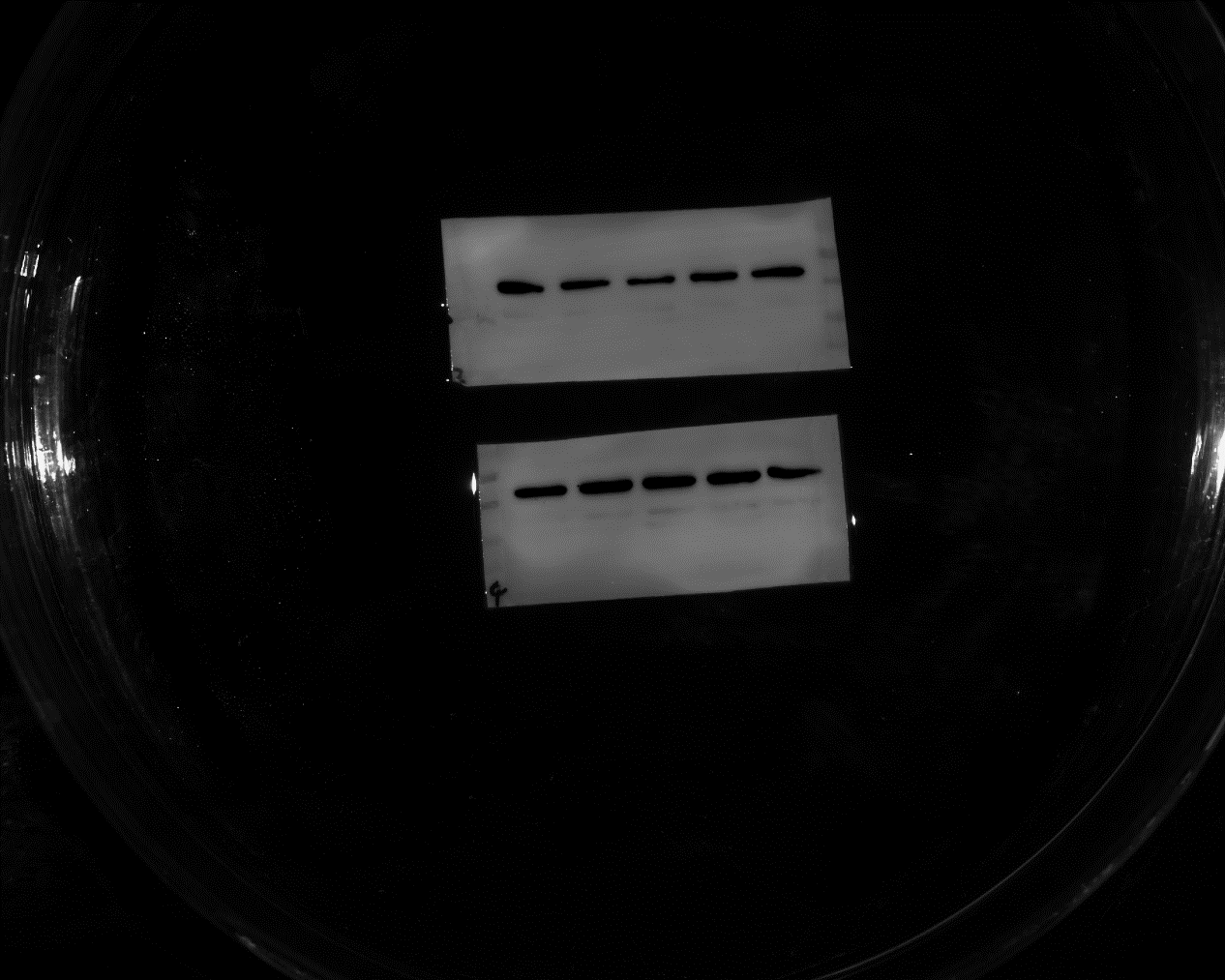


FIGURE 6J

AGS-PAK1


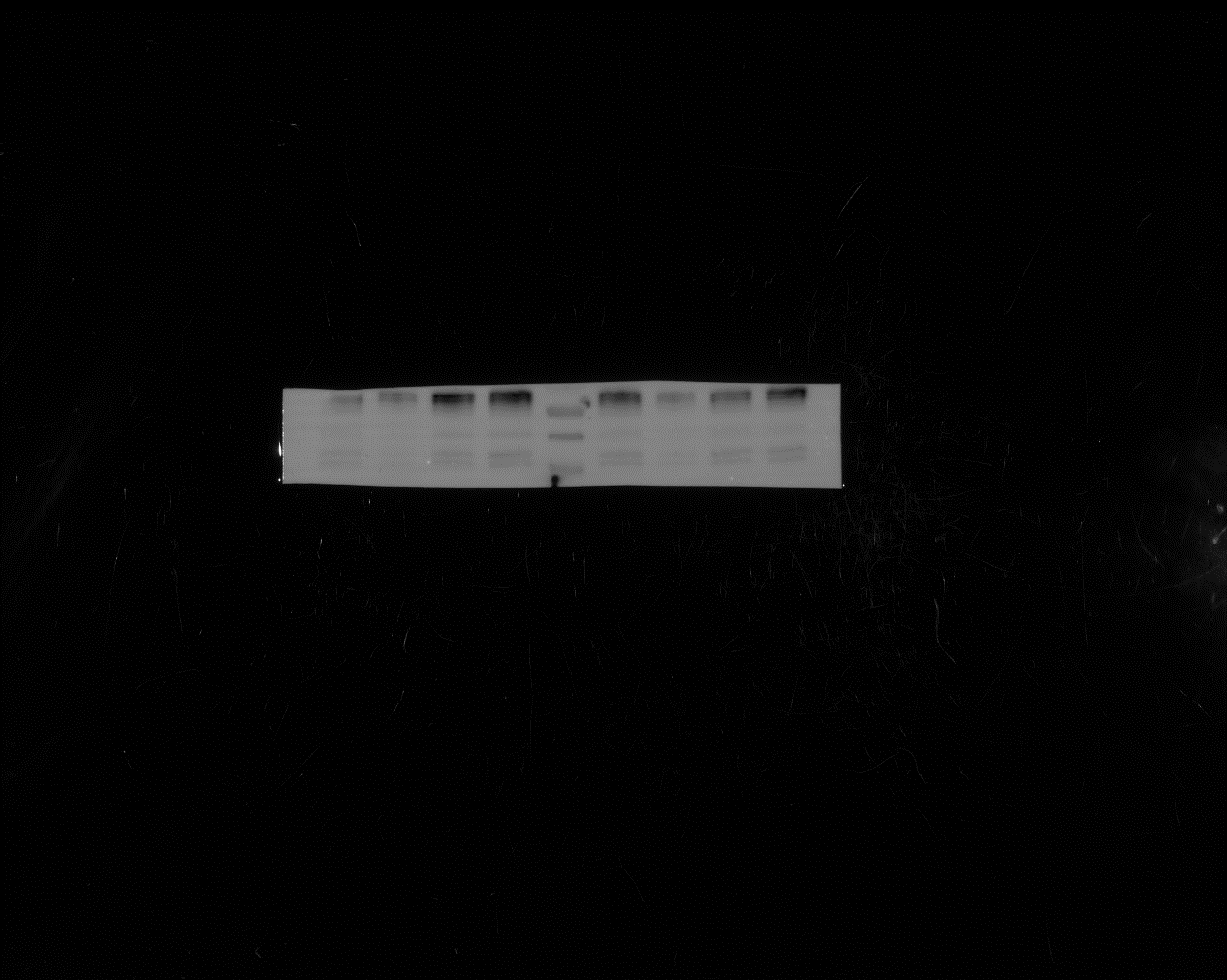


AGS-p-AKT


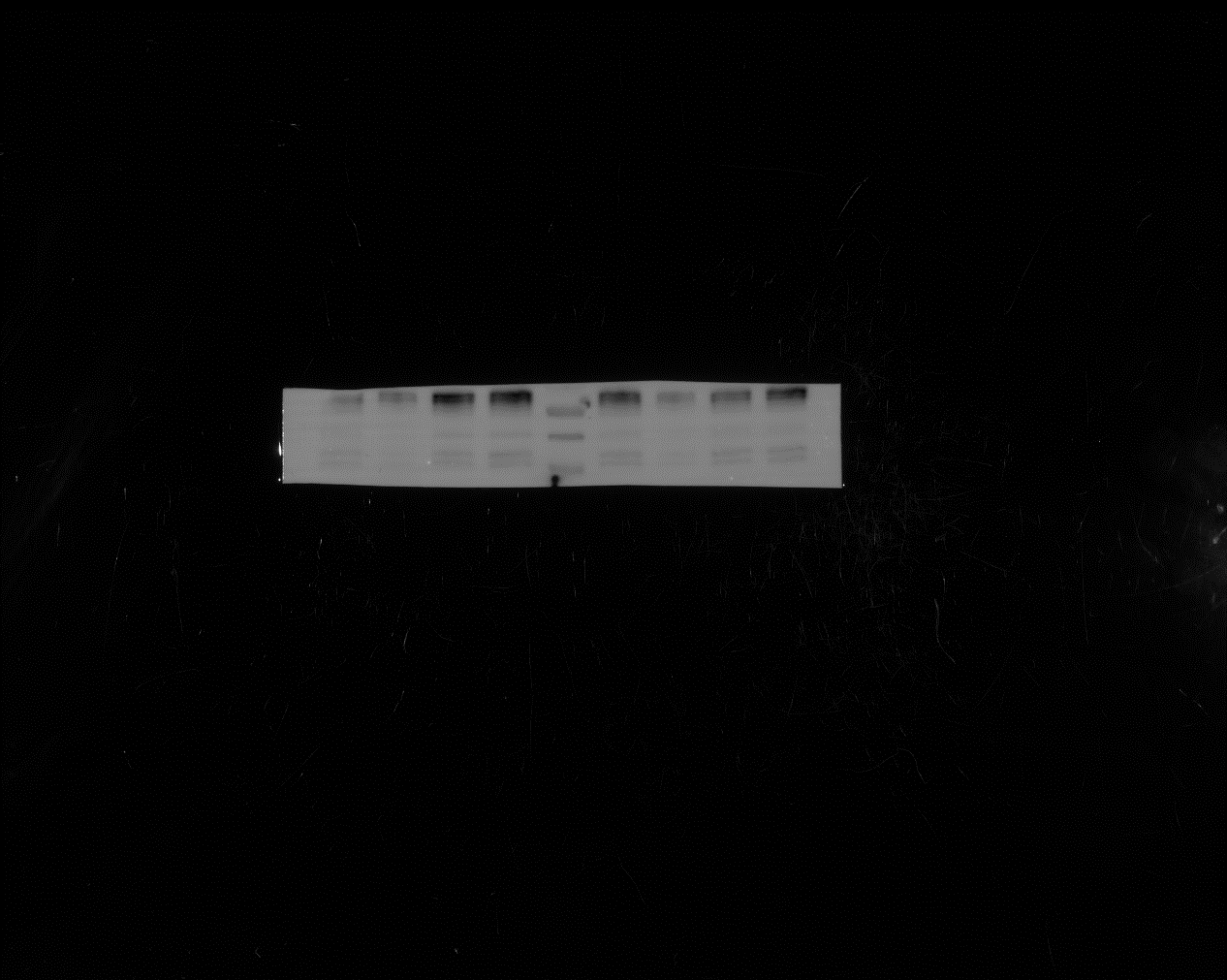


AGS-AKT


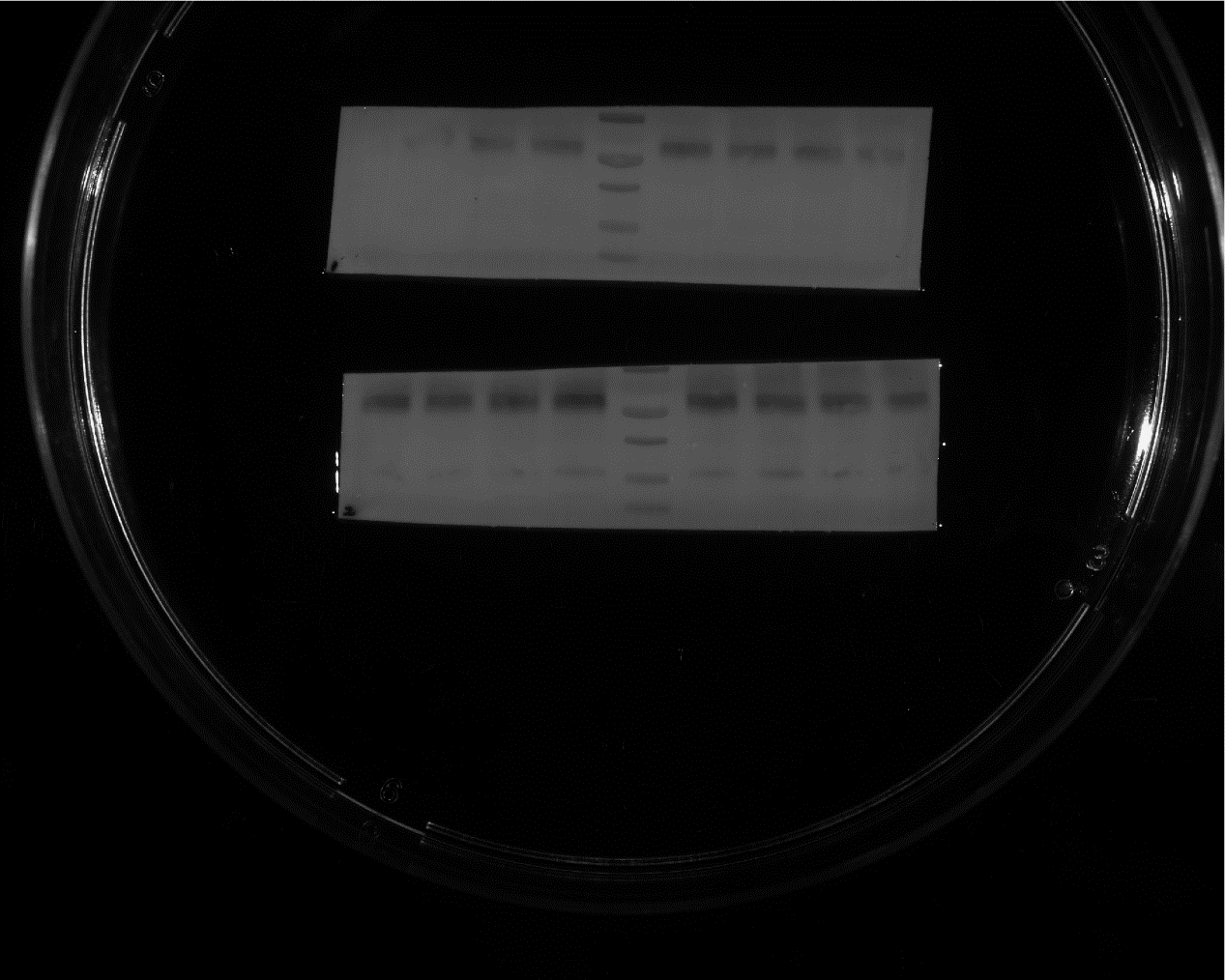


AGS-p-ERK


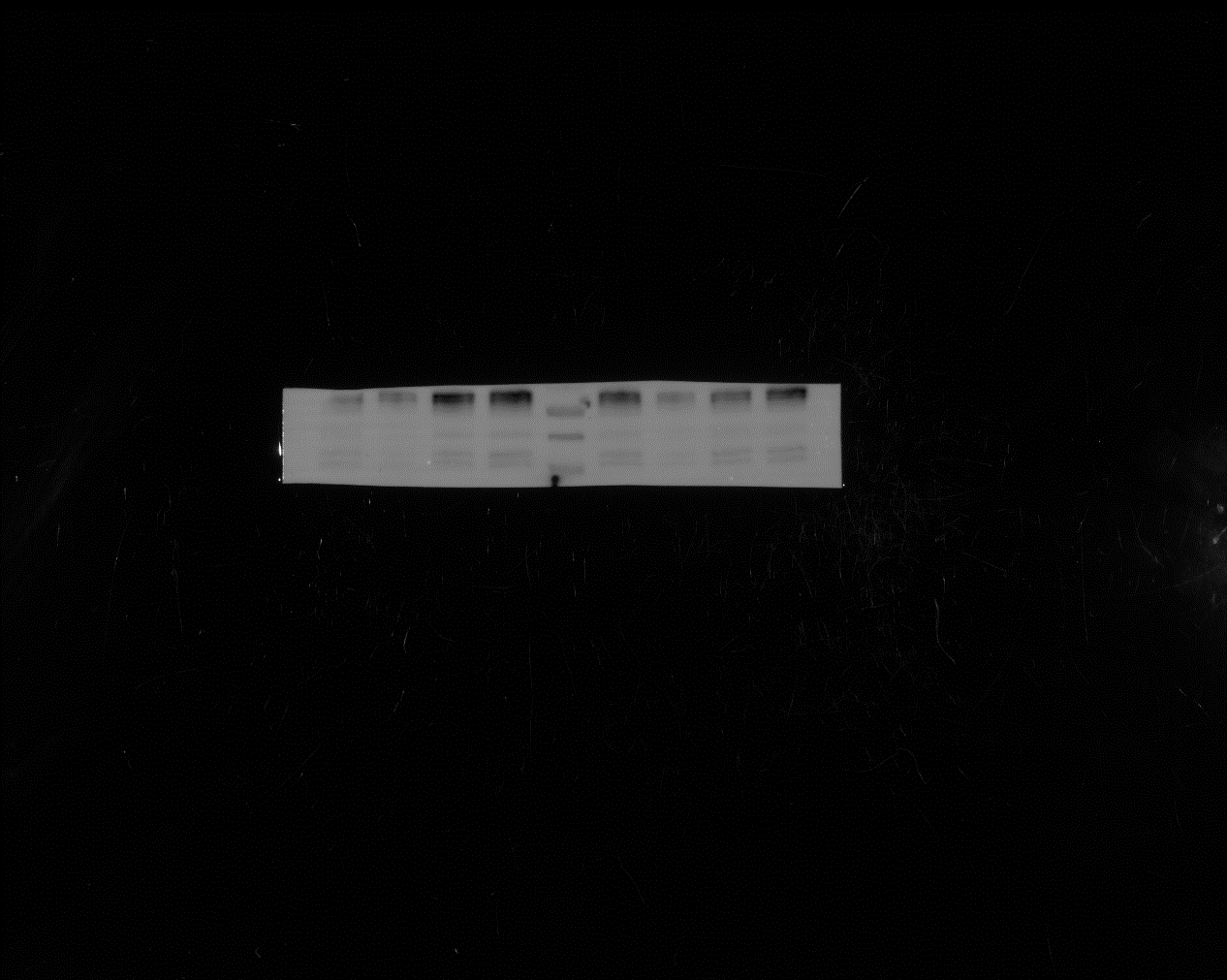


AGS-ERK


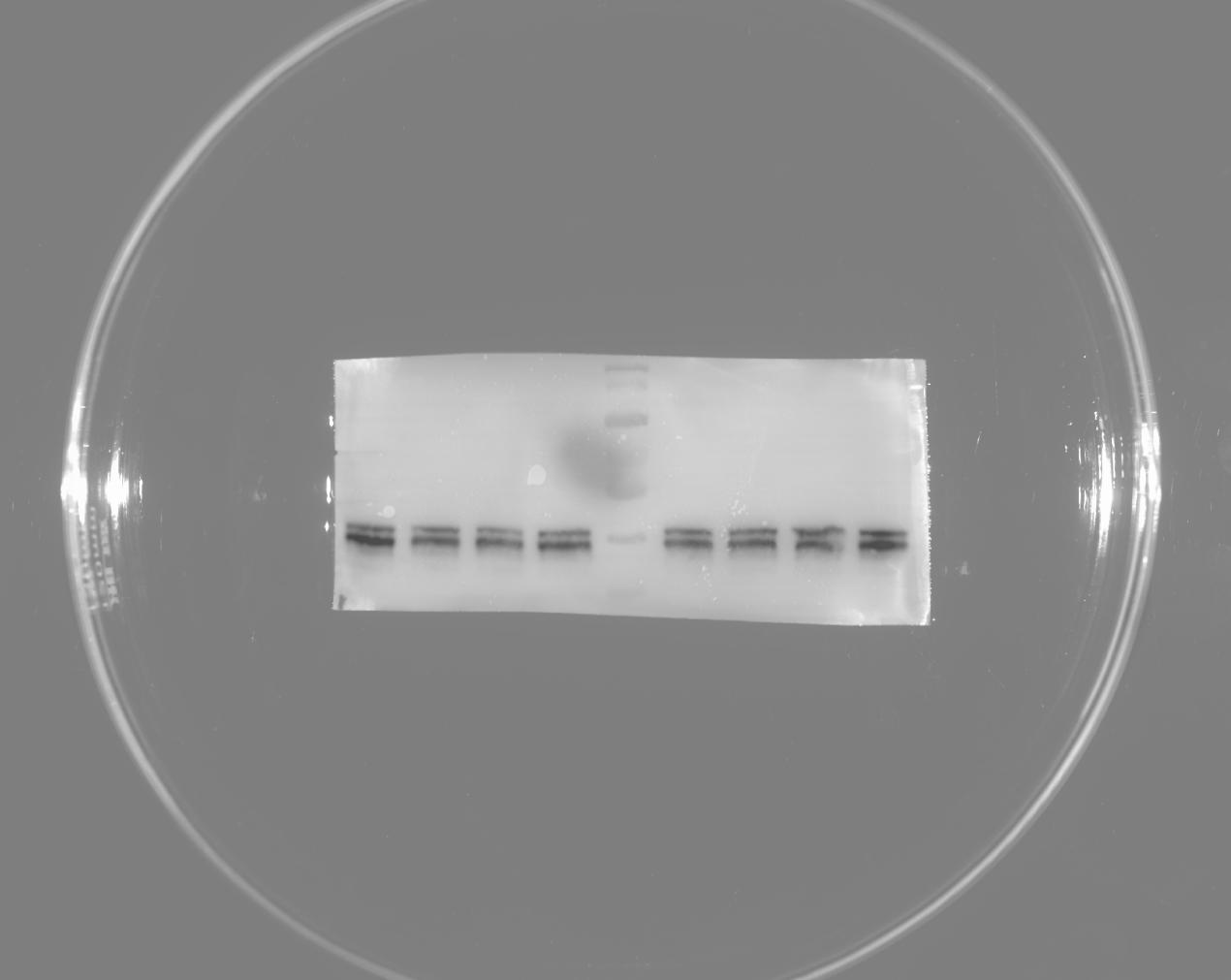


AGS-Actin


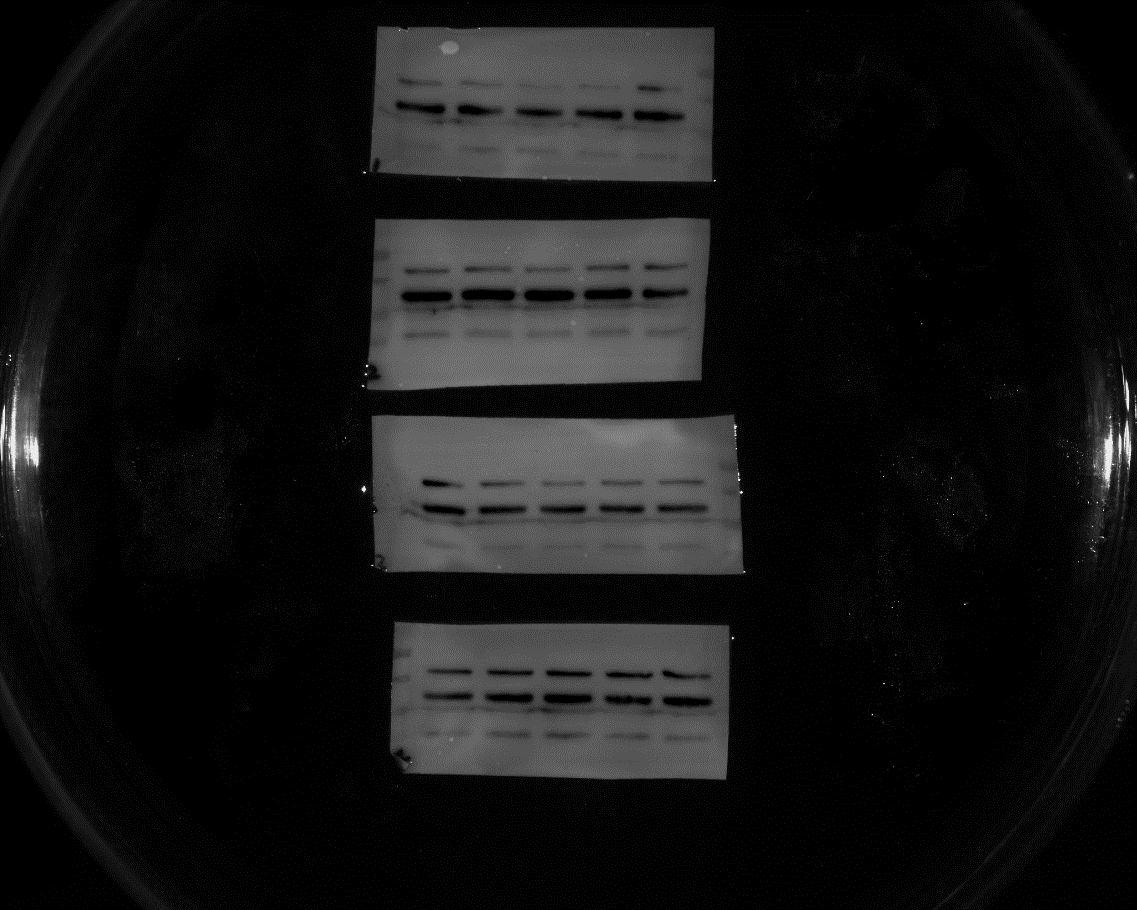


SGC-7901-PAK1


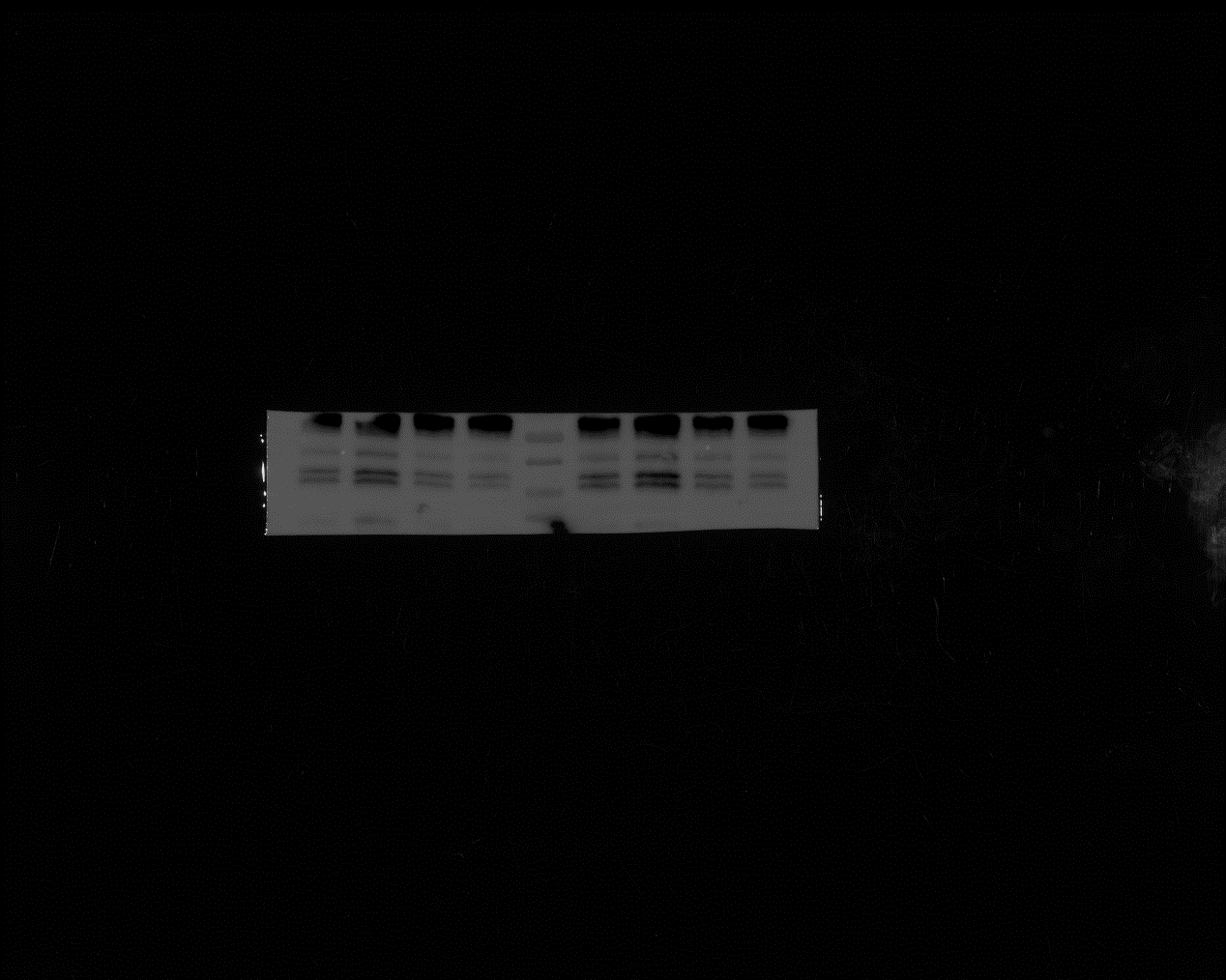


SGC-7901-p-AKT


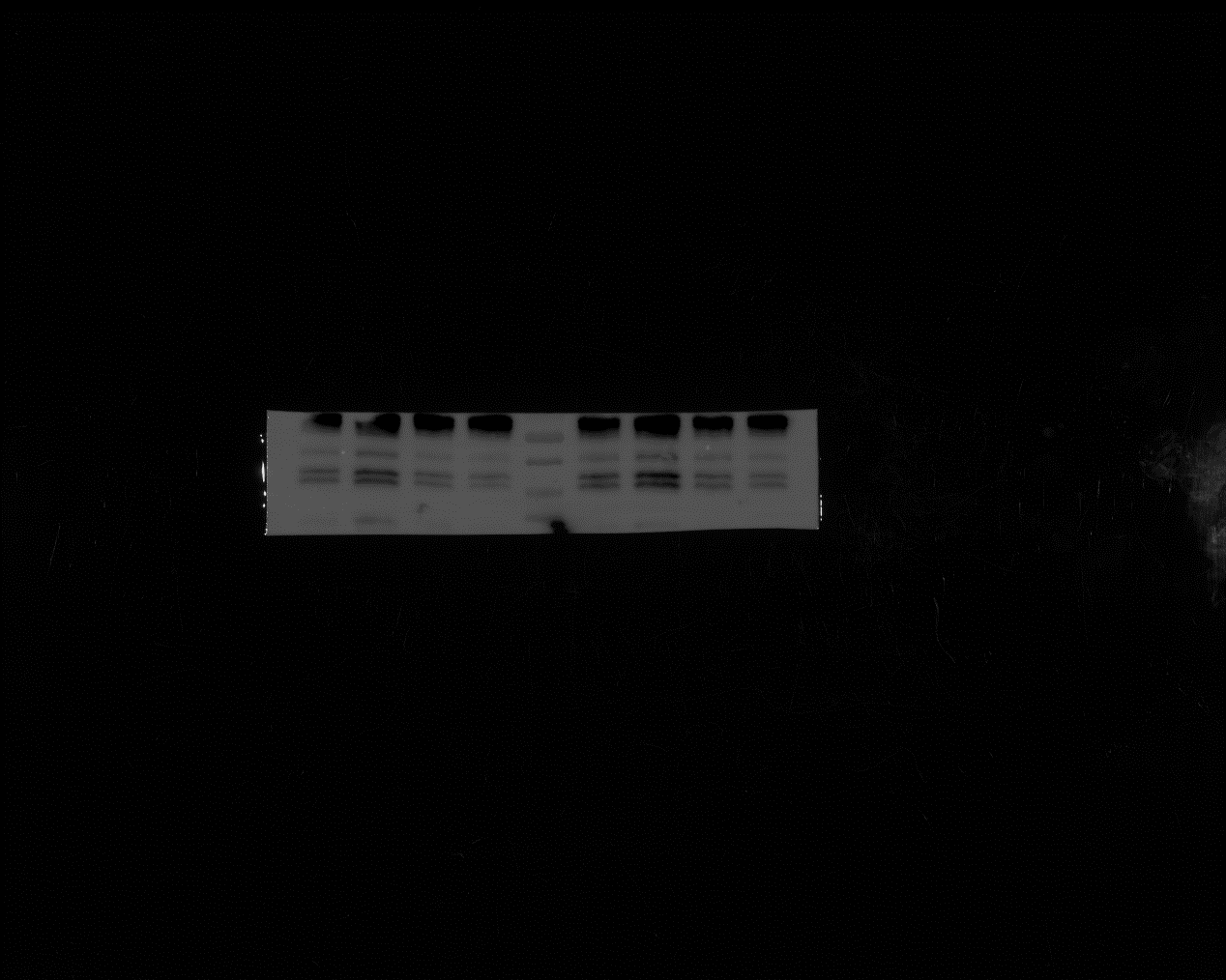


SGC-7901-AKT


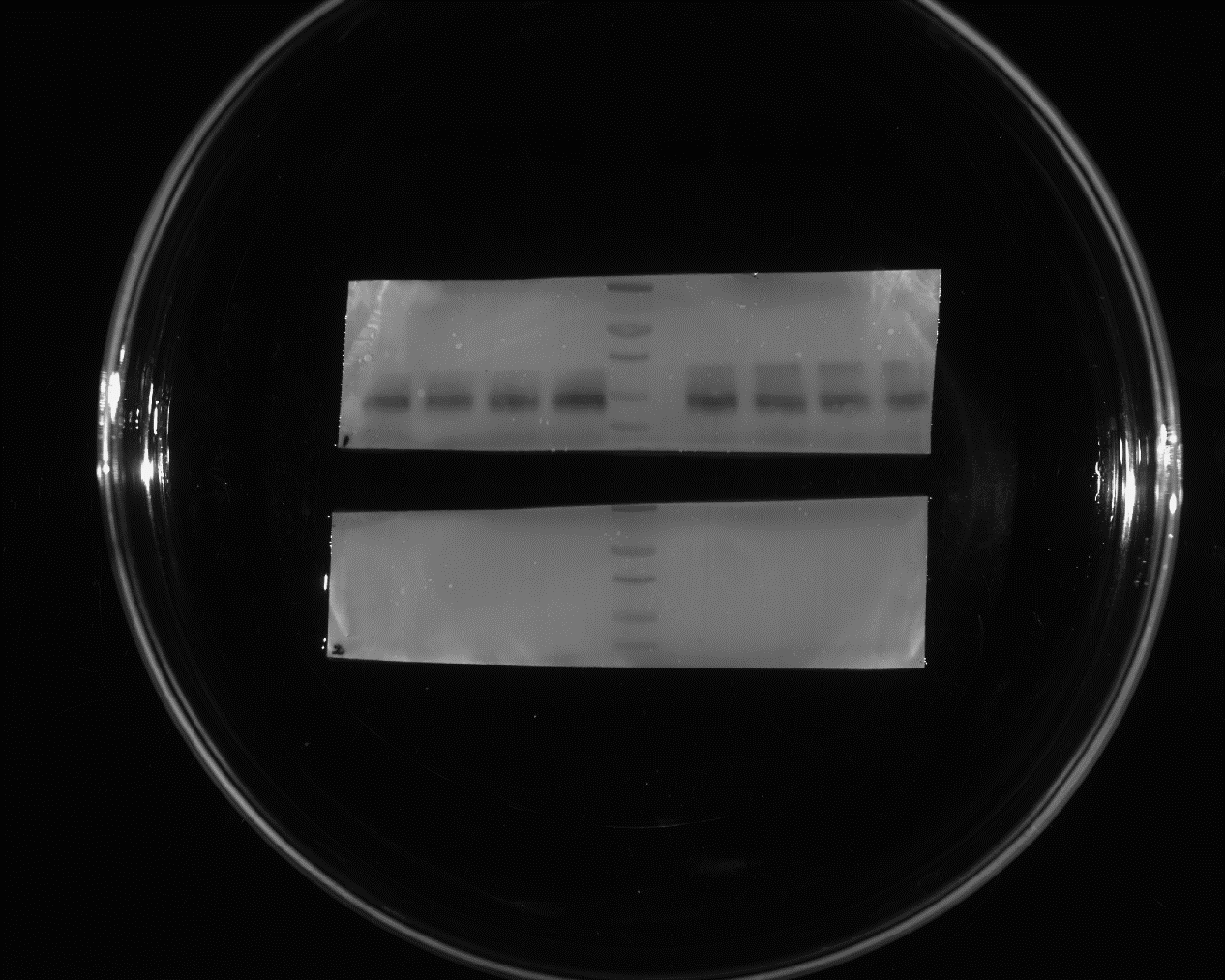


SGC-7901-p-ERK


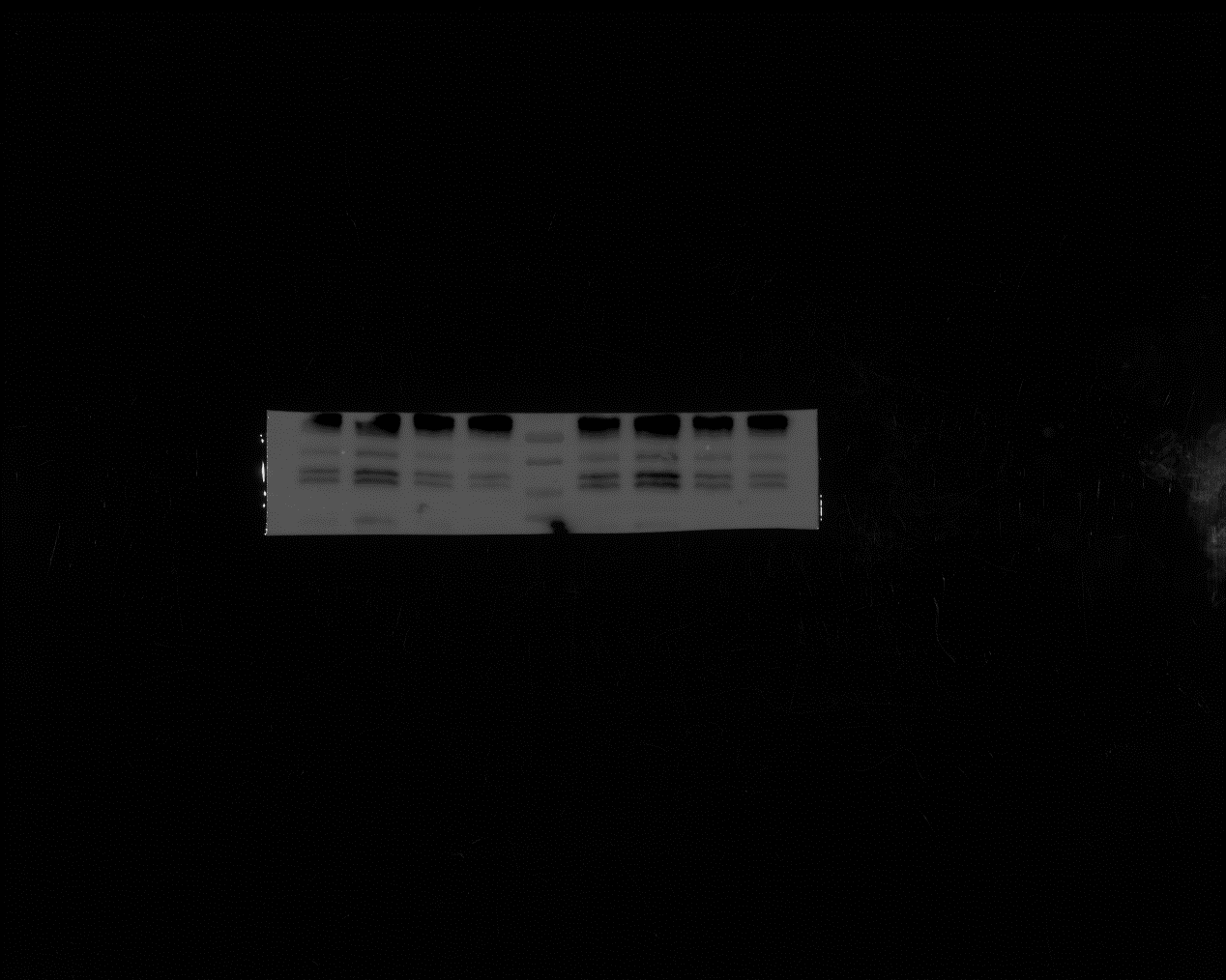


SGC-7901-ERK


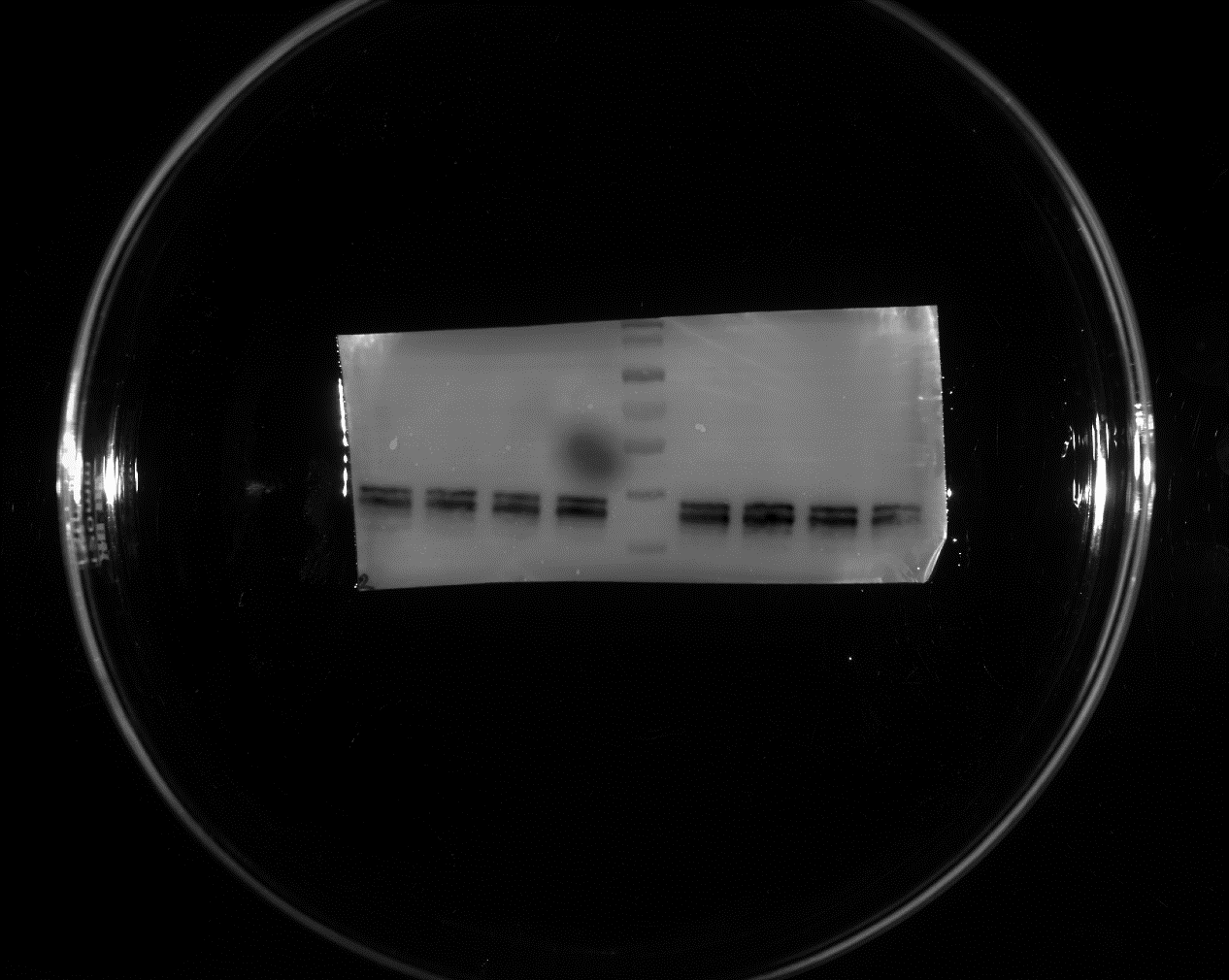


SGC-7901-Actin


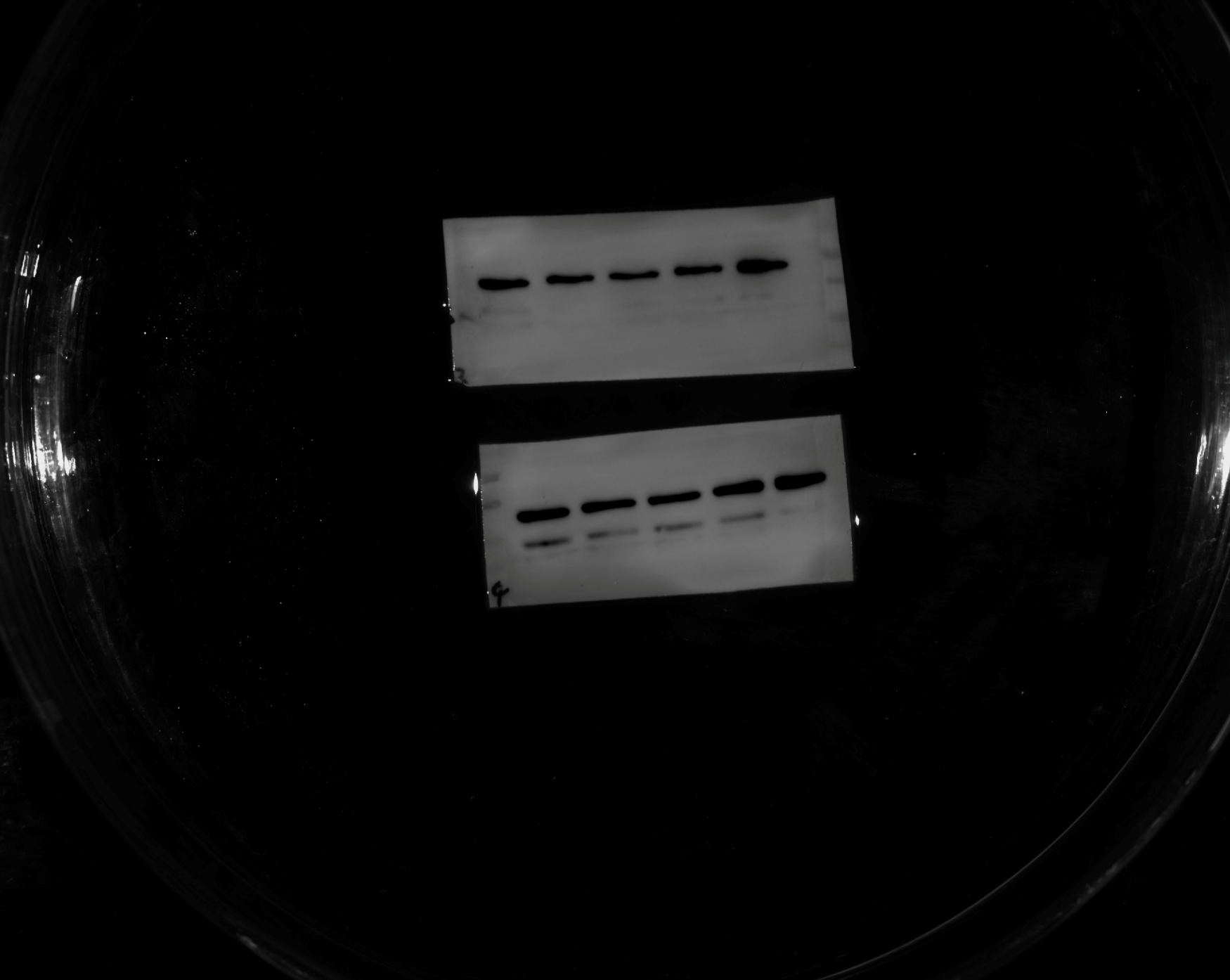


FIGURE 8F

AGS-RUNX1


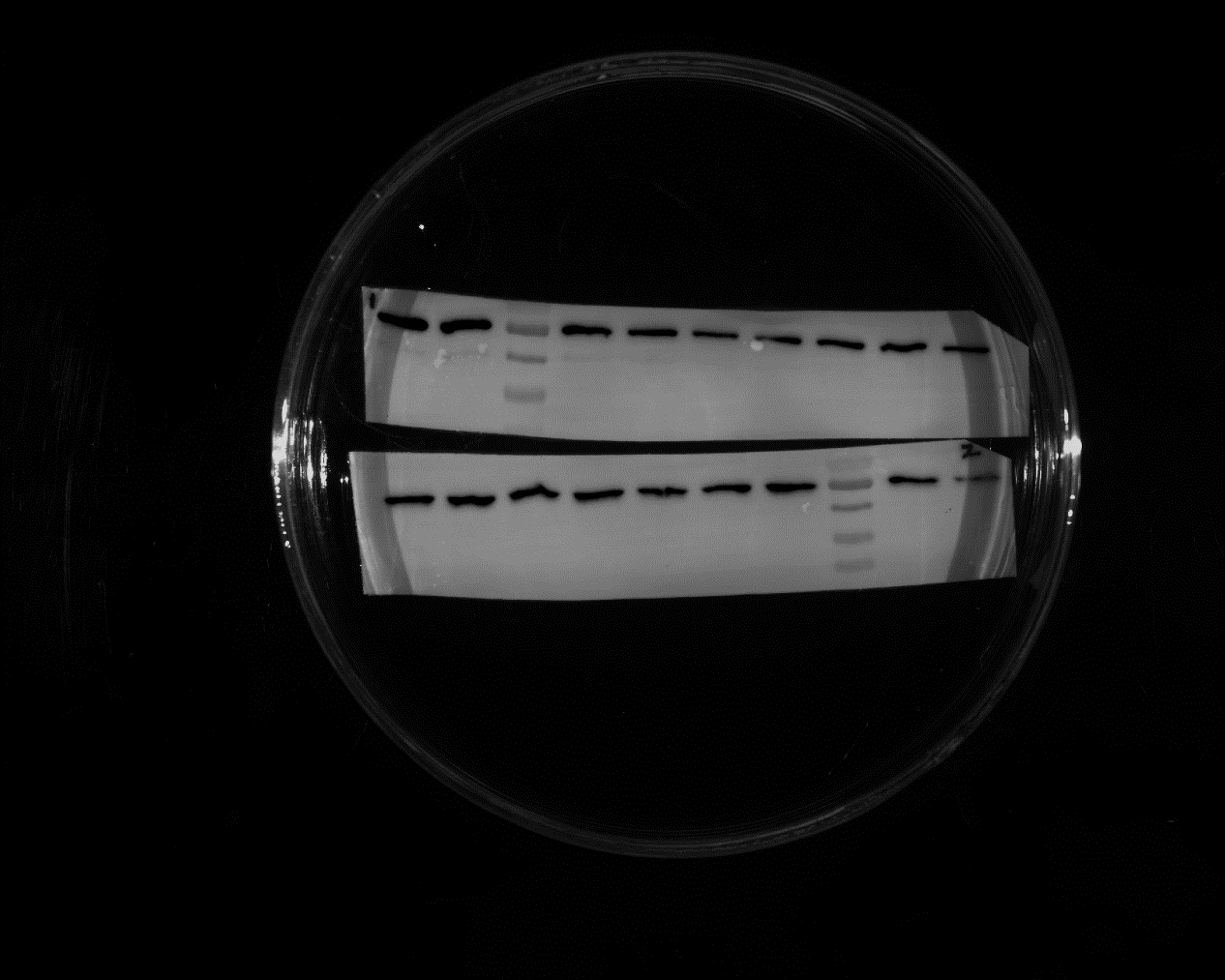


AGS-PTPN22


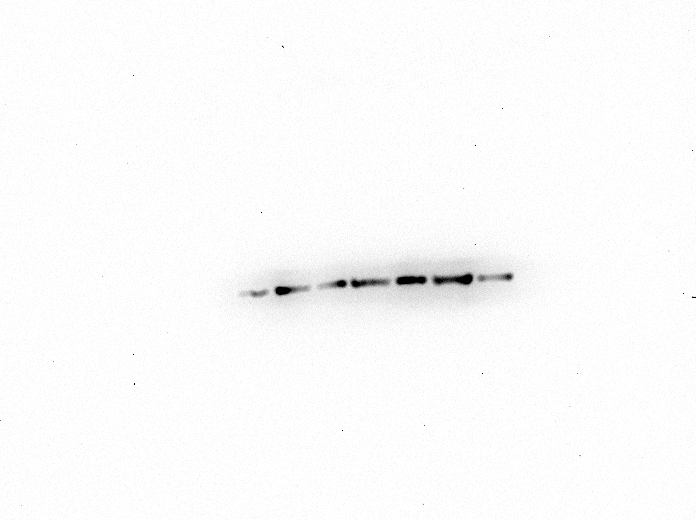


AGS-Actin


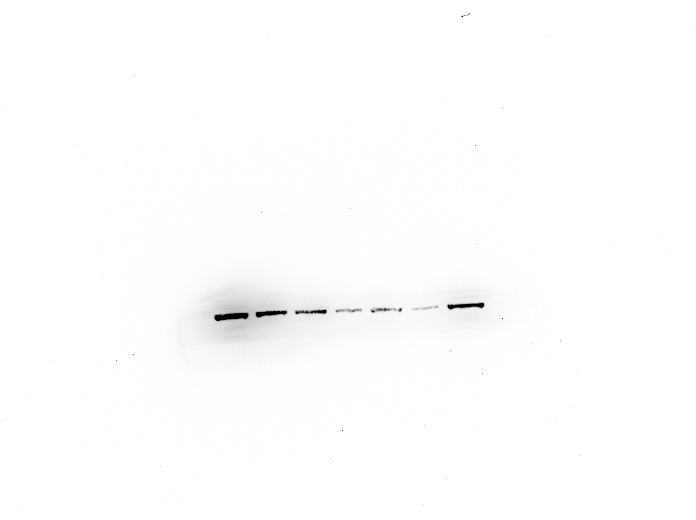


SGC-7901-RUNX1


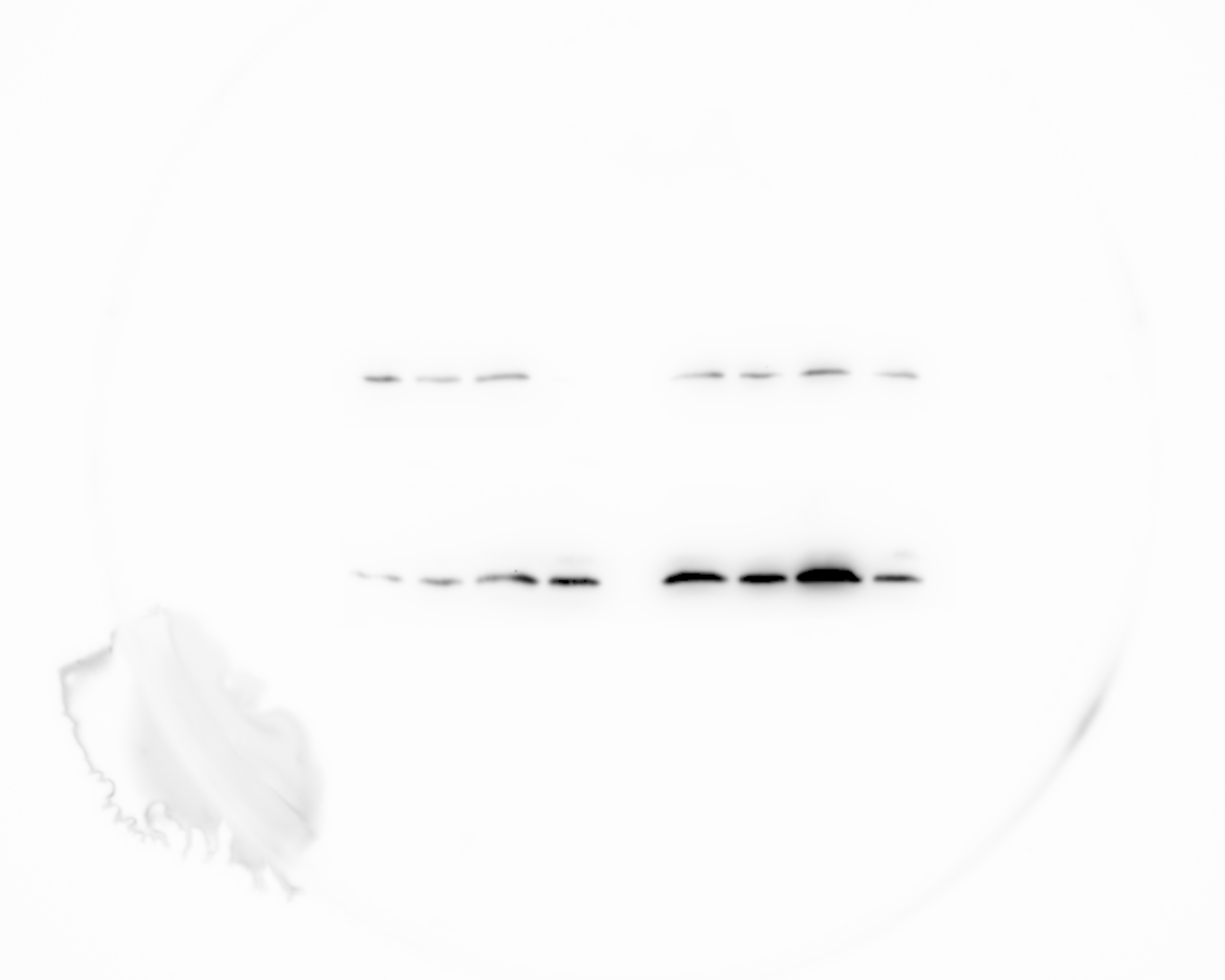


SGC-7901-PTPN22


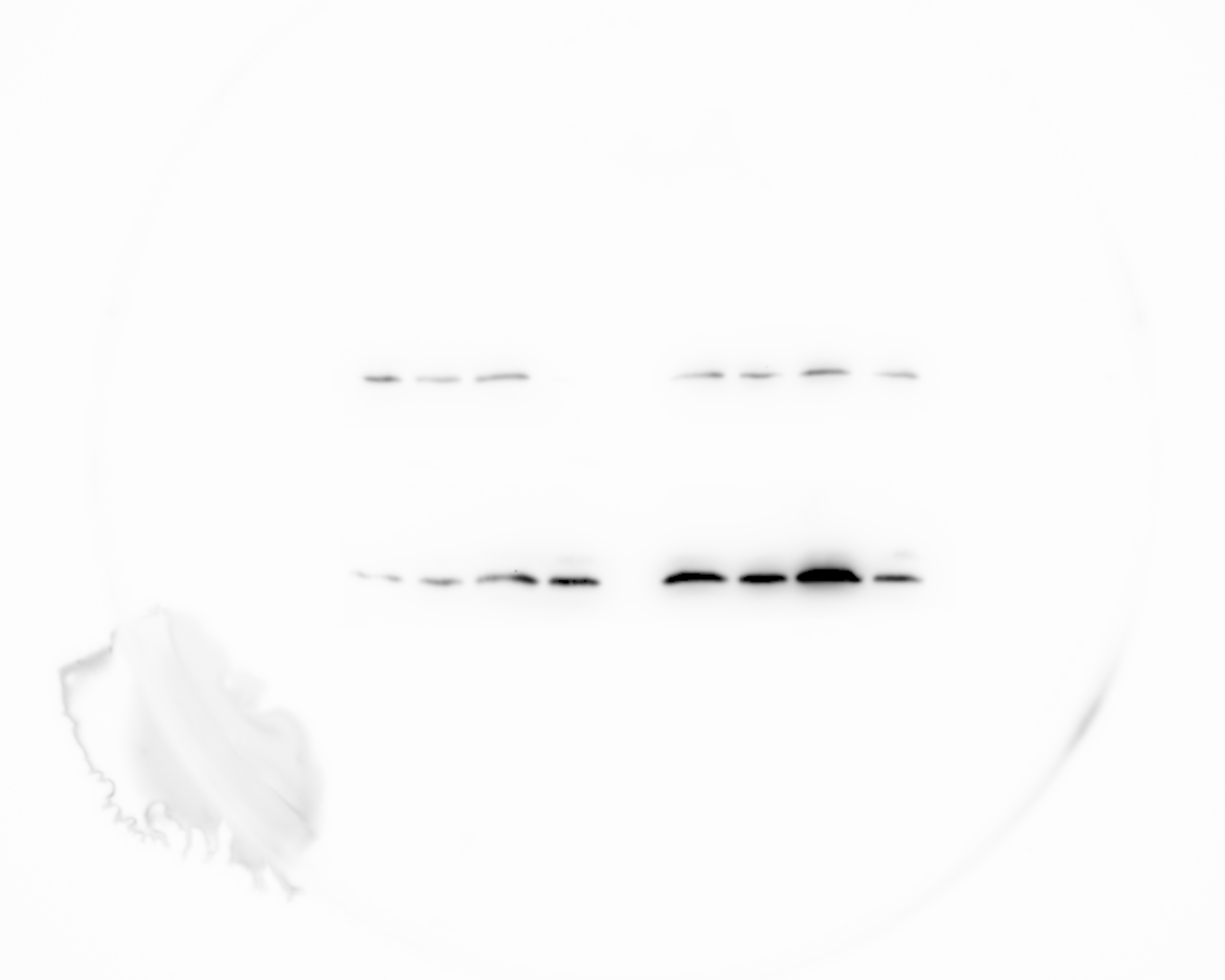


SGC-7901-Actin


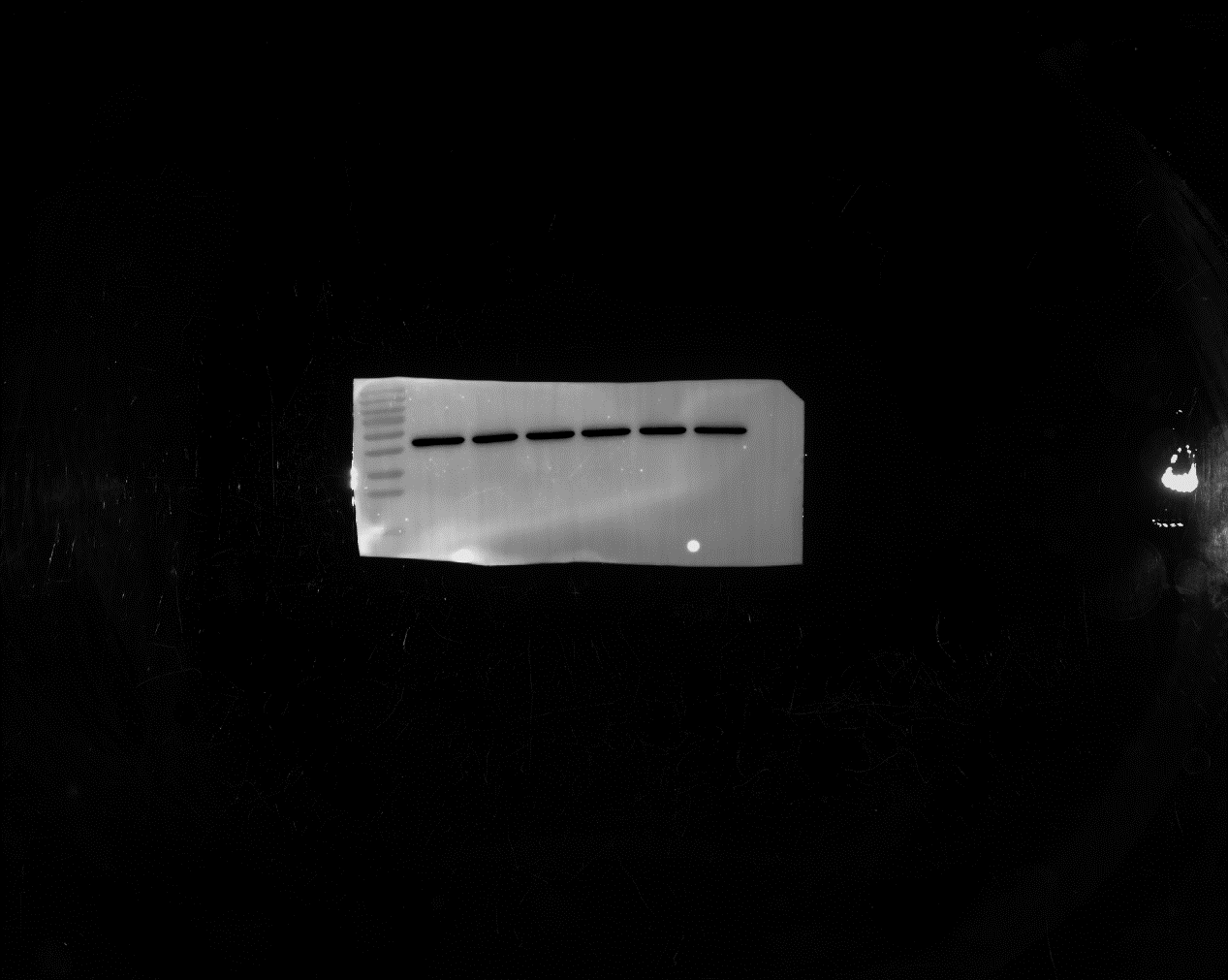


**FIGURE 8K**

AGS-E-cad


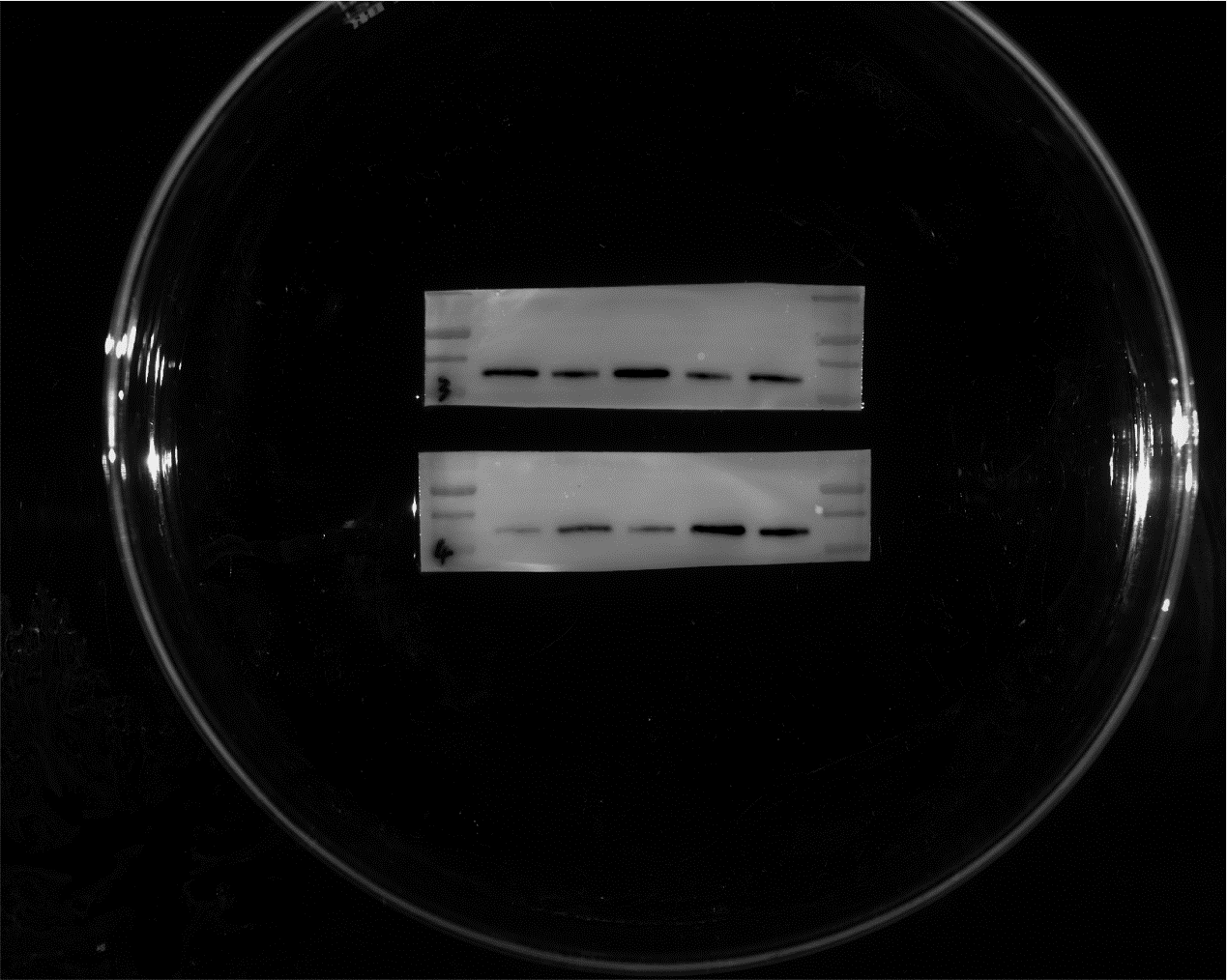


AGS-Vimentin


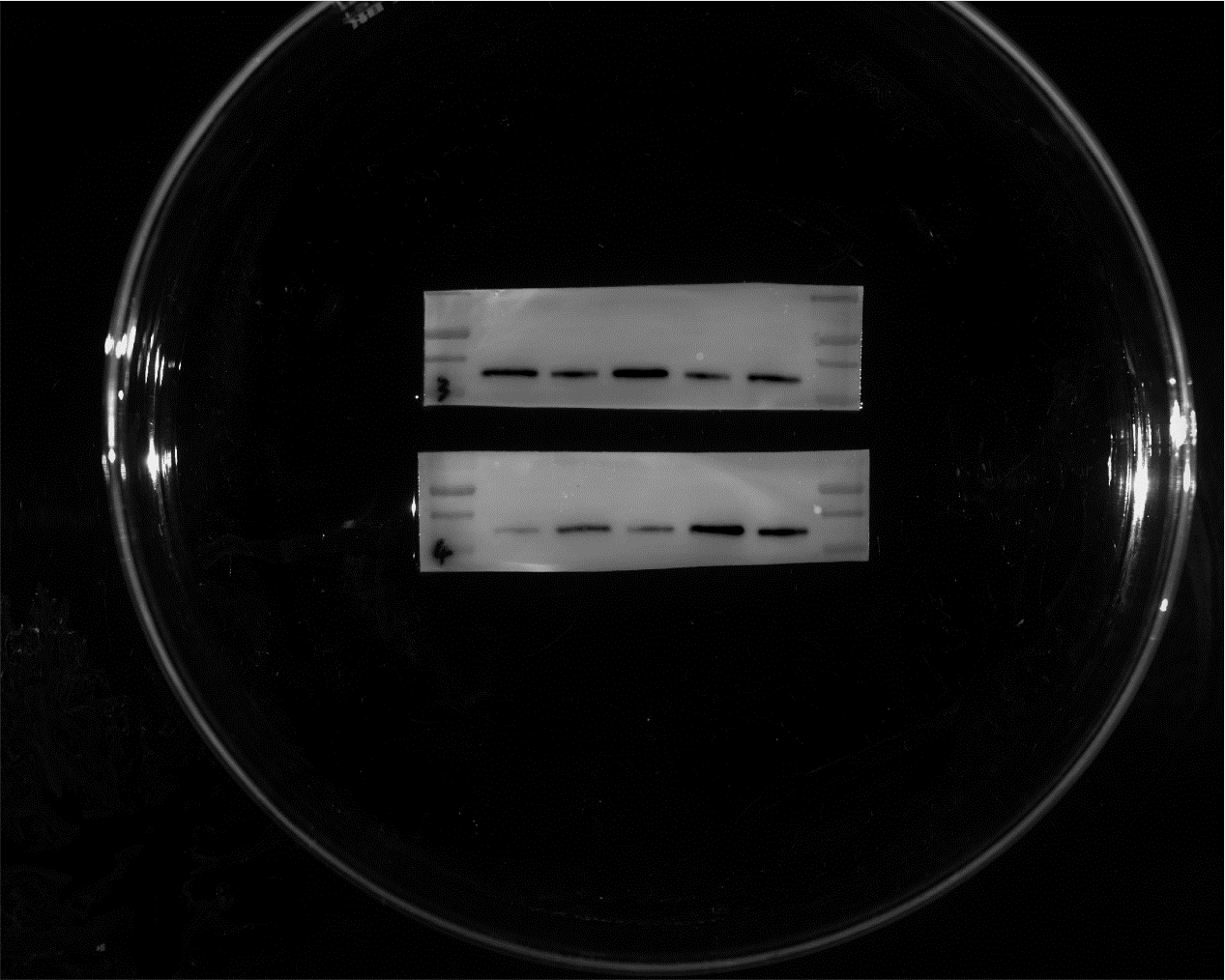


AGS-P62


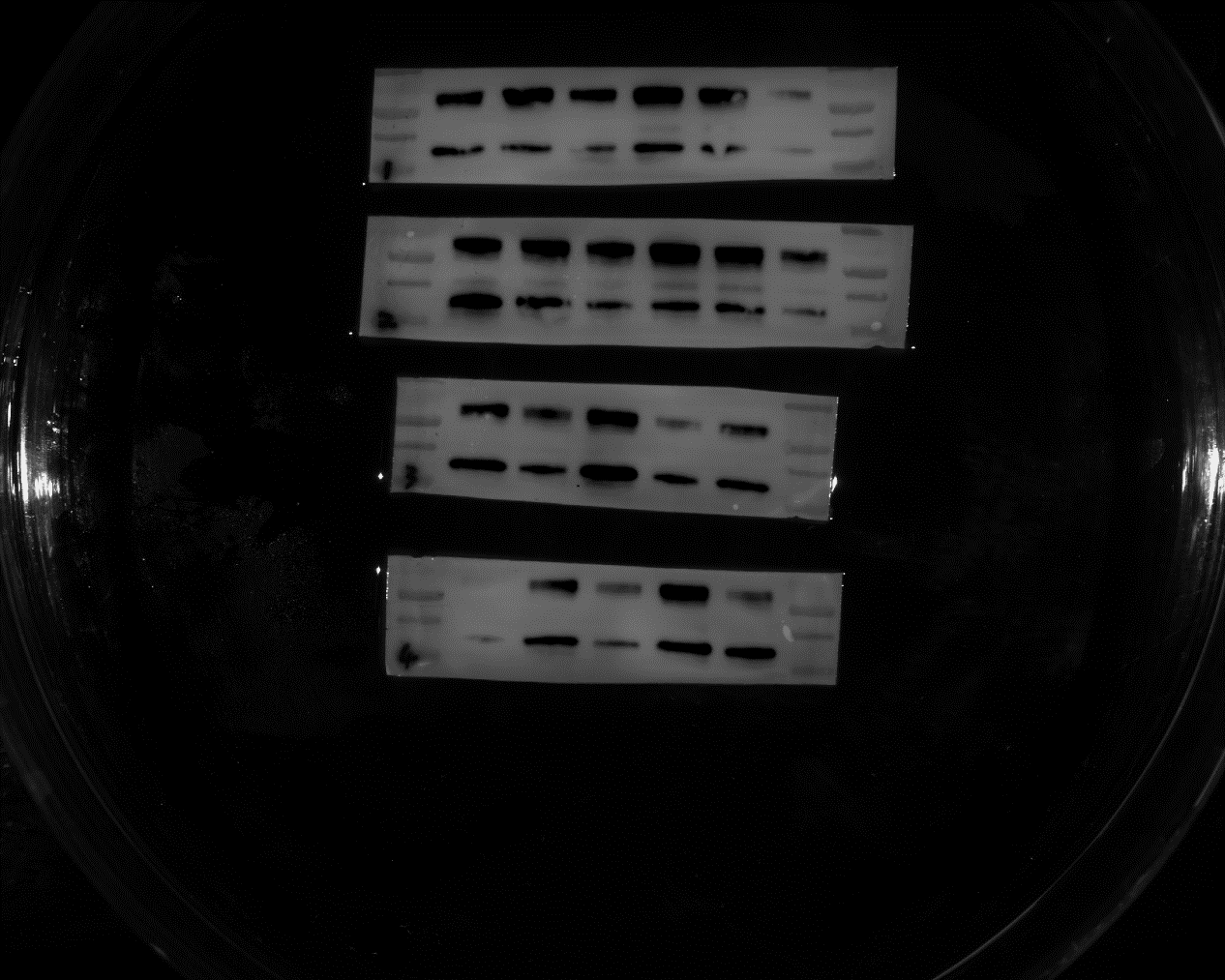


AGS-LC3


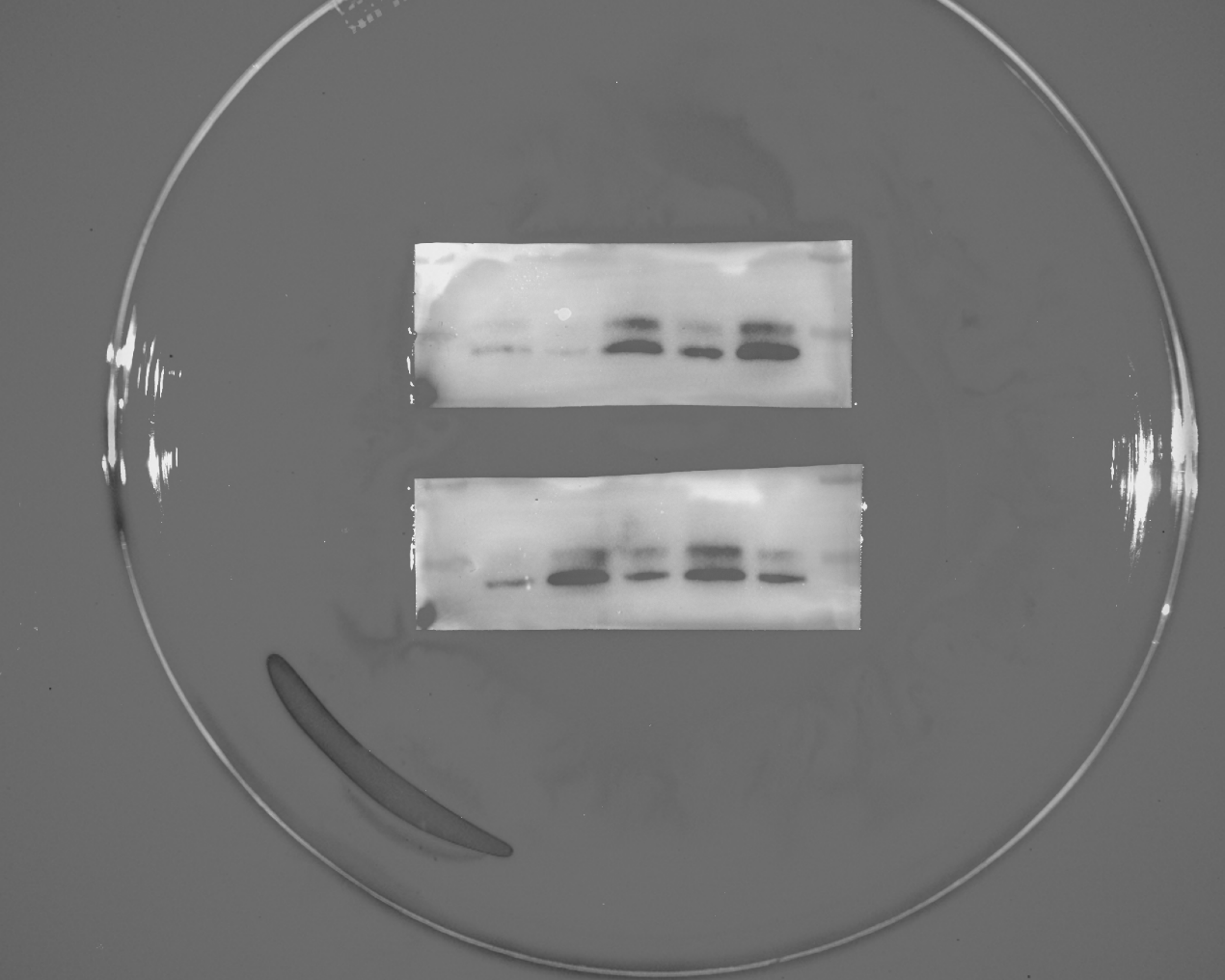


AGS-Actin


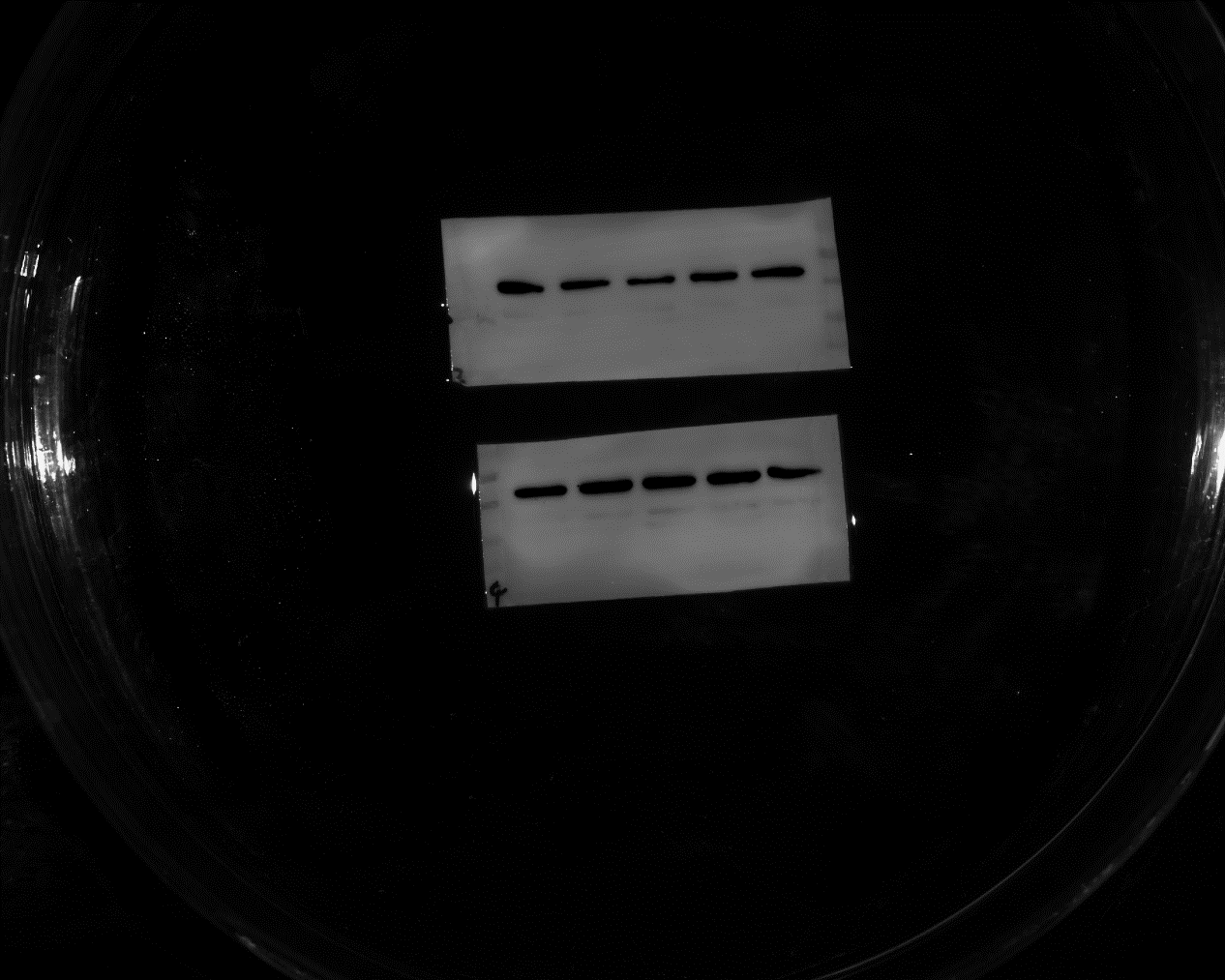


**FIGURE 9D and F**

AGS, SGC-7901-FUS


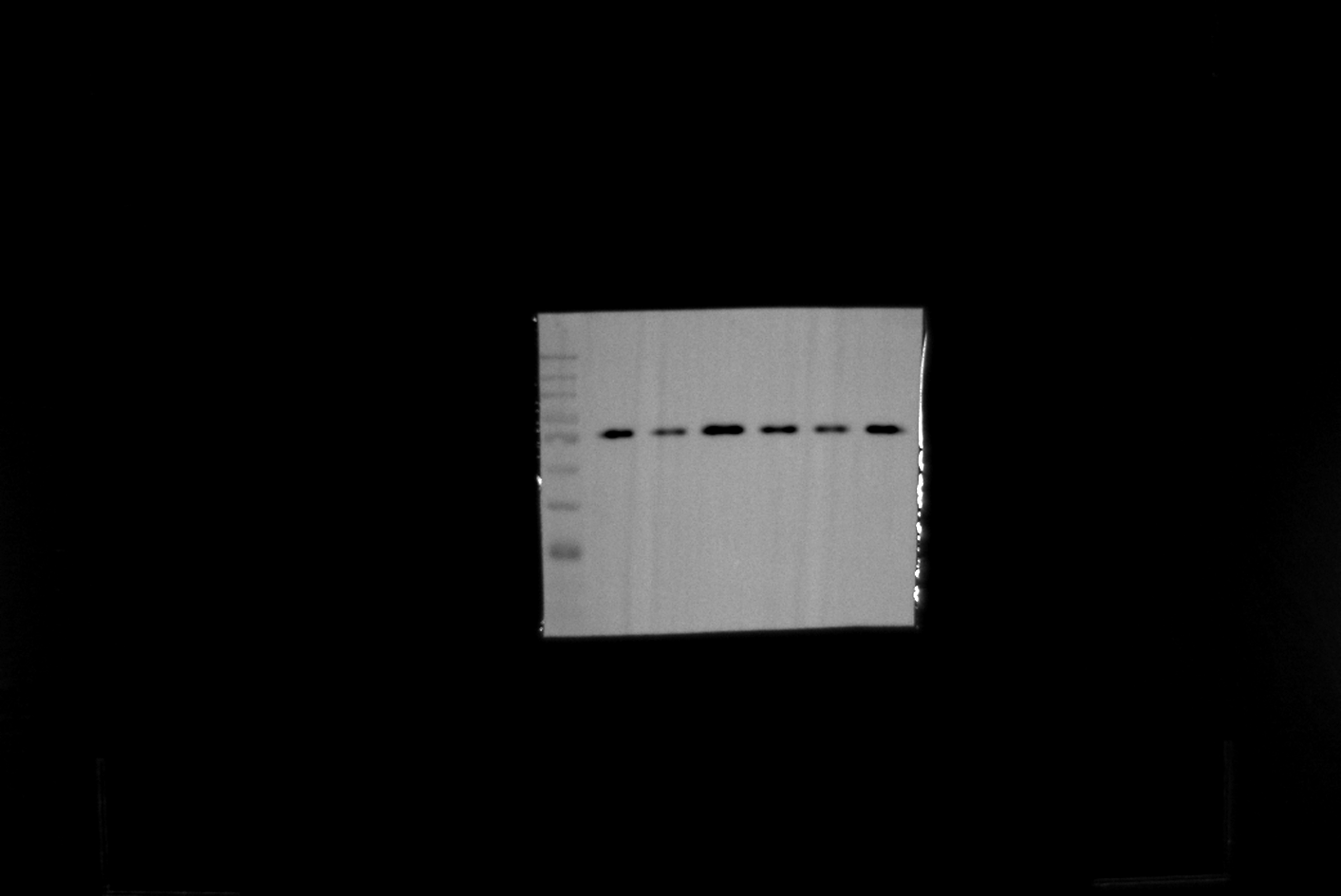


AGS, SGC-7901-RUNX1


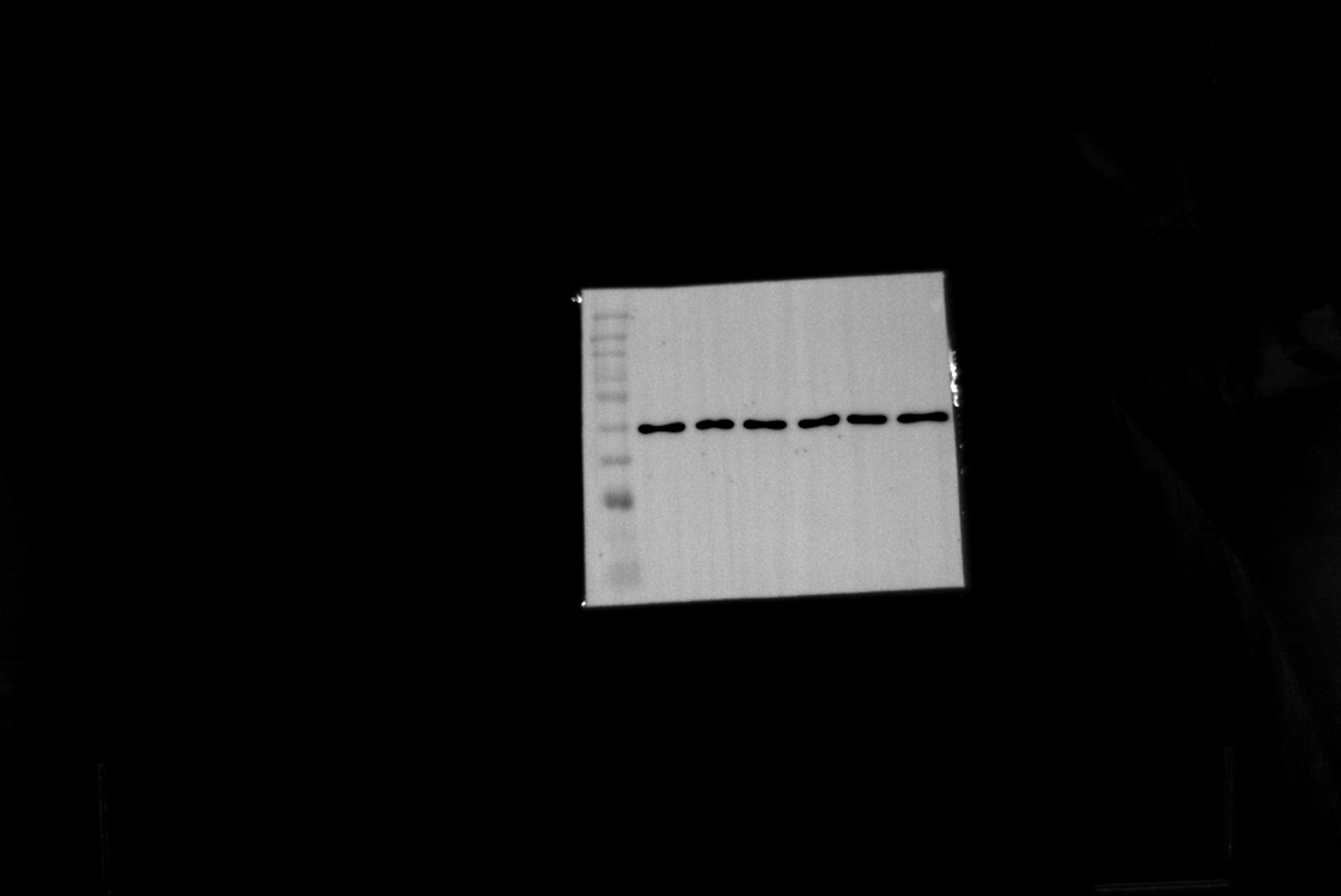


AGS, SGC-7901-ELAVL1


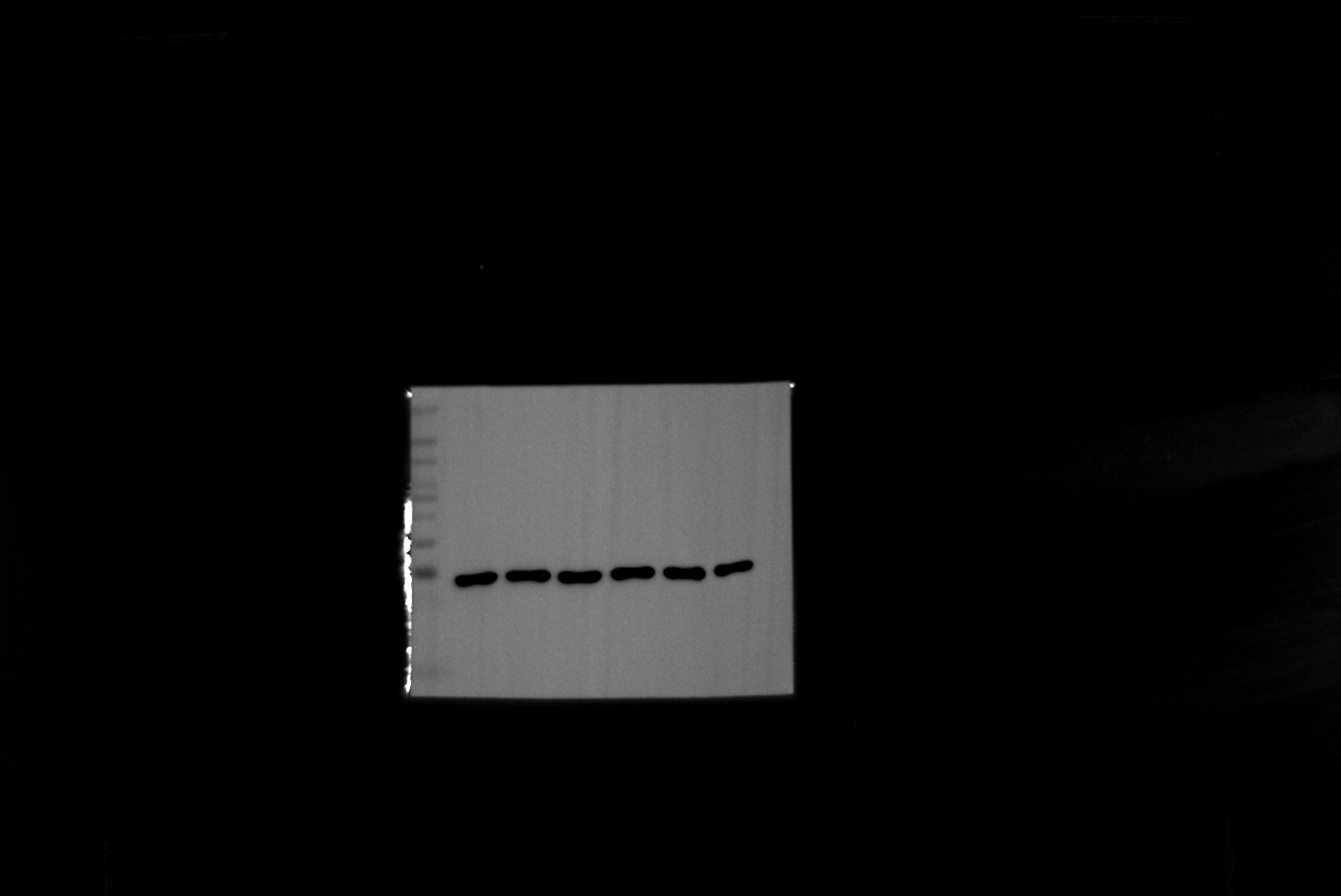


AGS, SGC-7901-β-actin


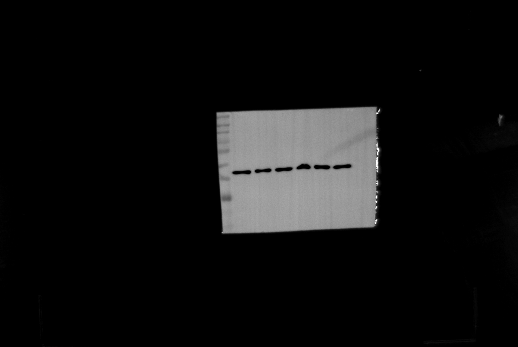

Supplement: Supplementary file 1 — Additional file 1: Table S1: The primer sequence of qRT-PCR. Table S2: Plasmid sequences were transfected. Table S3 Bioinformatics analysis was used to analyze the specific information. Table S4 Seven sites where RUNX1 binds to the promoter region of PTPN22. Figure S1. circPTPN22 can promote the proliferation, migration, and invasion of GC cells. A and B. qRT-PCR detection of circPTPN22 knockdown and overexpression efficiency in GC cells. C-H. The effect of knockdown or overexpression of circPTPN22 on the proliferation of GC cells was detected by cck-8 assay (C and D) and EdU assay (E–H). I-P. The effects of knockdown or overexpression of circPTPN22 on migration (I-L) and invasion (M-P) of GC cells were detected by transwell assay. **p < 0.01, ***p < 0.001, ****p < 0.001. Figure S2. Evidence for circPTPN22 binding to miR-6788-5p. A and B. Bioinformatics analysis of RBPs, IRES sites and ORF reading frames that circPTPN22 may bind. C. Expression of miR-6788-5p in GC cells. D. Survival analysis of GC patients in miR-6788-5p high and low groups. *p < 0.05, **p < 0.01, ****p < 0.001. Figure S3. miR-6788-5p inhibited the proliferation, migration, and invasion of GC cells. A-E. Using cck-8 (A), cell colony formation assay (B and C), and EdU cell proliferation assay (D and E) to detect the effect of adding miR-6788-5p inhibitor or mimic on the proliferation of GC cells. F and G. Transwell assay was used to detect the effect of adding miR-6788-5p inhibitor or mimic on the migration and invasion of GC cells. H. Western blot detection of E-cad and vimentin protein levels in GC cells after adding miR-6788-5p inhibitor or mimic. **p < 0.01, ***p < 0.001, ****p < 0.001. Figure S4. The expression level of PAK1 in GC cells. *p < 0.05, ****p < 0.001. Figure S5. FUS and ELAVL1 can partially restore the effects of circPTPN22 on the proliferation, migration, and invasion of GC cells. A. The expression level of RUNX1 in gastric cancer tissues. B. The expression level of FUS and ELAVL1 i [file 11658_2024_610_MOESM1_ESM.docx]
